# Soil mulching significantly enhances yields and water and nitrogen use efficiencies of maize and wheat: a meta-analysis

**DOI:** 10.1038/srep16210

**Published:** 2015-11-20

**Authors:** Wei Qin, Chunsheng Hu, Oene Oenema

**Affiliations:** 1 Key Laboratory of Agricultural Water Resources, Centre for Agricultural Resources Research, Institute of Genetics and Developmental Biology, Chinese Academy of Sciences, Shijiazhuang, Hebei, China; 2 Department of Soil Quality, Wageningen UR, 6700 AA, Wageningen, the Netherlands; 3 Alterra, Wageningen UR, PO Box 47, 6700 AA, Wageningen, the Netherlands

## Abstract

Global crop yields are limited by water and nutrient availability. Soil mulching (with plastic or straw) reduces evaporation, modifies soil temperature and thereby affects crop yields. Reported effects of mulching are sometimes contradictory, likely due to differences in climatic conditions, soil characteristics, crop species, and also water and nitrogen (N) input levels. Here we report on a meta-analysis of the effects of mulching on wheat and maize, using 1310 yield observations from 74 studies conducted in 19 countries. Our results indicate that mulching significantly increased yields, WUE (yield per unit water) and NUE (yield per unit N) by up to 60%, compared with no-mulching. Effects were larger for maize than wheat, and larger for plastic mulching than straw mulching. Interestingly, plastic mulching performed better at relatively low temperature while straw mulching showed the opposite trend. Effects of mulching also tended to decrease with increasing water input. Mulching effects were not related to soil organic matter content. In conclusion, soil mulching can significantly increase maize and wheat yields, WUE and NUE, and thereby may contribute to closing the yield gap between attainable and actual yields, especially in dryland and low nutrient input agriculture. The management of soil mulching requires site-specific knowledge.

Wheat and maize account for ~70% of the world cereal production but their yields are significantly limited by the availability of water and nutrients, especially in arid and semi-arid regions^1^,^2^,^3^,^4^. In regions with sufficient water and nutrient input, the water and nutrient use efficiencies of wheat and maize are often low due to suboptimal management^5^,^6^,^7^, which leads to large losses^8^,^9^,^10^. Forecasts project that food production, including wheat and maize, will have to double in order to feed the growing world population, now 7 billion but expected to be 9 to 10 billion in 2050^11^. This will increase the pressure on the use of our limited natural resources, such as land, water and nutrients. There is an urgent need to increase water and nutrient use efficiencies in the major cropping systems, especially in rainfed agricultural systems^12^.

Rainfed agriculture covers 80% of the world’s cultivated land, and contributes about 60% to the total crop production^13^. Low productivity in many arid and semi-arid rainfed agricultural systems is often due to degraded soil ferity and limited water and nutrient inputs. There are various options for increasing ‘crop yield per drop and bag’, such as straw mulching and plastic mulching^14^,^15^. These soil mulching management techniques can reduce evaporation and erosion, modify soil temperature, and reduce weed infestation, and thereby may lead to increases in yield, and possibly water use efficiency (WUE) and nitrogen (N) use efficiency (NUE)^14^,^15^,^16^,^17^. The effects of mulching were partially reported before in some previous studies along with other main objectives, such as the comparison between tillage and no or reduced tillage. For example, Rusinamhodzi, *et al.*^18^ assessed the effect of long-term no tillage, crop rotation and straw mulching on maize grain yield. They found that mean maize yield was ~1 ton ha^−1^ higher with conservation agriculture practices (with straw mulching) when mean annual precipitation was below 600 mm. However, when mean annual precipitation was above 1000 mm, these conservation agriculture practices may have lower yields (~1 ton ha^−1^). Recently, Pittelkow, *et al.*^19^ reported that crop yields increased by 7.3% under rainfed agriculture in dry climates when no-tillage, straw mulching and crop rotation are implemented together. No-till applied alone (without straw mulching and crop rotation) reduced yields by 11.9%. Also, effects of no-tillage with or without mulching were larger in dry conditions than humid conditions^19^. Others found that straw mulching may retard seed germination and early growth of crops, especially in relatively cold climatic conditions^20^. Currently, plastic films are widely used in some regions such as China and India, mainly because of governmental subsidies. Plastic films are more effective in reducing soil evaporation compared to straw mulching, but large amounts of plastic film residual may have negative effects on soil structure, water and nutrient transport and crop growth, thereby reducing crop production^21^. Hence, the reported effects of mulching often differ and sometimes contradict between studies, likely due to differences in the climatic conditions (rainfall and temperature), soil characteristics, crop species, and also water and N input levels. As yet, a systematic and quantitative assessment of the effects of soil mulching on crop yields, WUE and NUE as function of environmental conditions has not been carried out.

Here, we examine the effects of straw and plastic mulching on yield, WUE and NUE of wheat and maize, as function of environmental conditions using a meta-analysis of published results. We selected wheat and maize as test crops because of their global importance, and their contrasting responses to environmental conditions. A comprehensive and quantitative understanding of the effects of mulching may contribute to closing yield gaps between attainable and actual crop yields, and to guiding practitioners better. The objectives of our study were (1) to examine the effects of mulching on wheat and maize yield, WUE and NUE on the basis of results of published studies; (2) to relate variations in the effects of mulching to variations in inputs of water and N, temperature, and to soil organic matter; and (3) to quantify possible interactions between water and N use in yield, WUE and NUE.

## Methods

### Data collection

We searched in peer-reviewed literature for publications investigating the effects of mulching on yield of maize and wheat using Scopus (Elsevier). Search terms included ‘mulch’ and/or ‘mulching’, ‘maize’ and/or ‘wheat’, ‘yield’, ‘water’ or ‘nitrogen’ in the article title, abstract, and keywords. Conference proceedings and non-English language publications were excluded. This search produced a total of ~600 publications, which were screened on the basis of the following criteria: (1) studies must contain both no-mulching and mulching treatments (either straw or plastic mulching); (2) crop yields, water input and N input were all reported so that the interactions between water and N can be quantified; (3) location, year and soil information of the experiment was stated. The final analysis was based on 1310 yield observations from 74 studies conducted in 19 countries (Table S1).

### Definitions and data analysis

Water use efficiency (WUE, in kg m^−3^) was defined as:


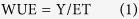


where Y is yield (in kg ha^−1^), ET is evapotranspiration (mm, m^3^ ha^−1^) reported in the study. Because most of the studies were conducted in water-limited environment where ET is closely related to total water (rainfall + irrigation) input, when ET was not reported in the studies, we considered ET is equal to total water (rainfall + irrigation) input (mm, m^3^ ha^−1^) during the crop growing season.

Nitrogen (N) use efficiency (NUE, dimensionless, or kg kg^−1^) was defined as:


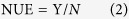


where N is the total N input from fertilizer and/or manure, all converted to N content (kg ha^−1^). The N input via the straw for soil mulching was small (<20 kg N ha^−1^) and therefore neglected. A few observations (75) with zero (0) N input were excluded from the final dataset to avoid errors (non-values) in the calculation of NUE.

The magnitude of the mulching effects on yield in each study was calculated as the natural logarithm of the response ratio (R)^22^:


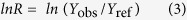


where Y_obs_ is the observed yield of the mulching treatment, Y_ref_ is the mean yield of no-mulching treatment, and the reference to be compared with. Hence, the comparisons were side-by-side and equal weight was given to each calculated effect size.

In meta-analysis studies, the observations can be weighted by many ways. When studies did not report standard deviation or standard error, the observations can be weighted equally (or unweighted), weighted with sample size of the study or weighted with number of replicates of control and treatment group^22^,^23^,^24^. We tested and compared three different weighting methods for each observation. In the end, we chose the equal weight method for each observation because this method produced the smallest AIC (Akaike information criterion) value, among all three weighting methods (Table S2).

We used the mean yield of no-mulching treatment(s) as the reference because of the following reasons. Although most of studies included in our dataset match the criteria of side-by-side comparison between mulching and no-mulching, there were still some studies which were not specifically designed for testing the effects of mulching. For example, there can be multiple observations with no-mulching treatments, mostly with different tillage (no tillage, reduced tillage or conventional tillage) and/or land preparation (flattening the field or making ridges and furrows). This then may lead to multiple comparisons with no-mulching treatments. In the case that the mulching treatment was compared to a no-mulching treatment with low yield, the effects of mulching would be overestimated. The opposite is also true, i.e., if the mulching treatment was compared to a no-mulching treatment with high yield, the effects of mulching would be underestimated. To avoid either over- or under-estimation of the mulching effect, we decided to use the mean yield from no-mulching treatments as reference, and then compare the observed yield of the mulching treatments to the reference, to derive the response ratio. The same approach was applied to the calculation of the effect sizes of WUE and NUE.

The effect size (ln R) was statistically analyzed with a mixed-effect model via the R package “nlme”^25^,^26^:


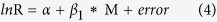


where α is the intercept with the same dimension as *ln R*, *β*_1_ represent the response due to the mulching treatment (M) and *error* represents the residual that was not explained by the mulching variable. In this mixed-effect model, mulching treatments (i.e., no-mulching, straw mulching, and plastic mulching) were set as fixed effects and studies were set as random effects.

To investigate how the effects of mulching varied due to the levels of water input, N input and temperature, the whole dataset was separated into two sub-datasets according to the 50^th^ percentile value of the water input, N input, temperature and soil organic matter (SOM) content, respectively. Mean effects of mulching were considered significant if confidence intervals did not overlap with 0 (P values = 0.05). Mean effects for different subgroups were considered to be significantly different from one another if their 95% confidence intervals did not overlap. For ease of interpretation, all results were back-transformed and reported as percentage change in yield (and in WUE and NUE) for each mulching treatment. The analysis was conducted for wheat and maize separately. A similar procedure has been used in some recent meta-analysis studies^19^,^23^,^27^.

Also, the overall mean effects of multiple variables and possible interactions between variables, including mulching, soil organic matter, temperature, water and N input on yield (and on WUE and NUE) were analysed, using the sub-datasets for wheat and maize, with the following formula:


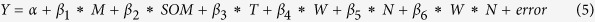


where *Y* is yield (in ton ha^−1^), *β*_1–6_ represent the response due to changes in each variable, M is mulching treatment, T is mean air temperature during the growing season (°C), W is mean water input (rainfall + irrigation) during the growing season (m^−3^ ha^−1^), and N is fertilizer and/or manure N input (kg ha^−1^), among which only M is a discrete variable and all others are continuous variables. Note that there are differences in the interpretation of *β* related to discrete and continuous variables. For the mulching treatment (*M*), *β*_1_ is interpreted as the change in yield in absolute terms, compared to the intercept (α) with no-mulching (the reference). For the continuous variables, *β*_2−6_ is interpreted as the response in yield due to per unit change in the variable. Note also that the response variable in Eq 5 is actual yield (and in Eq. 4 is *ln* R).

## Results

### Overview of the dataset

Our dataset consisted of 1310 yield observations from 74 studies conducted in 19 countries (Table S1). There were 569 observations for wheat (CK: 208, Straw: 289, Plastic: 72) and 741 observations for maize (CK: 270, Straw: 328, Plastic: 143). So there are more observations for maize than wheat, and more observations for straw mulching than plastic mulching. Wheat received relatively less water compared to maize. The water input of wheat ranged from 25 to 1000 mm, and that of maize ranged from 150–2000 mm. However, most of the observations were concentrated below 800 mm. Wheat also received relatively less N input, compared to maize. The N input of wheat ranged from 20–200 kg N ha^−1^, and that of maize ranged from 30–400 kg N ha^−1^. The 25^th^ and 75^th^ percentile values indicate that wheat yields ranged from 2.5 to 7.0 ton ha^−1^ and maize yields ranged from 2.5 to 10 ton ha^−1^; WUE of wheat ranged from 0.5 to 1.5 kg m^−3^ and WUE of maize ranged from 0.5 to 2.5 kg m^−3^; NUE of wheat ranged from 20 to 50 kg kg^−1^ and NUE of maize from 20 to 80 kg kg^−1^.

### Overall effects of mulching on yields, WUE and NUE

**Figure 1:**
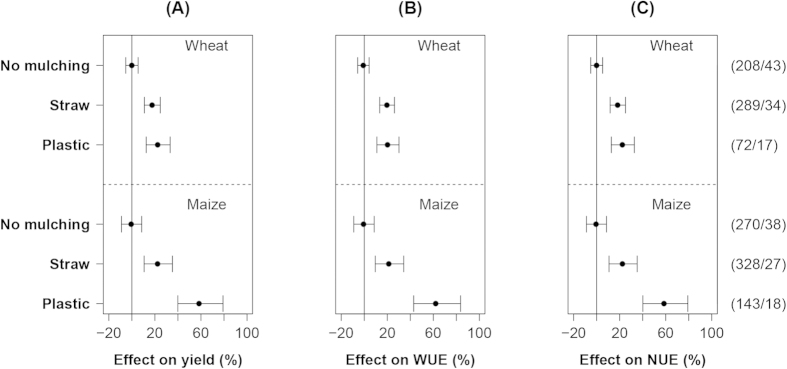
Effect of mulching on crop yield (**A**), water use efficiency (**B**) and nitrogen use efficiency (**C**) of wheat (upper panels) and maize (lower panels). Dots show means, error bars represent 95% confidence intervals. The number of observations and total number of studies for each treatment are displayed in parentheses on the right-hand side of the figure, respectively.

Yields, WUE and NUE of wheat and maize were significantly increased by both straw mulching and plastic cover, compared to the reference value with no-mulching (Fig. 1). The mulching effects on yield, WUE and NUE were highly similar because WUE and NUE were calculated as yield per water/N input. On average, straw mulching and plastic mulching increased yield, WUE and NUE of wheat by 20%. The mean effect of straw mulching on maize were similar to that of wheat, but plastic mulching increased yield, WUE and NUE of maize by ~60%.

### Effects of mulching on yields, WUE and NUE at different water input levels

**Figure 2:**
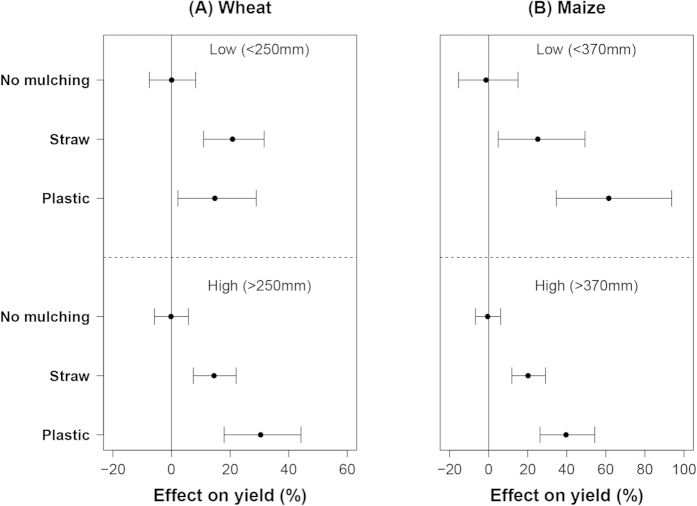
The effect of mulching on wheat (**A**) and maize (**B**) yields at different water input levels. Data were sub-grouped according to the 0.5 quantile value of the water input of each crop with plastic mulching treatments and displayed as low and high (from top to the bottom).

The effects of mulching were affected by water input. Here the dataset was separated into just two sub-datasets according to the 50^th^ percentile value of the water input of each crop with plastic mulching treatments (because of relatively small number of observations, and in order to have reasonable comparisons). The mean effect of straw mulching on wheat yields was 20% at low water input level (<250 mm) and 15% at high water input (>250 mm). In contrast, the mean effect of plastic mulching on wheat yields was 15% at low water input and 35% at high water input (Fig. 2A). For maize the mean effect of straw mulching on maize yield was 20%, independent of water input level. The mean effect of plastic mulching on maize yield was 60% at low water input (<370 mm) and 40% at high water input (Fig. 2B). Interestingly, the confidence intervals (CI) of the mulching effects on maize yields also decreased with increased water input. For example, the CIs of straw mulching were ±18% at low water input and decreased to ±8% at high water input.

**Figure 3:**
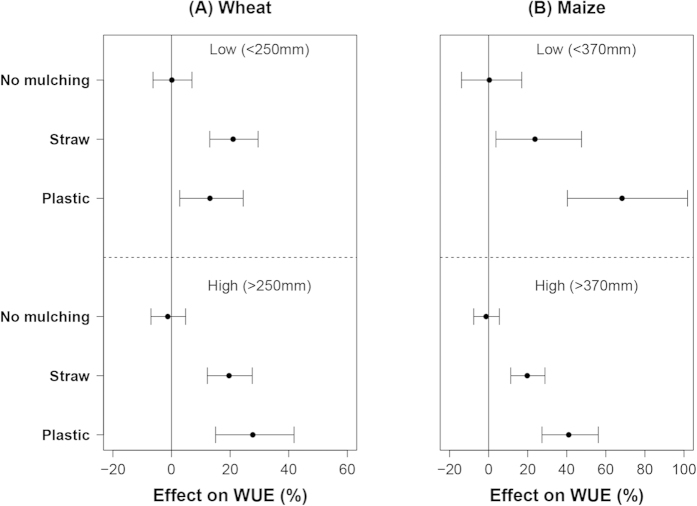
The effect of mulching on WUE of wheat (**A**) and maize (**B**) at different water input levels. Data were sub-grouped according to the 0.5 quantile value of the water input of each crop with plastic mulching treatments and displayed as low and high (from top to the bottom).

The mean effect (20%) of straw mulching on WUE of wheat was not affected by water input, but the mean effect of plastic mulching was 15% at low water input and 28% at high water input (Fig. 3A). For maize, the mean effect (20%) of straw mulching on WUE was also independent of water input level, the mean effect of plastic was 70% at low water input and 40% at high water input (Fig. 3B). The CIs of the mulching effects on WUE of maize also decreased with increased water input.

**Figure 4:**
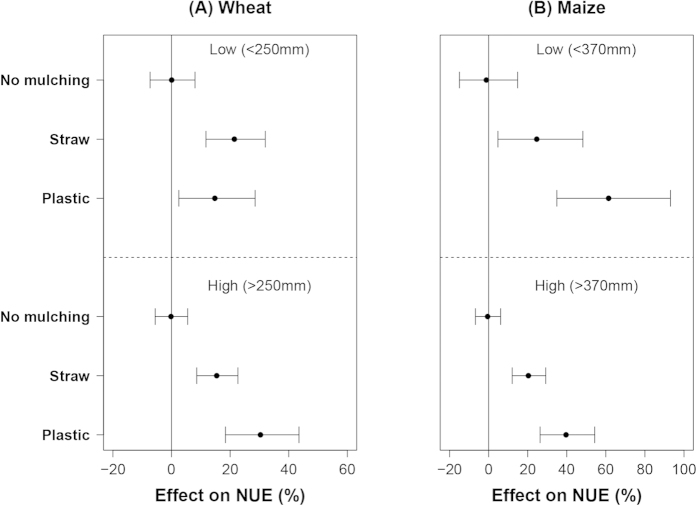
The effect of mulching on NUE of wheat (**A**) and maize (**B**) at different water input levels. Data were sub-grouped according to the 0.5 quantile value of the water input of each crop with plastic mulching treatments and displayed as low and high (from top to the bottom).

The mean effects of straw mulching on NUE of wheat were slightly higher at low water input than at high water input. Mean effects of plastic mulching on NUE were lower at low water input than at high water input (Fig. 4A). For maize, the mean effect of straw mulching on NUE was around 20%, independent of water input level, whereas that of plastic mulching decreased from 60% at low water input level to 40% at high water input level (Fig. 4B).

### Effects of mulching on yields, WUE and NUE at different N input levels

**Figure 5:**
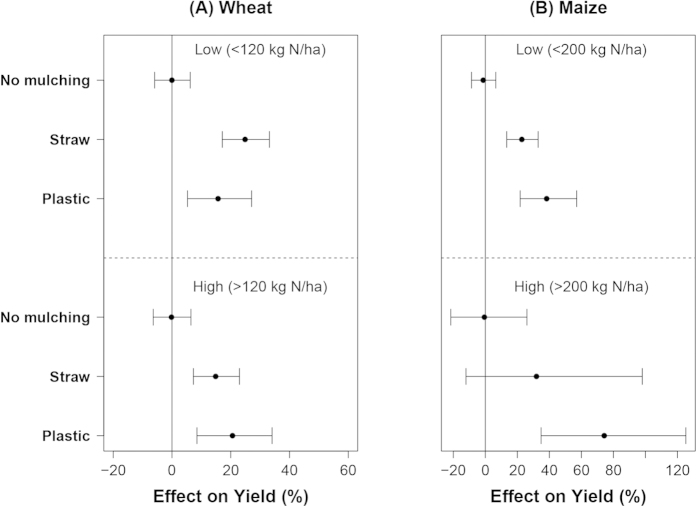
The effect of mulching on wheat (**A**) and maize (**B**) yields at different N input levels. Data were sub-grouped according to the 0.5 quantile value of the N input of each crop with plastic mulching treatments and displayed as low and high (from top to the bottom).

The mulching effects were also affected by N input level. Again, the dataset was separated into two sub-datasets according to the 50^th^ percentile value of the N input for each crop. The mean effect of straw mulching on wheat yield was 25% at low N input (<120 kg N ha^−1^) and 15% at high N input level. For plastic mulching we observed opposite trends (Fig. 5A). For maize, the effects of mulching was larger at high input level (>200 kg N ha^−1^), but CIs were also larger at high N input. The mean effect of plastic mulching was 75% at high N input and 40% at low N input (Fig. 5B).

**Figure 6:**
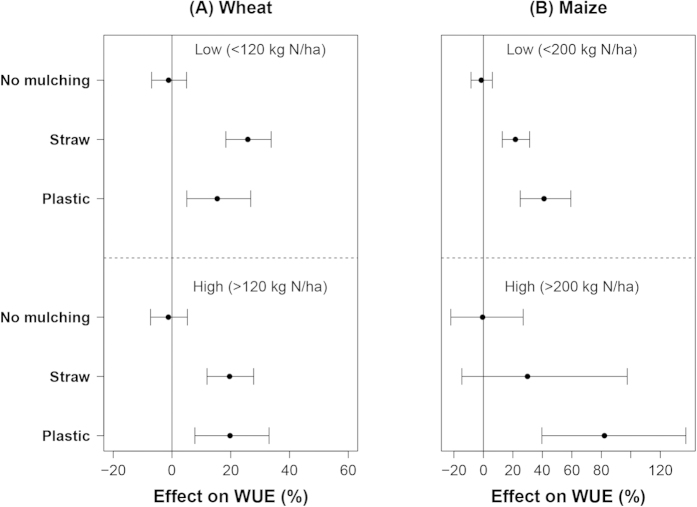
The effect of mulching on WUE of wheat (**A**) and maize (**B**) at different N input levels. Data were sub-grouped according to the 0.5 quantile value of the water input of each crop with plastic mulching treatments and displayed as low and high (from top to the bottom).

The mean effect of straw mulching on WUE of wheat was slightly larger at low N input (<120 kg N ha^−1^). In contrast, the mean effect of plastic mulching on WUE of wheat was slightly larger at the high N input level, but the differences between low and high N input were rather small (Fig. 6A). For maize, the mean effect of plastic mulching on WUE was 81% at high N input and 30% at low N input (Fig. 6B).

**Figure 7:**
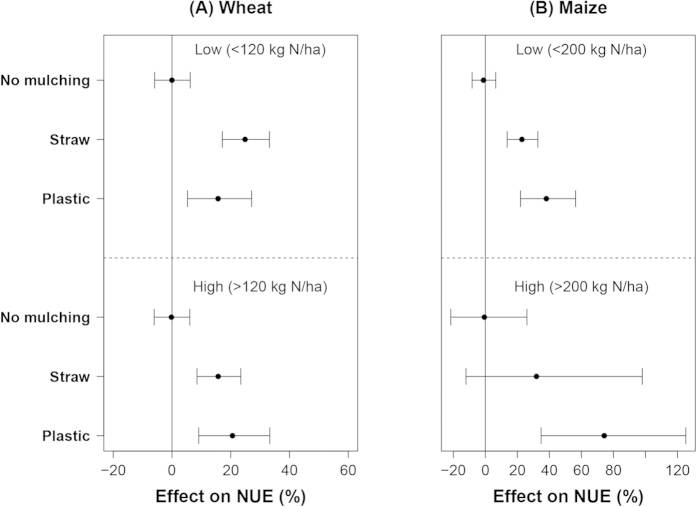
The effect of mulching on NUE of wheat (**A**) and maize (**B**) at different N input levels. Data were sub-grouped according to the 0.5 quantile value of the water input of each crop with plastic mulching treatments and displayed as low and high (from top to the bottom).

The mean effects of mulching (~20%) on NUE of wheat did not differ much between low and high N input (Fig. 7A). For maize, the mean effect of plastic mulching on NUE was 78% at high N input level and 35% at low N input. The CIs of the mulching effects were larger at high N input level (Fig. 7B).

### Effects of mulching at different temperature

**Figure 8:**
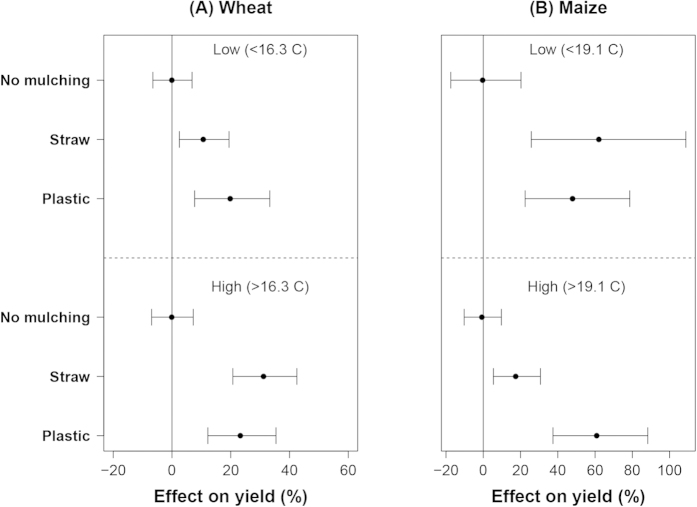
The effect of mulching on wheat (**A**) and maize (**B**) yields at different temperature. Data were sub-grouped according to the 0.5 quantile value of the seasonal mean temperature of each crop with plastic mulching treatments and displayed as low and high (from top to the bottom).

The effects of mulching were also affected by temperature. The mean seasonal temperature of the maize growing season was higher than that of wheat. Interestingly, at low temperature (4.9–16.3 °C), the effects of plastic mulching in wheat yields were larger than that of straw mulching; whereas the opposite trend was found at high temperature (16.3–25.5 °C) (Fig. 8A). In maize, the effect of straw mulching was 60% at low temperature (12.7–19.1 °C) and 18% at high temperature (19.1–30.4 °C). The mean effects of plastic mulching in maize yield were around 60% at both low and high temperature (Fig. 8B).

### Effects of mulching at different soil organic matter content

**Figure 9:**
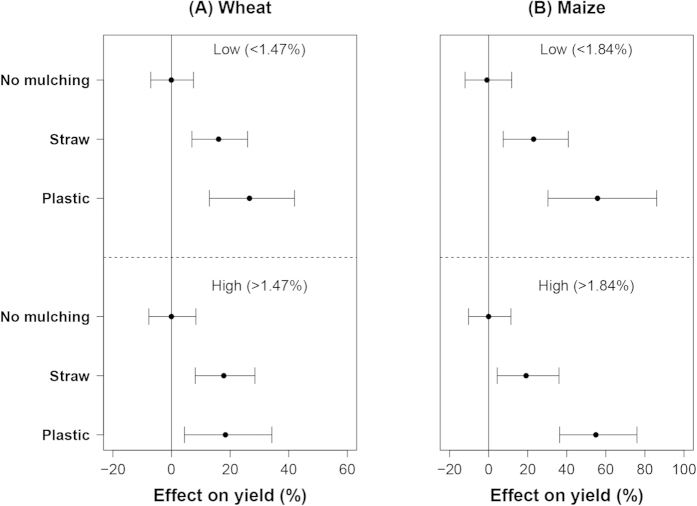
The effect of mulching on wheat (**A**) and maize (**B**) yields at different soil organic matter contents. Data were sub-grouped according to the 0.5 quantile value of the SOM of each crop with plastic mulching treatments and displayed as low and high (from top to the bottom).

The effects of mulching were not significantly affected by soil organic matter (SOM) content. The mean effect of both straw and plastic mulching in wheat yield was ~20% (Fig. 9A), irrespective of SOM content. For maize, the mean effect of straw and plastic mulching on yield was ~20 and 60%, respectively, again irrespective of SOM content (Fig. 9B). Soil organic matter content was mostly less than 2% in our dataset, indicating a relatively dry environment, where water availability and temperature may be dominant factors in determining crop yields. Furthermore, fertilization may also have ‘diluted’ the effects of SOM on mulching.

### Interactions between water and N inputs in yield, WUE and NUE

Results of the statistical analysis of the effects of mulching, water and N inputs and their interactions, as well as those of soil organic matter (SOM) and mean air temperature during the growing season, on yield, WUE and NUE are summarized in Tables 1, 2 and 3. Both straw mulching and plastic mulching had significant and positive effects on wheat and maize yields (Table 1). There were significant positive interactions between water and N inputs in wheat yield, indicating that the effect of N input increased with increased water input, and vice versa. However, the interactions between water and N inputs were not significant in maize yield, likely due to relatively high water availability for the maize growing season. Both wheat and maize yield significantly and positively responded to N input. Wheat yields were not significantly affected by SOM content and temperature; whereas maize yields were both positively related to SOM content and temperature.

WUE of wheat was negatively related to water input and positively to N input (Table 2). There were positive interactions between water and N inputs in WUE of wheat. WUE of maize was also negatively related to water (but not significantly) and positively to N inputs, but there was a negative interaction between water and N inputs, indicating that increases in water input reduced the positive effect of N on WUE. WUE of wheat and maize was not significantly related to SOM content and temperature.

NUE of wheat and maize were positively related to water input and negatively to N input (Table 3). There were negative interaction between water and N inputs in NUE of wheat and maize; however the interactive effects were not significant. The NUE of wheat and maize was significantly and positively related to SOM.

## Discussion

Our study provides a systematic and quantitative analysis on the effects of two common soil mulching techniques (i.e., straw mulching and plastic cover) on yields, WUE and NUE of wheat and maize, based on published experimental studies across the world. We focused on wheat and maize because of their importance in the global food production and food security, in total these two crops accounts for 70% of the world cereal production^1^. We excluded rice because rice is mainly grown as paddy rice with flood irrigation, and mulching on rice has not been extensively studied and reported. Results of our study indicate that mulching effects depend on crop type, water and N input levels and mean air temperature during the growing season.

First, we found that the effects of soil mulching (for both straw and plastic) were larger in maize than in wheat, which could be related to the larger yield potential of maize. Maize as a C4 crop is more efficient in photosynthesis than wheat as a C3 crop^28^. In our dataset, the highest maize yield was 15 ton ha^−1^ and the highest wheat yield was only 8 ton ha^−1^. Furthermore, maize has a much lower planting density (1.5 to 18 plants m^−2^) than wheat (135 to 540 plants m^−2^)^29^,^30^. Maize often grows during the summer period when temperature and evaporation are high, whereas wheat mainly grows during winter and early summer when the temperature and evaporation are relatively low. Moreover, there is often more rainwater in the maize growing season than in the wheat growing season. As a consequence, mulching may reduce evaporation more in maize than in wheat. In North China Plain, for example, evaporation during the full crop season averaged 28% of total evapotranspiration for winter wheat and 40% of that for summer maize^31^.

Second, the effects of soil mulching were affected by water and N input levels (Figs 2, 3, 4, 5, 6, 7). For maize, it seems that the effects of plastic cover tended to decrease with increased water input, likely because of smaller yield differences between mulched and non-mulched treatments at the near-optimal range of water input. However, for wheat, the effects of plastic cover were smaller at low water input (<250 mm) than at high water input, likely because the wheat yields of both mulched and non-mulched treatments were low under such severe water-limited situations. Input levels of N did not significantly influence the effect of soil mulching on wheat, but strongly increased that on maize. These trends were derived from an analysis of just two sub-datasets, split according to water and N input levels, the statistical power would have been low if the dataset were split into more sub-datasets.

The effects of soil mulching likely result from soil temperature modification and evaporation reduction. Straw mulching often reduces soil temperature whereas plastic cover increases soil temperature. For example, in North China Plain (a temperate region), decreased soil temperature by straw mulching may delay the germination and development of winter wheat, which may reduce grain yield by 5–7%^20^. Plastic mulching increases soil temperature, which may favour early seed generation and root growth^14^,^15^. This may explain why plastic mulching performs better for wheat at relatively low temperature. However, in tropical regions, straw mulching may modulate soil temperature in such a way that crop yield increases^32^,^33^. This contrasting pattern was shown in Fig. 8A. For maize, the effects of plastic mulching were significantly larger than that of straw mulching at high temperature (Fig. 8B), which is likely related to a more effective reduction in soil evaporation by plastic mulching compared to straw mulching. Furthermore, the positive effect of SOM in maize yields likely reflects an increased availability of N and/or other nutrients through the mineralization of SOM, especially at high temperature, because SOM is often positively related to N mineralization rate and also soil water holding capacity^34^,^35^,^36^,^37^,^38^. Wheat often grows at relative low temperature and has a longer growing season than maize. As a result, SOM may have less influence in wheat yields (Fig. 9).

Our results suggest that soil mulching may contribute to closing yield gaps between attainable and actual yields, and to increasing water and N use efficiencies. Mueller, *et al.*^2^ estimated that actual grain yields are often only 30 to 80% of the attainable yield, i.e., the yield obtained in well-managed field trials. These yield gaps may be narrowed down by improved water and nutrient management, which may include reducing the evaporative demand through mulching as indicated by this study. To be able to cash the full benefits of mulching, extension services, the plastic supplying industries and farmers must know the relationships between mulching effects, crop type and environmental conditions. Recently, progress has been made in the optimization of the time and methods (when and how) of mulching. For example, Bu, *et al.*^39^ concluded that plastic film mulching was more effective than straw and/or gravel mulching in counteracting water limitations and low temperatures in the Loess Plateau in China. Liu, *et al.*^40^ reported that removing the film at the silking stage decreased the plant senescence rate and slightly increased the final kernel number and weight, thus increasing the grain yield of maize by 0.6 to 1.2 ton ha^−1^. These results clearly indicate the importance of site-specific knowledge and management of mulching.

Thanks to governmental subsidies, plastic covers have been widely adopted, especially in China and India. For example, the Comprehensive Subsidy on Agricultural Inputs in China has increased from ¥12 billion in 2006 to ¥71.6 billion in 2010. As a result, the area of plastic film coverage reached ~20 million ha, and the amount of plastic film used reached 1.25 million tons in 2011^41^. However, plastic films have also negative effects. For example, production of plastic films is energy demanding, and residues of plastic cover may contribute to soil and environmental pollution; plastic residues have accumulated in soils and ditches in some regions. The practice of plastic covers may not be sustainable without proper collection and recycling of the residues of these plastic films. Alternatively, plastic mulching could be made from biodegradable plastic^21^.

Plastic mulching is also being tested in some regions in Africa. The potentials are relatively large, as the effects of covers on yield, WUE and NUE can be large in low water and N input areas. The question is whether plastic films have a future without subsidies, without site-specific and crop-specific guidelines, and without a proper mechanism for the collection and recycling of the residues. Use of plastic films in Africa is limited by the financial cost (and/or lack of governmental subsidies), but also by the cost of the distribution of the plastic films and the collection and recycling of the plastic residues. Straw mulching is limited by the availability of straw in the field, which is often being used also for feeding ruminants or as biofuel. This is often the case for small household farmers in Africa^42^. Alternative sources of mulches may be provided by pruning from agroforestry trees^43^,^44^, which require extra labour costs.

Evidently, straw and plastic mulching is not without difficulties. Governmental subsidies and extension services have stimulated its use in practice, and the results of our study shows that these mulching practices can have significant yields advantages. However, labour costs are relatively high, straw mulches are not always available and residues of plastic mulching are not easily collected and recycled. These side-effects as well as the site-specificity of the mulching effects must be included in the guidelines for mulching practices.

## Conclusions

Soil mulching can significantly increase yields (as well as WUE and NUE) of wheat and maize by 20% and 60%, respectively. Mean effects were larger for plastic films than for straw mulching. The effects of soil mulching depended on water and N input levels, temperature and to some extent also SOM. The effects tended to decrease with an increase in the availability of water, and tended to increase with N input. Significant interactions between water and N inputs in yields, WUE and NUE suggest that mulching effects strongly depend on environmental conditions. Soil mulching may contribute to closing the yield gap between attainable and actual yields.

Though soil mulching has clear positive and rather consistent effects on yields, WUE and NUE of wheat and maize, there are also clear trade-offs. Straw mulching is limited by the availability of straw in the field, which is often being used also for feeding ruminants or as biofuel. Use of plastic films is limited by the financial cost, but also by the cost of the collection and recycling of the plastic residues. Therefore, guidelines for mulching practices should consider the effects of water and N input levels, crop type and the side-effects of mulching.

## Additional Information

**How to cite this article**: Qin, W. *et al.* Soil mulching significantly enhances yields and water and nitrogen use efficiencies of maize and wheat: a meta-analysis. *Sci. Rep.* **5**, 16210; doi: 10.1038/srep16210 (2015).

## Supplementary Material

Supplementary Information

## Figures and Tables

**Table 1:** The effects of multiple variables on wheat and maize yields.

Crop	Item†	Estimate	SD	DF	*t* value	*p* value	Sign‡
Wheat	α (Intercept)	1.71	0.81	513.00	2.11	0.035	*
	β_1_ (Plastic)	0.56	0.15	49.00	3.73	0.001	**
	β_1_ (Straw)	0.46	0.10	49.00	4.61	0.000	***
	β_2_ (SOM)	0.03	0.19	513.00	0.18	0.857	NS
	β_3_ (Temperature)	0.01	0.04	513.00	0.12	0.905	NS
	β_4_ (Water)	5E-04	0.00	513.00	0.77	0.445	NS
	β_5_ (N)	6E-03	0.00	513.00	3.10	0.002	**
	β_6_ (W*N)	2E-05	0.00	513.00	4.27	0.000	***
Maize	α (Intercept)	−10.45	3.40	725.00	−3.07	0.002	**
	β_1_ (Plastic)	2.19	0.25	44.00	8.62	0.000	***
	β_1_ (Straw)	0.80	0.19	44.00	4.09	0.000	***
	β_2_ (SOM)	2.13	0.65	37.00	3.28	0.002	**
	β_3_ (Temperature)	0.47	0.12	725.00	3.77	0.000	***
	β_4_ (Water)	9E-04	0.00	725.00	1.14	0.256	NS
	β_5_ (N)	9E-03	0.00	725.00	3.51	0.001	**
	β_6_ (W*N)	−1E-06	0.00	725.00	−0.23	0.816	NS

^†^See formula (5) for explanation of the items.

^‡^“***” means p < 0.001, “**” means 0.001 < p < 0.01, “*” means 0.01 < p < 0.05 and “NS” means p > 0.05.

**Table 2:** The effects of multiple variables on WUE of wheat and maize.

Crop	Item†	Estimate	SD	DF	*t* value	*p* value	Sign‡
Wheat	α (Intercept)	1.01	0.24	513.00	4.23	0.000	***
	β_1_ (Plastic)	0.15	0.05	49.00	3.16	0.003	**
	β_1_ (Straw)	0.15	0.03	49.00	4.66	0.000	***
	β_2_ (SOM)	−0.01	0.06	513.00	−0.25	0.806	NS
	β_3_ (Temperature)	−5E-03	0.01	513.00	−0.37	0.715	NS
	β_4_ (Water)	−1E-03	0.00	513.00	−4.63	0.000	***
	β_5_ (N)	2E-03	0.00	513.00	3.62	0.000	***
	β_6_ (W*N)	4E-06	0.00	513.00	2.06	0.040	*
Maize	α (Intercept)	−0.85	0.82	725.00	−1.03	0.302	NS
	β_1_ (Plastic)	0.61	0.06	44.00	9.86	0.000	***
	β_1_ (Straw)	0.19	0.05	44.00	4.07	0.000	***
	β_2_ (SOM)	0.31	0.16	37.00	1.97	0.056	NS
	β_3_ (Temperature)	0.06	0.03	725.00	1.94	0.053	NS
	β_4_ (Water)	−8E-05	0.00	725.00	−0.40	0.693	NS
	β_5_ (N)	4E-03	0.00	725.00	6.36	0.000	***
	β_6_ (W*N)	−4E-06	0.00	725.00	−3.96	0.000	***

^†^See formula (5) for explanation of the items.

^‡^“***” means p < 0.001, “**” means 0.001 < p < 0.01, “*” means 0.01 < p < 0.05 and “NS” means p > 0.05.

**Table 3:** The effects of multiple variables on NUE of wheat and maize.

Crop	Item†	Estimate	SD	DF	*t* value	*p* value	Sign‡
Wheat	α (Intercept)	59.41	10.51	470.00	5.65	0.000	**
	β_1_ (Plastic)	5.53	1.72	49.00	3.22	0.002	**
	β_1_ (Straw)	5.47	1.09	49.00	5.01	0.000	***
	β_2_ (SOM)	7.99	2.44	470.00	3.27	0.001	**
	β_3_ (Temperature)	−8E-01	0.53	470.00	−1.60	0.110	NS
	β_4_ (Water)	5E-02	0.01	470.00	4.55	0.000	***
	β_5_ (N)	−3E-01	0.03	470.00	−9.40	0.000	***
	β_6_ (W*N)	−1E-04	0.00	470.00	−1.34	0.182	NS
Maize	α (Intercept)	−1.18	25.97	654.00	−0.05	0.964	NS
	β_1_ (Plastic)	18.35	3.30	43.00	5.55	0.000	***
	β_1_ (Straw)	6.20	2.67	43.00	2.32	0.025	*
	β_2_ (SOM)	10.22	4.43	36.00	2.31	0.027	*
	β_3_ (Temperature)	1.59	0.97	654.00	1.64	0.102	NS
	β_4_ (Water)	0.01	0.01	654.00	1.53	0.128	NS
	β_5_ (N)	−1E-01	0.03	654.00	−3.59	0.000	***
	β_6_ (W*N)	−5E-05	0.00	654.00	−1.01	0.312	NS

^†^See formula (5) for explanation of the items.

^‡^“***” means p < 0.001, “**” means 0.001 < p < 0.01, “*” means 0.01 < p < 0.05 and “NS” means p > 0.05.
