# Supplementary material for: Soil mulching significantly enhances yields and water and nitrogen use efficiencies of maize and wheat: a meta-analysis
Source: Sci Rep. 2015 Nov 20;5:16210. doi: 10.1038/srep16210 (PMC4653642; doi:10.1038/srep16210)
Supplement: Supplementary Information [file srep16210-s1.pdf]

Soil mulching significantly enhances yields and water and nitrogen use efficiencies of maize and wheat: a meta-analysis

Wei Qin [1, 2*], Chunsheng Hu[1*], Oene Oenema[1, 2, 3]

[1]*Centre for Agricultural Resources Research, Institute of Genetics and Developmental Biology, Chinese Academy of Sciences, Shijiazhuang, Hebei, China;*

[2]*Department of Soil Quality, Wageningen UR, 6700 AA, Wageningen, the Netherlands;*

[3]*Alterra, Wageningen UR, PO Box 47, 6700 AA, Wageningen, the Netherlands*

* Corresponding author. Tel.: +31 317 482975; fax: +31 317 419000.

E-mail address: wei.qin@wur.nl or weiqinwur@gmail.com (W. Qin) and cshu@sjziam.ac.cn (C. Hu)

Supplementary information- Figures and tables

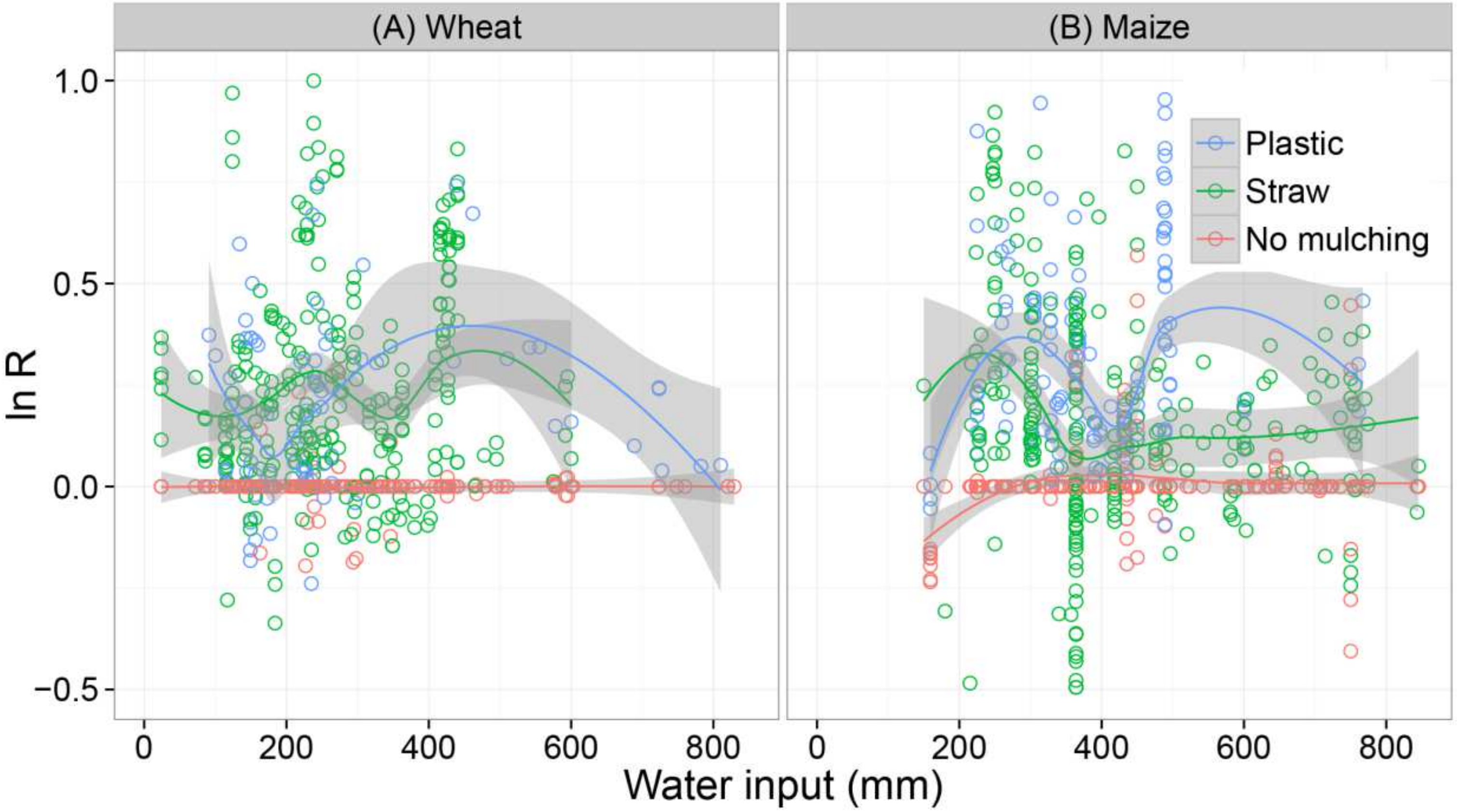

Supplementary Figure S1. Change of effect size (of yield) in different water input of wheat (A) and maize (B). Lines show the means fitted by local regression, gray areas show the 95% confidence interval.

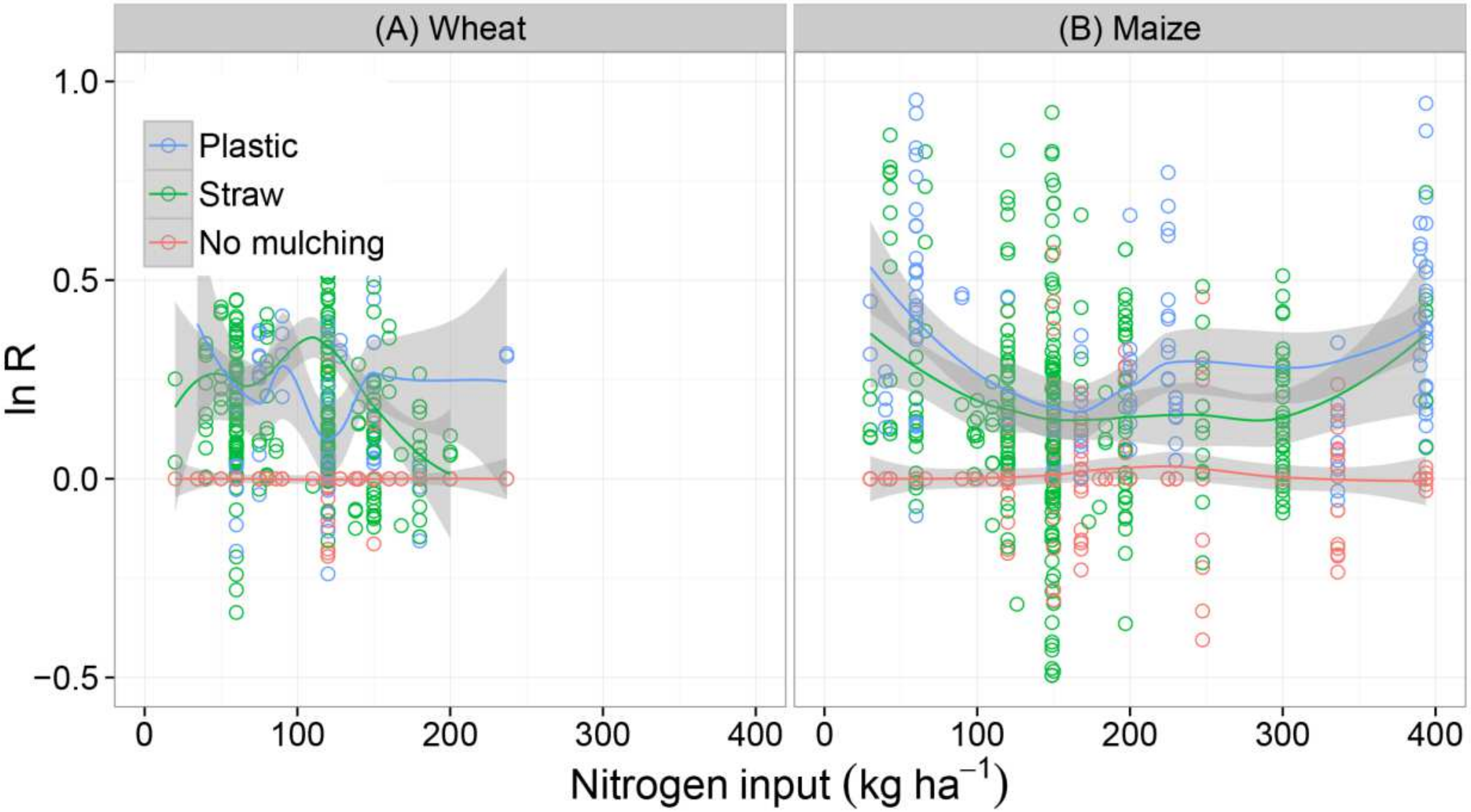

Supplementary Figure S2. Change of effect size (of yield) in different nitrogen input levels for wheat (A) and maize (B). Lines show the means fitted by local regression, gray areas show the 95% confidence interval.

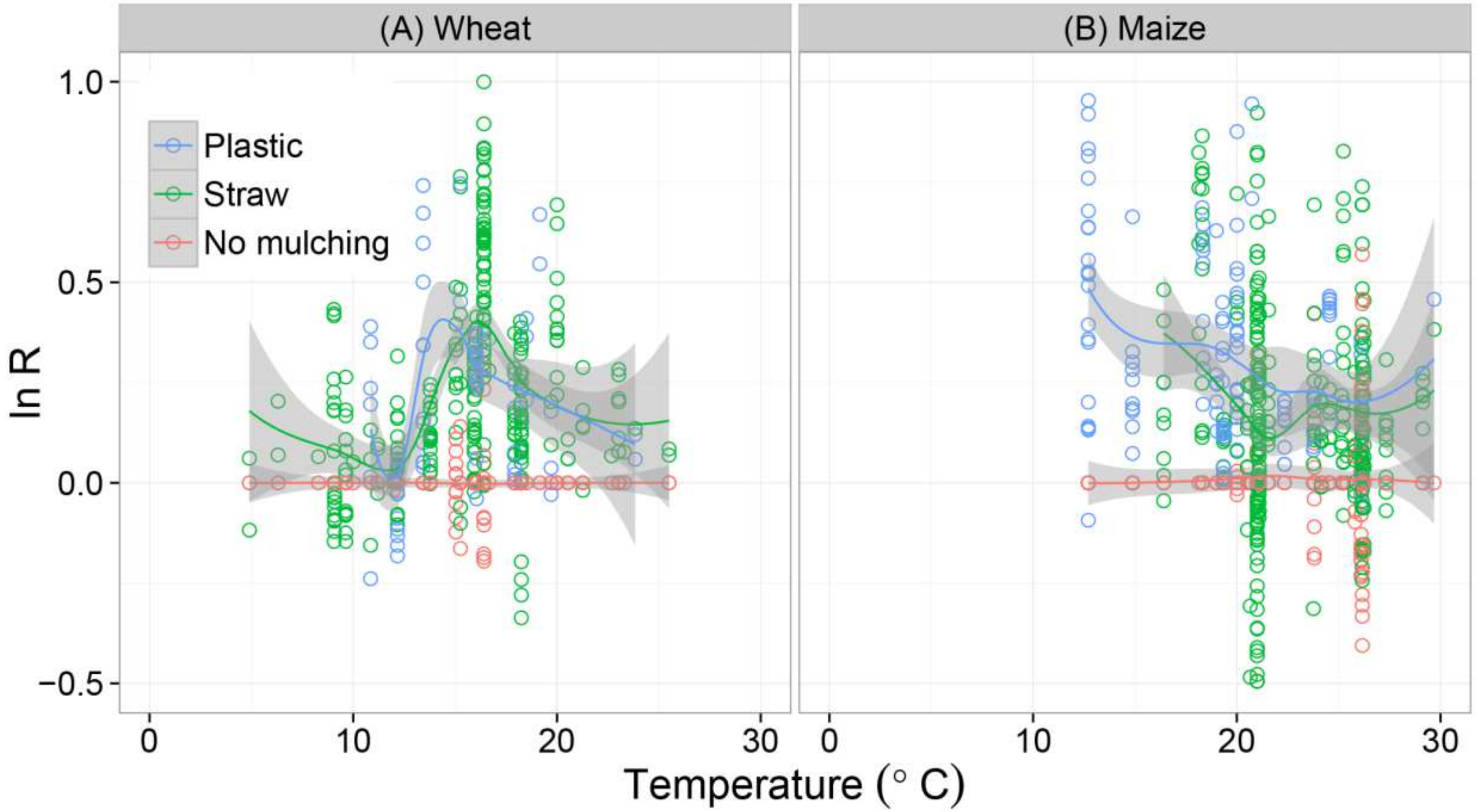

Supplementary Figure S3. Change of effect size (of yield) in different seasonal mean temperature of wheat (A) and maize (B). Lines show the means fitted by local regression, gray areas show the 95% confidence interval.

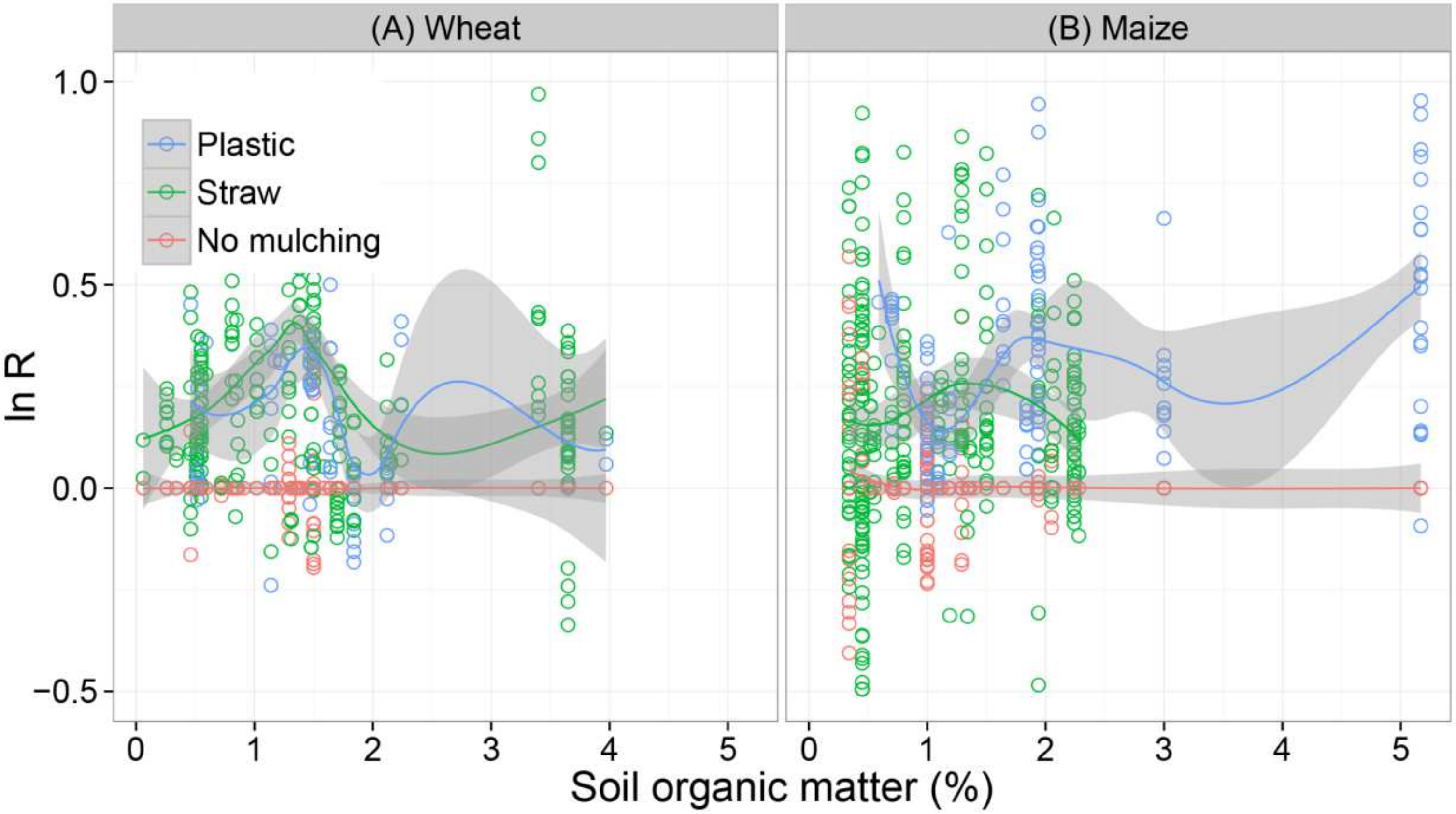

Supplementary Figure S4. Change of effect size (of yield) in different nitrogen input levels for wheat (A) and maize (B). Lines show the means fitted by local regression, gray areas show the 95% confidence interval.

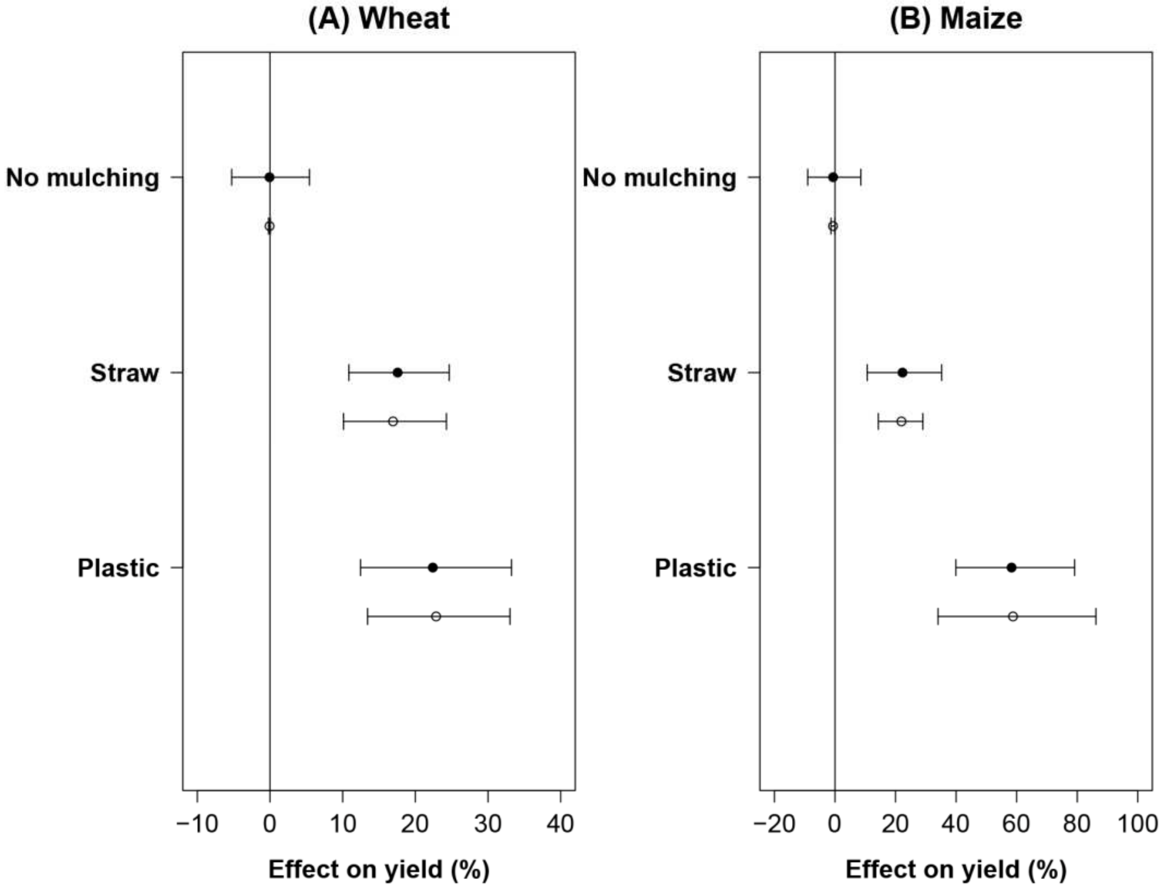

Supplementary Figure S5. Comparisons of the estimated mulching effects between original dataset and bootstrapping dataset (1000 iterations) of wheat (A) and maize (B). Solid black dots show the mean effects of original dataset, the open circle dots show the mean effect of bootstrapping dataset. error bars represent 95% confidence intervals.

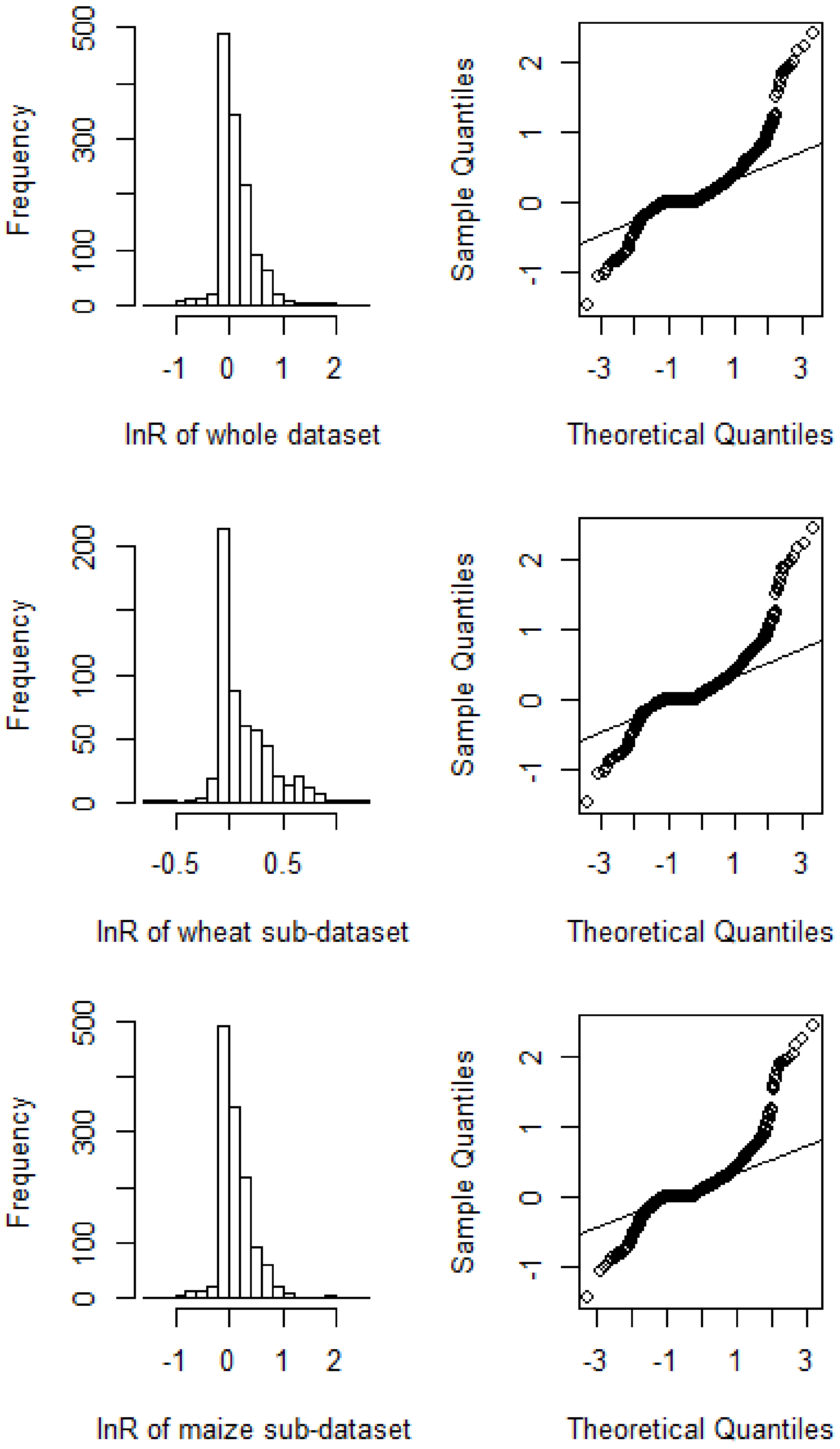

Supplementary Figure S6. Histogram of lnR of whole dataset and sub dataset of wheat and maize.

Supplementary Table S1. The dataset of the selected studies used in the meta-analysis.

Reference	Year	Location	Soil	Crop	Treatment	Yield	Rain	Irrigation	Total	ET	WUE	N	NUE	Temp	SOM
						ton ha^{-1}	mm	mm	mm	mm	kg m^{-3}	kg ha^{-1}	kg kg^{-1}	ºC	%
(Triplett et al., 1968)	1962	Ohio,USA	Silt loam	Maize	CK	4.04	217	0	217	307.22	1.32	168	24.05	21.56	2.07
(Triplett et al., 1968)	1962	Ohio,USA	Silt loam	Maize	CK	4.15	217	0	217	302.78	1.37	168	24.7	21.56	2.07
(Triplett et al., 1968)	1962	Ohio,USA	Silt loam	Maize	S	4.83	217	0	217	259.67	1.86	168	28.75	21.56	2.07
(Triplett et al., 1968)	1962	Ohio,USA	Silt loam	Maize	S	5.61	217	0	217	235.67	2.38	168	33.39	21.56	2.07
(Triplett et al., 1968)	1963	Ohio,USA	Silt loam	Maize	CK	5.65	261	0	261	385	1.47	168	33.63	21.56	2.07
(Triplett et al., 1968)	1963	Ohio,USA	Silt loam	Maize	CK	5.16	261	0	261	379.67	1.36	168	30.71	21.56	2.07
(Triplett et al., 1968)	1963	Ohio,USA	Silt loam	Maize	S	5.83	261	0	261	337.89	1.73	168	34.7	21.56	2.07
(Triplett et al., 1968)	1963	Ohio,USA	Silt loam	Maize	S	6.51	261	0	261	302.78	2.15	168	38.75	21.56	2.07
(Triplett et al., 1968)	1964	Ohio,USA	Silt loam	Maize	CK	6.29	396	0	396	452.44	1.39	168	37.44	21.56	2.07
(Triplett et al., 1968)	1964	Ohio,USA	Silt loam	Maize	CK	4.38	396	0	396	448.89	0.98	168	26.07	21.56	2.07
(Triplett et al., 1968)	1964	Ohio,USA	Silt loam	Maize	S	8.51	396	0	396	404	2.11	168	50.65	21.56	2.07
(Triplett et al., 1968)	1964	Ohio,USA	Silt loam	Maize	S	6.74	396	0	396	392.44	1.72	168	40.12	21.56	2.07
(Doss et al., 1970)	1963	Alabama, USA	Sandy loam	Maize	CK	5.76	435.4	0	435.4	366.5	1.57	168	34.26	26.1	1
(Doss et al., 1970)	1963	Alabama, USA	Sandy loam	Maize	CK	5.17	435.4	0	435.4	366.5	1.41	168	30.79	26.1	1
(Doss et al., 1970)	1963	Alabama, USA	Sandy loam	Maize	CK	5.7	435.4	0	435.4	366.5	1.56	168	33.95	26.1	1
(Doss et al., 1970)	1963	Alabama, USA	Sandy loam	Maize	CK	6.03	435.4	0	435.4	366.5	1.65	168	35.92	26.1	1
(Doss et al., 1970)	1963	Alabama, USA	Sandy loam	Maize	CK	5.01	435.4	0	435.4	366.5	1.37	336	14.92	26.1	1
(Doss et al., 1970)	1963	Alabama, USA	Sandy loam	Maize	CK	5.6	435.4	0	435.4	366.5	1.53	336	16.68	26.1	1
(Doss et al., 1970)	1963	Alabama, USA	Sandy loam	Maize	CK	6.51	435.4	0	435.4	366.5	1.78	336	19.38	26.1	1
(Doss et al., 1970)	1963	Alabama, USA	Sandy loam	Maize	CK	5.6	435.4	0	435.4	366.5	1.53	336	16.65	26.1	1
(Doss et al., 1970)	1963	Alabama, USA	Sandy loam	Maize	P	6.45	435.4	0	435.4	344.2	1.87	168	38.4	26.1	1
(Doss et al., 1970)	1963	Alabama, USA	Sandy loam	Maize	P	6.54	435.4	0	435.4	344.2	1.9	168	38.95	26.1	1
(Doss et al., 1970)	1963	Alabama, USA	Sandy loam	Maize	P	6.35	435.4	0	435.4	344.2	1.85	336	18.91	26.1	1

(Doss et al., 1970)	1963	Alabama, USA	Sandy loam	Maize	P	6.21	435.4	0	435.4	344.2	1.8	336	18.48	26.1	1
(Doss et al., 1970)	1963	Alabama, USA	Sandy loam	Maize	CK	5.88	435.4	209.6	645	506.2	1.16	168	35.01	26.1	1
(Doss et al., 1970)	1963	Alabama, USA	Sandy loam	Maize	CK	6.3	435.4	209.6	645	506.2	1.25	168	37.53	26.1	1
(Doss et al., 1970)	1963	Alabama, USA	Sandy loam	Maize	CK	6.17	435.4	209.6	645	506.2	1.22	168	36.75	26.1	1
(Doss et al., 1970)	1963	Alabama, USA	Sandy loam	Maize	CK	6.01	435.4	209.6	645	506.2	1.19	168	35.78	26.1	1
(Doss et al., 1970)	1963	Alabama, USA	Sandy loam	Maize	CK	6.1	435.4	209.6	645	506.2	1.21	336	18.16	26.1	1
(Doss et al., 1970)	1963	Alabama, USA	Sandy loam	Maize	CK	6.25	435.4	209.6	645	506.2	1.23	336	18.6	26.1	1
(Doss et al., 1970)	1963	Alabama, USA	Sandy loam	Maize	CK	6.9	435.4	209.6	645	506.2	1.36	336	20.53	26.1	1
(Doss et al., 1970)	1963	Alabama, USA	Sandy loam	Maize	CK	6.54	435.4	209.6	645	506.2	1.29	336	19.48	26.1	1
(Doss et al., 1970)	1963	Alabama, USA	Sandy loam	Maize	P	7.14	435.4	165.2	600.6	377.7	1.89	168	42.53	26.1	1
(Doss et al., 1970)	1963	Alabama, USA	Sandy loam	Maize	P	7.27	435.4	165.2	600.6	377.7	1.93	168	43.28	26.1	1
(Doss et al., 1970)	1963	Alabama, USA	Sandy loam	Maize	P	7.32	435.4	165.2	600.6	377.7	1.94	336	21.78	26.1	1
(Doss et al., 1970)	1963	Alabama, USA	Sandy loam	Maize	P	6.64	435.4	165.2	600.6	377.7	1.76	336	19.77	26.1	1
(Doss et al., 1970)	1964	Alabama, USA	Sandy loam	Maize	CK	3.72	159.3	0	159.3	228.6	1.63	168	22.14	26.1	1
(Doss et al., 1970)	1964	Alabama, USA	Sandy loam	Maize	CK	3.98	159.3	0	159.3	228.6	1.74	168	23.71	26.1	1
(Doss et al., 1970)	1964	Alabama, USA	Sandy loam	Maize	CK	4.01	159.3	0	159.3	228.6	1.76	168	23.88	26.1	1
(Doss et al., 1970)	1964	Alabama, USA	Sandy loam	Maize	CK	3.92	159.3	0	159.3	228.6	1.71	168	23.32	26.1	1
(Doss et al., 1970)	1964	Alabama, USA	Sandy loam	Maize	CK	4.13	159.3	0	159.3	228.6	1.8	336	12.28	26.1	1
(Doss et al., 1970)	1964	Alabama, USA	Sandy loam	Maize	CK	4.05	159.3	0	159.3	228.6	1.77	336	12.05	26.1	1
(Doss et al., 1970)	1964	Alabama, USA	Sandy loam	Maize	CK	4.17	159.3	0	159.3	228.6	1.83	336	12.43	26.1	1
(Doss et al., 1970)	1964	Alabama, USA	Sandy loam	Maize	CK	3.89	159.3	0	159.3	228.6	1.7	336	11.58	26.1	1
(Doss et al., 1970)	1964	Alabama, USA	Sandy loam	Maize	P	4.7	159.3	0	159.3	210.3	2.24	168	27.99	26.1	1
(Doss et al., 1970)	1964	Alabama, USA	Sandy loam	Maize	P	5.08	159.3	0	159.3	210.3	2.42	168	30.26	26.1	1
(Doss et al., 1970)	1964	Alabama, USA	Sandy loam	Maize	P	4.77	159.3	0	159.3	210.3	2.27	336	14.2	26.1	1
(Doss et al., 1970)	1964	Alabama, USA	Sandy loam	Maize	P	4.66	159.3	0	159.3	210.3	2.21	336	13.86	26.1	1
(Doss et al., 1970)	1964	Alabama, USA	Sandy loam	Maize	CK	5.46	159.3	273	432.3	406.7	1.34	168	32.49	26.1	1

(Doss et al., 1970)	1964	Alabama, USA	Sandy loam	Maize	CK	5.16	159.3	273	432.3	406.7	1.27	168	30.71	26.1	1
(Doss et al., 1970)	1964	Alabama, USA	Sandy loam	Maize	CK	5.38	159.3	273	432.3	406.7	1.32	168	32.03	26.1	1
(Doss et al., 1970)	1964	Alabama, USA	Sandy loam	Maize	CK	5.8	159.3	273	432.3	406.7	1.43	168	34.52	26.1	1
(Doss et al., 1970)	1964	Alabama, USA	Sandy loam	Maize	CK	5.84	159.3	273	432.3	406.7	1.44	336	17.38	26.1	1
(Doss et al., 1970)	1964	Alabama, USA	Sandy loam	Maize	CK	5.8	159.3	273	432.3	406.7	1.43	336	17.26	26.1	1
(Doss et al., 1970)	1964	Alabama, USA	Sandy loam	Maize	CK	6.24	159.3	273	432.3	406.7	1.53	336	18.58	26.1	1
(Doss et al., 1970)	1964	Alabama, USA	Sandy loam	Maize	CK	5.24	159.3	273	432.3	406.7	1.29	336	15.61	26.1	1
(Doss et al., 1970)	1964	Alabama, USA	Sandy loam	Maize	P	6.44	159.3	208.3	367.6	288	2.24	168	38.33	26.1	1
(Doss et al., 1970)	1964	Alabama, USA	Sandy loam	Maize	P	6.71	159.3	208.3	367.6	288	2.33	168	39.94	26.1	1
(Doss et al., 1970)	1964	Alabama, USA	Sandy loam	Maize	P	6.93	159.3	208.3	367.6	288	2.41	336	20.63	26.1	1
(Doss et al., 1970)	1964	Alabama, USA	Sandy loam	Maize	P	5.69	159.3	208.3	367.6	288	1.97	336	16.92	26.1	1
(Sandhu et al., 1992)	1980	New Delhi, India	Sandy loam	Maize	CK	2.38	644	0	644	526.12	0.45	60	39.67	27.33	0.55
(Sandhu et al., 1992)	1980	New Delhi, India	Sandy loam	Maize	S	2.75	644	0	644	597	0.46	60	45.83	27.33	0.55
(Sandhu et al., 1992)	1981	New Delhi, India	Sandy loam	Maize	CK	1.41	261	0	261	209	0.67	60	23.42	27.33	0.55
(Sandhu et al., 1992)	1981	New Delhi, India	Sandy loam	Maize	S	1.53	261	0	261	218	0.7	60	25.48	27.33	0.55
(Sandhu et al., 1992)	1981	New Delhi, India	Sandy loam	Maize	S	1.59	261	0	261	275	0.58	60	26.42	27.33	0.55
(Sandhu et al., 1992)	1982	New Delhi, India	Sandy loam	Maize	CK	1.7	441	0	441	456	0.37	60	28.28	27.33	0.55
(Sandhu et al., 1992)	1982	New Delhi, India	Sandy loam	Maize	S	1.96	441	0	441	453	0.43	60	32.67	27.33	0.55
(Sandhu et al., 1992)	1983	New Delhi, India	Sandy loam	Maize	CK	3.88	581	0	581	406	0.96	60	64.72	27.33	0.55
(Sandhu et al., 1992)	1983	New Delhi, India	Sandy loam	Maize	S	3.79	581	0	581	394	0.96	60	63.23	27.33	0.55
(Sandhu et al., 1992)	1983	New Delhi, India	Sandy loam	Maize	S	3.62	581	0	581	374	0.97	60	60.35	27.33	0.55
(Sandhu et al., 1992)	1984	New Delhi, India	Sandy loam	Maize	CK	3.09	543	0	543	331	0.93	60	51.47	27.33	0.55
(Sandhu et al., 1992)	1984	New Delhi, India	Sandy loam	Maize	S	3.44	543	0	543	350	0.98	60	57.35	27.33	0.55
(Sandhu et al., 1992)	1984	New Delhi, India	Sandy loam	Maize	S	4.2	543	0	543	369	1.14	60	69.93	27.33	0.55
(Sandhu et al., 1992)	1985	New Delhi, India	Sandy loam	Maize	CK	3.49	774	0	774	638	0.55	60	58.08	27.33	0.55
(Sandhu et al., 1992)	1985	New Delhi, India	Sandy loam	Maize	S	3.54	774	0	774	631	0.56	60	59.03	27.33	0.55

(Sandhu et al., 1992)	1985	New Delhi, India	Sandy loam	Maize	S	4.07	774	0	774	652	0.62	60	67.9	27.33	0.55
(Acharya et al., 1994)	1980	Himachal Pradesh, India	Silty clay loam	Maize	CK	5.95	1904	0	1904	623.7	0.95	120	49.57	23.8	1.29
(Acharya et al., 1994)	1980	Himachal Pradesh, India	Silty clay loam	Maize	S	6.67	1904	0	1904	623.7	1.07	120	55.59	23.8	1.29
(Acharya et al., 1994)	1980	Himachal Pradesh, India	Silty clay loam	Maize	CK	5.94	1904	0	1904	623.7	0.95	120	49.52	23.8	1.29
(Acharya et al., 1994)	1980	Himachal Pradesh, India	Silty clay loam	Maize	CK	6.81	1904	0	1904	623.7	1.09	120	56.76	23.8	1.29
(Acharya et al., 1994)	1981	Himachal Pradesh, India	Silty clay loam	Maize	CK	4.85	1943	0	1943	680.8	0.71	120	40.42	23.8	1.29
(Acharya et al., 1994)	1981	Himachal Pradesh, India	Silty clay loam	Maize	S	5.14	1943	0	1943	680.8	0.75	120	42.83	23.8	1.29
(Acharya et al., 1994)	1981	Himachal Pradesh, India	Silty clay loam	Maize	CK	3.49	1943	0	1943	680.8	0.51	120	29.1	23.8	1.29
(Acharya et al., 1994)	1981	Himachal Pradesh, India	Silty clay loam	Maize	CK	4.82	1943	0	1943	680.8	0.71	120	40.2	23.8	1.29
(Acharya et al., 1994)	1982	Himachal Pradesh, India	Silty clay loam	Maize	CK	4.48	1270	0	1270	489.7	0.92	120	37.34	23.8	1.29
(Acharya et al., 1994)	1982	Himachal Pradesh, India	Silty clay loam	Maize	S	5.78	1270	0	1270	489.7	1.18	120	48.13	23.8	1.29
(Acharya et al., 1994)	1982	Himachal Pradesh, India	Silty clay loam	Maize	CK	4.14	1270	0	1270	489.7	0.85	120	34.49	23.8	1.29
(Acharya et al., 1994)	1982	Himachal Pradesh, India	Silty clay loam	Maize	S	4.86	1270	0	1270	489.7	0.99	120	40.53	23.8	1.29
(Acharya et al., 1994)	1982	Himachal Pradesh, India	Silty clay loam	Maize	CK	4.83	1270	0	1270	489.7	0.99	120	40.28	23.8	1.29
(Acharya et al., 1994)	1983	Himachal Pradesh, India	Silty clay loam	Maize	CK	5	1631	0	1631	576	0.87	120	41.69	23.8	1.29
(Acharya et al., 1994)	1983	Himachal Pradesh, India	Silty clay loam	Maize	S	5.84	1631	0	1631	576	1.01	120	48.68	23.8	1.29
(Acharya et al., 1994)	1983	Himachal Pradesh, India	Silty clay loam	Maize	CK	4.06	1631	0	1631	576	0.7	120	33.82	23.8	1.29
(Acharya et al., 1994)	1983	Himachal Pradesh, India	Silty clay loam	Maize	S	5.04	1631	0	1631	576	0.88	120	42.03	23.8	1.29
(Acharya et al., 1994)	1983	Himachal Pradesh, India	Silty clay loam	Maize	CK	5.55	1631	0	1631	576	0.96	120	46.26	23.8	1.29
(Acharya et al., 1994)	1984	Himachal Pradesh, India	Silty clay loam	Maize	CK	4.76	1851	0	1851	578.2	0.82	120	39.63	23.8	1.29
(Acharya et al., 1994)	1984	Himachal Pradesh, India	Silty clay loam	Maize	S	5.58	1851	0	1851	578.2	0.96	120	46.46	23.8	1.29
(Acharya et al., 1994)	1984	Himachal Pradesh, India	Silty clay loam	Maize	CK	3.37	1851	0	1851	578.2	0.58	120	28.06	23.8	1.29
(Acharya et al., 1994)	1984	Himachal Pradesh, India	Silty clay loam	Maize	S	4.86	1851	0	1851	578.2	0.84	120	40.48	23.8	1.29
(Acharya et al., 1994)	1984	Himachal Pradesh, India	Silty clay loam	Maize	CK	5.25	1851	0	1851	578.2	0.91	120	43.77	23.8	1.29
(Acharya et al., 1994)	1985	Himachal Pradesh, India	Silty clay loam	Maize	CK	4.96	2003	0	2003	727.2	0.68	120	41.36	23.8	1.29
(Acharya et al., 1994)	1985	Himachal Pradesh, India	Silty clay loam	Maize	S	6.51	2003	0	2003	727.2	0.89	120	54.22	23.8	1.29

(Acharya et al., 1994)	1985	Himachal Pradesh, India	Silty clay loam	Maize	CK	1.55	2003	0	2003	727.2	0.21	120	12.9	23.8	1.29
(Acharya et al., 1994)	1985	Himachal Pradesh, India	Silty clay loam	Maize	S	4.97	2003	0	2003	727.2	0.68	120	41.44	23.8	1.29
(Acharya et al., 1994)	1985	Himachal Pradesh, India	Silty clay loam	Maize	CK	5.75	2003	0	2003	727.2	0.79	120	47.94	23.8	1.29
(Wicks et al., 1994)	1981	Wilber, Nebrask, USA	Silt loam	Maize	CK	4.95	600	0	600	600	0.83	100	49.5	21.53	1.5
(Wicks et al., 1994)	1981	Wilber, Nebrask, USA	Silt loam	Maize	S	5.52	600	0	600	600	0.92	100	55.2	21.53	1.5
(Wicks et al., 1994)	1981	Wilber, Nebrask, USA	Silt loam	Maize	S	5.44	600	0	600	600	0.91	100	54.4	21.53	1.5
(Wicks et al., 1994)	1981	Wilber, Nebrask, USA	Silt loam	Maize	S	5.77	600	0	600	600	0.96	100	57.7	21.53	1.5
(Wicks et al., 1994)	1981	Wilber, Nebrask, USA	Silt loam	Maize	S	6.03	600	0	600	600	1.01	100	60.3	21.53	1.5
(Wicks et al., 1994)	1981	NorthP latte	Silt loam	Maize	CK	5.43	300	0	300	300	1.81	98	55.36	19.39	1.5
(Wicks et al., 1994)	1981	NorthP latte	Silt loam	Maize	S	6.05	300	0	300	300	2.02	98	61.78	19.39	1.5
(Wicks et al., 1994)	1981	NorthP latte	Silt loam	Maize	S	6.27	300	0	300	300	2.09	98	63.94	19.39	1.5
(Wicks et al., 1994)	1981	NorthP latte	Silt loam	Maize	S	6.09	300	0	300	300	2.03	98	62.09	19.39	1.5
(Wicks et al., 1994)	1981	NorthP latte	Silt loam	Maize	S	5.49	300	0	300	300	1.83	98	55.99	19.39	1.5
(Wicks et al., 1994)	1982	Sidney	Silt loam	Maize	CK	1.51	306	0	306	306	0.49	66	22.85	18.14	1.5
(Wicks et al., 1994)	1982	Sidney	Silt loam	Maize	S	2.19	306	0	306	306	0.72	66	33.21	18.14	1.5
(Wicks et al., 1994)	1982	Sidney	Silt loam	Maize	S	2.74	306	0	306	306	0.9	66	41.59	18.14	1.5
(Wicks et al., 1994)	1982	Sidney	Silt loam	Maize	S	3.15	306	0	306	306	1.03	66	47.66	18.14	1.5
(Wicks et al., 1994)	1982	Sidney	Silt loam	Maize	S	3.44	306	0	306	306	1.13	66	52.18	18.14	1.5
(Lal et al., 1995)	1979	Ibadan, Nigeria	Luvisol	Maize	CK	0.64	585.8	0	585.8	585.8	0.11	120	5.33	25.23	0.8
(Lal et al., 1995)	1979	Ibadan, Nigeria	Luvisol	Maize	S	0.59	585.8	0	585.8	585.8	0.1	120	4.92	25.23	0.8
(Lal et al., 1995)	1979	Ibadan, Nigeria	Luvisol	Maize	CK	0.33	585.8	0	585.8	585.8	0.06	120	2.75	25.23	0.8
(Lal et al., 1995)	1979	Ibadan, Nigeria	Luvisol	Maize	S	0.39	585.8	0	585.8	585.8	0.07	120	3.25	25.23	0.8
(Lal et al., 1995)	1980	Ibadan, Nigeria	Luvisol	Maize	CK	0.85	845.4	0	845.4	845.4	0.1	120	7.08	25.23	0.8
(Lal et al., 1995)	1980	Ibadan, Nigeria	Luvisol	Maize	S	1.5	845.4	0	845.4	845.4	0.18	120	12.5	25.23	0.8
(Lal et al., 1995)	1980	Ibadan, Nigeria	Luvisol	Maize	CK	0.58	845.4	0	845.4	845.4	0.07	120	4.83	25.23	0.8
(Lal et al., 1995)	1980	Ibadan, Nigeria	Luvisol	Maize	S	0.61	845.4	0	845.4	845.4	0.07	120	5.08	25.23	0.8

(Lal et al., 1995)	1980	Ibadan, Nigeria	Luvisol	Maize	CK	1.42	604.3	0	604.3	604.3	0.23	120	11.83	26.25	0.8
(Lal et al., 1995)	1980	Ibadan, Nigeria	Luvisol	Maize	S	1.56	604.3	0	604.3	604.3	0.26	120	13	26.25	0.8
(Lal et al., 1995)	1980	Ibadan, Nigeria	Luvisol	Maize	CK	1	604.3	0	604.3	604.3	0.17	120	8.33	26.25	0.8
(Lal et al., 1995)	1980	Ibadan, Nigeria	Luvisol	Maize	S	1.24	604.3	0	604.3	604.3	0.21	120	10.33	26.25	0.8
(Lal et al., 1995)	1981	Ibadan, Nigeria	Luvisol	Maize	CK	0.21	432.4	0	432.4	432.4	0.05	120	1.75	25.23	0.8
(Lal et al., 1995)	1981	Ibadan, Nigeria	Luvisol	Maize	S	0.48	432.4	0	432.4	432.4	0.11	120	4	25.23	0.8
(Lal et al., 1995)	1981	Ibadan, Nigeria	Luvisol	Maize	CK	0.27	432.4	0	432.4	432.4	0.06	120	2.25	25.23	0.8
(Lal et al., 1995)	1981	Ibadan, Nigeria	Luvisol	Maize	S	0.33	432.4	0	432.4	432.4	0.08	120	2.75	25.23	0.8
(Lal et al., 1995)	1981	Ibadan, Nigeria	Luvisol	Maize	CK	3.25	636.8	0	636.8	636.8	0.51	120	27.08	26.25	0.8
(Lal et al., 1995)	1981	Ibadan, Nigeria	Luvisol	Maize	S	4.26	636.8	0	636.8	636.8	0.67	120	35.5	26.25	0.8
(Lal et al., 1995)	1981	Ibadan, Nigeria	Luvisol	Maize	CK	1.3	636.8	0	636.8	636.8	0.2	120	10.83	26.25	0.8
(Lal et al., 1995)	1981	Ibadan, Nigeria	Luvisol	Maize	S	1.84	636.8	0	636.8	636.8	0.29	120	15.33	26.25	0.8
(Lal et al., 1995)	1982	Ibadan, Nigeria	Luvisol	Maize	CK	0.41	223.6	0	223.6	223.6	0.18	120	3.42	25.23	0.8
(Lal et al., 1995)	1982	Ibadan, Nigeria	Luvisol	Maize	S	0.73	223.6	0	223.6	223.6	0.33	120	6.08	25.23	0.8
(Lal et al., 1995)	1982	Ibadan, Nigeria	Luvisol	Maize	CK	0.43	223.6	0	223.6	223.6	0.19	120	3.58	25.23	0.8
(Lal et al., 1995)	1982	Ibadan, Nigeria	Luvisol	Maize	S	0.6	223.6	0	223.6	223.6	0.27	120	5	25.23	0.8
(Lal et al., 1995)	1982	Ibadan, Nigeria	Luvisol	Maize	CK	3.3	615.2	0	615.2	615.2	0.54	120	27.5	26.25	0.8
(Lal et al., 1995)	1982	Ibadan, Nigeria	Luvisol	Maize	S	3.43	615.2	0	615.2	615.2	0.56	120	28.58	26.25	0.8
(Lal et al., 1995)	1982	Ibadan, Nigeria	Luvisol	Maize	CK	2.9	615.2	0	615.2	615.2	0.47	120	24.17	26.25	0.8
(Lal et al., 1995)	1982	Ibadan, Nigeria	Luvisol	Maize	S	3.02	615.2	0	615.2	615.2	0.49	120	25.17	26.25	0.8
(Lal et al., 1995)	1983	Ibadan, Nigeria	Luvisol	Maize	CK	0.64	230.6	0	230.6	230.6	0.28	120	5.33	25.23	0.8
(Lal et al., 1995)	1983	Ibadan, Nigeria	Luvisol	Maize	S	0.93	230.6	0	230.6	230.6	0.4	120	7.75	25.23	0.8
(Lal et al., 1995)	1983	Ibadan, Nigeria	Luvisol	Maize	CK	0.92	230.6	0	230.6	230.6	0.4	120	7.67	25.23	0.8
(Lal et al., 1995)	1983	Ibadan, Nigeria	Luvisol	Maize	S	0.97	230.6	0	230.6	230.6	0.42	120	8.08	25.23	0.8
(Lal et al., 1995)	1983	Ibadan, Nigeria	Luvisol	Maize	CK	2.85	580.9	0	580.9	580.9	0.49	120	23.75	26.25	0.8
(Lal et al., 1995)	1983	Ibadan, Nigeria	Luvisol	Maize	S	3.04	580.9	0	580.9	580.9	0.52	120	25.33	26.25	0.8

(Lal et al., 1995)	1983	Ibadan, Nigeria	Luvisol	Maize	CK	3.14	580.9	0	580.9	580.9	0.54	120	26.17	26.25	0.8
(Lal et al., 1995)	1983	Ibadan, Nigeria	Luvisol	Maize	S	2.95	580.9	0	580.9	580.9	0.51	120	24.58	26.25	0.8
(Lal et al., 1995)	1984	Ibadan, Nigeria	Luvisol	Maize	CK	1.98	480.6	0	480.6	480.6	0.41	120	16.5	25.23	0.8
(Lal et al., 1995)	1984	Ibadan, Nigeria	Luvisol	Maize	S	2.67	480.6	0	480.6	480.6	0.56	120	22.25	25.23	0.8
(Lal et al., 1995)	1984	Ibadan, Nigeria	Luvisol	Maize	CK	2.14	480.6	0	480.6	480.6	0.45	120	17.83	25.23	0.8
(Lal et al., 1995)	1984	Ibadan, Nigeria	Luvisol	Maize	S	2.46	480.6	0	480.6	480.6	0.51	120	20.5	25.23	0.8
(Lal et al., 1995)	1984	Ibadan, Nigeria	Luvisol	Maize	CK	4.47	681.5	0	681.5	681.5	0.66	120	37.25	26.25	0.8
(Lal et al., 1995)	1984	Ibadan, Nigeria	Luvisol	Maize	S	5.11	681.5	0	681.5	681.5	0.75	120	42.58	26.25	0.8
(Lal et al., 1995)	1984	Ibadan, Nigeria	Luvisol	Maize	CK	4.11	681.5	0	681.5	681.5	0.6	120	34.25	26.25	0.8
(Lal et al., 1995)	1984	Ibadan, Nigeria	Luvisol	Maize	S	4.29	681.5	0	681.5	681.5	0.63	120	35.75	26.25	0.8
(Lal et al., 1995)	1985	Ibadan, Nigeria	Luvisol	Maize	CK	0.55	735.5	0	735.5	735.5	0.07	120	4.58	25.23	0.8
(Lal et al., 1995)	1985	Ibadan, Nigeria	Luvisol	Maize	S	1.07	735.5	0	735.5	735.5	0.15	120	8.92	25.23	0.8
(Lal et al., 1995)	1985	Ibadan, Nigeria	Luvisol	Maize	CK	0.65	735.5	0	735.5	735.5	0.09	120	5.42	25.23	0.8
(Lal et al., 1995)	1985	Ibadan, Nigeria	Luvisol	Maize	S	0.77	735.5	0	735.5	735.5	0.1	120	6.42	25.23	0.8
(Lal et al., 1995)	1985	Ibadan, Nigeria	Luvisol	Maize	CK	2.17	935.7	0	935.7	935.7	0.23	120	18.08	26.25	0.8
(Lal et al., 1995)	1985	Ibadan, Nigeria	Luvisol	Maize	S	2.89	935.7	0	935.7	935.7	0.31	120	24.08	26.25	0.8
(Lal et al., 1995)	1985	Ibadan, Nigeria	Luvisol	Maize	CK	2.11	935.7	0	935.7	935.7	0.23	120	17.58	26.25	0.8
(Lal et al., 1995)	1985	Ibadan, Nigeria	Luvisol	Maize	S	1.81	935.7	0	935.7	935.7	0.19	120	15.08	26.25	0.8
(Lal et al., 1995)	1986	Ibadan, Nigeria	Luvisol	Maize	CK	0.32	379.2	0	379.2	379.2	0.08	120	2.67	25.23	0.8
(Lal et al., 1995)	1986	Ibadan, Nigeria	Luvisol	Maize	S	0.65	379.2	0	379.2	379.2	0.17	120	5.42	25.23	0.8
(Lal et al., 1995)	1986	Ibadan, Nigeria	Luvisol	Maize	CK	0.23	379.2	0	379.2	379.2	0.06	120	1.92	25.23	0.8
(Lal et al., 1995)	1986	Ibadan, Nigeria	Luvisol	Maize	S	0.23	379.2	0	379.2	379.2	0.06	120	1.92	25.23	0.8
(Lal et al., 1995)	1986	Ibadan, Nigeria	Luvisol	Maize	CK	1.41	714.2	0	714.2	714.2	0.2	120	11.75	26.25	0.8
(Lal et al., 1995)	1986	Ibadan, Nigeria	Luvisol	Maize	S	2.05	714.2	0	714.2	714.2	0.29	120	17.08	26.25	0.8
(Lal et al., 1995)	1986	Ibadan, Nigeria	Luvisol	Maize	CK	2.35	714.2	0	714.2	714.2	0.33	120	19.58	26.25	0.8
(Lal et al., 1995)	1986	Ibadan, Nigeria	Luvisol	Maize	S	1.98	714.2	0	714.2	714.2	0.28	120	16.5	26.25	0.8

(Lal et al., 1995)	1987	Ibadan, Nigeria	Luvisol	Maize	CK	0.73	723.5	0	723.5	723.5	0.1	120	6.08	26.25	0.8
(Lal et al., 1995)	1987	Ibadan, Nigeria	Luvisol	Maize	S	1.15	723.5	0	723.5	723.5	0.16	120	9.58	26.25	0.8
(Lal et al., 1995)	1987	Ibadan, Nigeria	Luvisol	Maize	CK	0.81	723.5	0	723.5	723.5	0.11	120	6.75	26.25	0.8
(Lal et al., 1995)	1987	Ibadan, Nigeria	Luvisol	Maize	S	1.05	723.5	0	723.5	723.5	0.15	120	8.75	26.25	0.8
(Li et al., 2000)	1998	Lanzhou, China	Sandy loam	Maize	P	8.9	304	0	314	314	2.84	393.75	22.61	20.74	1.94
(Li et al., 2000)	1998	Lanzhou, China	Sandy loam	Maize	P	7.03	304	0	329	329	2.14	393.75	17.84	20.74	1.94
(Li et al., 2000)	1998	Lanzhou, China	Sandy loam	Maize	CK	3.46	304	0	379	379	0.91	393.75	8.8	20.74	1.94
(Li et al., 2000)	1998	Lanzhou, China	Sandy loam	Maize	CK	4.78	304	0	379	379	1.26	393.75	12.15	20.74	1.94
(Sharma et al., 2000)	1994	Himachal Pradesh, India	Silty clay loam	Maize	S	1.53	1500	0	1500	700	0.22	150	10.2	16.41	1.5
(Sharma et al., 2000)	1994	Himachal Pradesh, India	Silty clay loam	Maize	S	1.63	1500	0	1500	700	0.23	150	10.87	16.41	1.5
(Sharma et al., 2000)	1994	Himachal Pradesh, India	Silty clay loam	Maize	S	1.7	1500	0	1500	700	0.24	150	11.33	16.41	1.5
(Sharma et al., 2000)	1994	Himachal Pradesh, India	Silty clay loam	Maize	CK	1.6	1500	0	1500	700	0.23	150	10.67	16.41	1.5
(Sharma et al., 2000)	1995	Himachal Pradesh, India	Silty clay loam	Maize	S	3.4	1500	0	1500	700	0.49	150	22.67	16.41	1.5
(Sharma et al., 2000)	1995	Himachal Pradesh, India	Silty clay loam	Maize	S	3.69	1500	0	1500	700	0.53	150	24.6	16.41	1.5
(Sharma et al., 2000)	1995	Himachal Pradesh, India	Silty clay loam	Maize	S	3.33	1500	0	1500	700	0.48	150	22.2	16.41	1.5
(Sharma et al., 2000)	1995	Himachal Pradesh, India	Silty clay loam	Maize	CK	2.87	1500	0	1500	700	0.41	150	19.13	16.41	1.5
(Sharma et al., 2000)	1996	Himachal Pradesh, India	Silty clay loam	Maize	S	4.12	1500	0	1500	700	0.59	150	27.47	16.41	1.5
(Sharma et al., 2000)	1996	Himachal Pradesh, India	Silty clay loam	Maize	S	4.45	1500	0	1500	700	0.64	150	29.67	16.41	1.5
(Sharma et al., 2000)	1996	Himachal Pradesh, India	Silty clay loam	Maize	CK	2.75	1500	0	1500	700	0.39	150	18.33	16.41	1.5
(Sharma et al., 2000)	1996	Himachal Pradesh, India	Silty clay loam	Maize	S	3.53	1500	0	1500	700	0.5	150	23.53	16.41	1.5
(Li et al., 2001)	1998	Lanzhou, China	Sandy loam	Maize	S	7.7	225	0	225	377.65	2.04	393.75	19.57	20.01	1.94
(Li et al., 2001)	1998	Lanzhou, China	Sandy loam	Maize	P	8.99	225	0	225	397.82	2.26	393.75	22.83	20.01	1.94
(Li et al., 2001)	1998	Lanzhou, China	Sandy loam	Maize	P	7.12	225	0	225	337.59	2.11	393.75	18.09	20.01	1.94
(Li et al., 2001)	1998	Lanzhou, China	Sandy loam	Maize	CK	3.8	225	0	225	351.41	1.08	393.75	9.64	20.01	1.94
(Li et al., 2001)	1998	Lanzhou, China	Sandy loam	Maize	CK	3.69	225	0	225	358.6	1.03	393.75	9.38	20.01	1.94
(Li et al., 2001)	1999	Lanzhou, China	Sandy loam	Maize	S	8.18	328	0	328	370.29	2.21	393.75	20.78	20.01	1.94

(Li et al., 2001)	1999	Lanzhou, China	Sandy loam	Maize	P	7.61	328	0	328	382.23	1.99	393.75	19.32	20.01	1.94
(Li et al., 2001)	1999	Lanzhou, China	Sandy loam	Maize	P	6.39	328	0	328	331.24	1.93	393.75	16.24	20.01	1.94
(Li et al., 2001)	1999	Lanzhou, China	Sandy loam	Maize	CK	5.59	328	0	328	390.64	1.43	393.75	14.19	20.01	1.94
(Li et al., 2001)	1999	Lanzhou, China	Sandy loam	Maize	CK	5.27	328	0	328	373.54	1.41	393.75	13.38	20.01	1.94
(Zhang et al., 2003)	1998	Luancheng, China	Loam	Maize	S	7.12	120	184	304	386	1.84	150	47.46	25.4	1.4
(Zhang et al., 2003)	1998	Luancheng, China	Loam	Maize	CK	6.66	120	184	304	431	1.55	150	44.39	25.4	1.4
(Zhang et al., 2004)	2000	Luancheng, China	Loam	Maize	CK	3.92	348.3	165.7	514	387.9	1.01	207	18.94	25.4	1.2
(Zhang et al., 2004)	2000	Luancheng, China	Loam	Maize	S	3.97	348.3	80	428.3	281.3	1.41	207	19.18	25.4	1.2
(Zhang et al., 2004)	2001	Luancheng, China	Loam	Maize	CK	6.17	211.9	107.3	319.2	358	1.72	207	29.81	25.4	1.2
(Zhang et al., 2004)	2001	Luancheng, China	Loam	Maize	S	3.53	211.9	40	251.9	253.3	1.39	207	17.05	25.4	1.2
(Zhou et al., 2009)	2006	Gansu, China	Silt loam	Maize	CK	0.17	231	0	231	403	0.04	225	0.76	20	1.36
(Zhou et al., 2009)	2006	Gansu, China	Silt loam	Maize	P	1.15	231	0	231	372	0.31	225	5.11	20	1.36
(Zhou et al., 2009)	2006	Gansu, China	Silt loam	Maize	P	1.12	231	0	231	397	0.28	225	4.98	20	1.36
(Zhou et al., 2009)	2007	Gansu, China	Silt loam	Maize	CK	0.54	287	0	287	397	0.14	225	2.38	20	1.36
(Zhou et al., 2009)	2007	Gansu, China	Silt loam	Maize	P	6.13	287	0	287	369	1.66	225	27.24	20	1.36
(Zhou et al., 2009)	2007	Gansu, China	Silt loam	Maize	P	3.72	287	0	287	376	0.99	225	16.53	20	1.36
(Zhou et al., 2009)	2007	Gansu, China	Silt loam	Maize	P	4.64	287	0	287	353	1.31	225	20.62	20	1.36
(Zhou et al., 2009)	2007	Gansu, China	Silt loam	Maize	P	5.03	287	0	287	331	1.52	225	22.35	20	1.36
(Sharma et al., 2011)	2005	Jammu, India	Sandy loam	Maize	CK	1.31	767.23	0	767.23	767.23	0.17	60	21.8	29.68	0.59
(Sharma et al., 2011)	2005	Jammu, India	Sandy loam	Maize	S	1.92	767.23	0	767.23	767.23	0.25	60	32	29.68	0.59
(Sharma et al., 2011)	2005	Jammu, India	Sandy loam	Maize	P	2.07	767.23	0	767.23	767.23	0.27	60	34.42	29.68	0.59
(Zhang et al., 2011)	2007	Shaanxi, China	Silt loam	Maize	CK	6.37	302	0	302	330	1.93	150	42.47	23.76	1.19
(Zhang et al., 2011)	2007	Shaanxi, China	Silt loam	Maize	S	7.25	302	0	302	301	2.41	150	48.34	23.76	1.19
(Zhang et al., 2011)	2007	Shaanxi, China	Silt loam	Maize	P	6.97	302	0	302	329	2.12	150	46.49	23.76	1.19
(Zhang et al., 2011)	2007	Shaanxi, China	Silt loam	Maize	P	7.2	302	0	302	321	2.24	150	47.97	23.76	1.19
(Zhang et al., 2011)	2008	Shaanxi, China	Silt loam	Maize	CK	7.06	340	0	340	261	2.71	150	47.09	23.76	1.19

(Zhang et al., 2011)	2008	Shaanxi, China	Silt loam	Maize	S	5.16	340	0	340	263	1.96	150	34.37	23.76	1.19
(Zhang et al., 2011)	2008	Shaanxi, China	Silt loam	Maize	P	7.66	340	0	340	282	2.72	150	51.05	23.76	1.19
(Zhang et al., 2011)	2008	Shaanxi, China	Silt loam	Maize	P	8.74	340	0	340	277	3.16	150	58.28	23.76	1.19
(Zhang et al., 2011)	2009	Shaanxi, China	Silt loam	Maize	CK	4.1	343	0	343	278	1.47	150	27.33	23.76	1.19
(Zhang et al., 2011)	2009	Shaanxi, China	Silt loam	Maize	S	4.3	343	0	343	281	1.53	150	28.67	23.76	1.19
(Zhang et al., 2011)	2009	Shaanxi, China	Silt loam	Maize	P	5.11	343	0	343	289	1.77	150	34.04	23.76	1.19
(Zhang et al., 2011)	2009	Shaanxi, China	Silt loam	Maize	P	4.98	343	0	343	286	1.74	150	33.22	23.76	1.19
(Bu et al., 2013)	2010	Shaanxi, China	Silt loam	Maize	CK	10.69	496	0	496	361.39	2.96	225	47.51	19	1.18
(Bu et al., 2013)	2010	Shaanxi, China	Silt loam	Maize	P	13.71	496	0	496	368.95	3.72	225	60.93	19	1.18
(Bu et al., 2013)	2011	Shaanxi, China	Silt loam	Maize	CK	7.14	487	0	487	351.72	2.03	225	31.73	19	1.18
(Bu et al., 2013)	2011	Shaanxi, China	Silt loam	Maize	P	13.39	487	0	487	350.71	3.82	225	59.51	19	1.18
(Li et al., 2013a)	2008	Shaanxi, China	Silt loam	Maize	P	11.79	330.3	0	330.3	436.5	2.7	150	78.61	19.34	1.09
(Li et al., 2013a)	2008	Shaanxi, China	Silt loam	Maize	P	11.85	330.3	0	330.3	439.3	2.7	150	78.98	19.34	1.09
(Li et al., 2013a)	2008	Shaanxi, China	Silt loam	Maize	S	11.52	30.3	0	330.3	434.1	2.65	150	76.78	19.34	1.09
(Li et al., 2013a)	2008	Shaanxi, China	Silt loam	Maize	P	10.56	330.3	0	330.3	420.5	2.51	150	70.4	19.34	1.09
(Li et al., 2013a)	2008	Shaanxi, China	Silt loam	Maize	CK	10.4	330.3	0	330.3	415.6	2.5	150	69.34	19.34	1.09
(Li et al., 2013a)	2009	Shaanxi, China	Silt loam	Maize	P	10.1	389.1	0	389.1	418.1	2.42	150	67.36	19.34	1.09
(Li et al., 2013a)	2009	Shaanxi, China	Silt loam	Maize	P	10.19	389.1	0	389.1	418.8	2.43	150	67.96	19.34	1.09
(Li et al., 2013a)	2009	Shaanxi, China	Silt loam	Maize	S	10.57	389.1	0	389.1	417.5	2.53	150	70.46	19.34	1.09
(Li et al., 2013a)	2009	Shaanxi, China	Silt loam	Maize	P	9.24	389.1	0	389.1	405.9	2.28	150	61.63	19.34	1.09
(Li et al., 2013a)	2009	Shaanxi, China	Silt loam	Maize	CK	9.02	389.1	0	389.1	405.8	2.22	150	60.14	19.34	1.09
(Li et al., 2013a)	2010	Shaanxi, China	Silt loam	Maize	P	10.71	431	0	431	419.3	2.55	150	71.4	19.34	1.09
(Li et al., 2013a)	2010	Shaanxi, China	Silt loam	Maize	P	10.8	431	0	431	421.1	2.56	150	72	19.34	1.09
(Li et al., 2013a)	2010	Shaanxi, China	Silt loam	Maize	S	11.04	431	0	431	422.1	2.61	150	73.57	19.34	1.09
(Li et al., 2013a)	2010	Shaanxi, China	Silt loam	Maize	P	9.5	431	0	431	411	2.31	150	63.35	19.34	1.09
(Li et al., 2013a)	2010	Shaanxi, China	Silt loam	Maize	CK	9.43	431	0	431	415.6	2.27	150	62.84	19.34	1.09

(Li et al., 2013a)	2007	Shaanxi, China	Silt loam	Maize	P	9.4	398	0	398	395	2.38	150	62.67	19.34	1.09
(Li et al., 2013a)	2007	Shaanxi, China	Silt loam	Maize	P	9.3	398	0	398	391	2.38	150	62	19.34	1.09
(Li et al., 2013a)	2007	Shaanxi, China	Silt loam	Maize	S	8.53	398	0	398	399	2.14	150	56.87	19.34	1.09
(Li et al., 2013a)	2007	Shaanxi, China	Silt loam	Maize	P	8.51	398	0	398	402	2.12	150	56.73	19.34	1.09
(Li et al., 2013a)	2007	Shaanxi, China	Silt loam	Maize	CK	8.19	398	0	398	400	2.05	150	54.6	19.34	1.09
(Li et al., 2013)	1992	Shaanxi, China	Silt loam	Maize	CK	2.21	265.1	40	305.1	313.2	0.71	0	NA	24.55	0.7
(Li et al., 2013)	1992	Shaanxi, China	Silt loam	Maize	CK	3.16	265.1	40	305.1	313.2	1.01	30	105.2	24.55	0.7
(Li et al., 2013)	1992	Shaanxi, China	Silt loam	Maize	CK	3.7	265.1	40	305.1	313.2	1.18	60	61.6	24.55	0.7
(Li et al., 2013)	1992	Shaanxi, China	Silt loam	Maize	CK	4.19	265.1	40	305.1	313.2	1.34	90	46.53	24.55	0.7
(Li et al., 2013)	1992	Shaanxi, China	Silt loam	Maize	CK	4.41	265.1	40	305.1	313.2	1.41	120	36.75	24.55	0.7
(Li et al., 2013)	1992	Shaanxi, China	Silt loam	Maize	P	3.77	265.1	40	305.1	316.3	1.19	0	NA	24.55	0.7
(Li et al., 2013)	1992	Shaanxi, China	Silt loam	Maize	P	4.94	265.1	40	305.1	316.3	1.56	30	164.8	24.55	0.7
(Li et al., 2013)	1992	Shaanxi, China	Silt loam	Maize	P	5.67	265.1	40	305.1	316.3	1.79	60	94.5	24.55	0.7
(Li et al., 2013)	1992	Shaanxi, China	Silt loam	Maize	P	6.67	265.1	40	305.1	316.3	2.11	90	74.13	24.55	0.7
(Li et al., 2013)	1992	Shaanxi, China	Silt loam	Maize	P	6.97	265.1	40	305.1	316.3	2.2	120	58.05	24.55	0.7
(Li et al., 2013)	1992	Shaanxi, China	Silt loam	Maize	S	3.91	265.1	40	305.1	314.9	1.24	0	NA	24.55	0.7
(Li et al., 2013)	1992	Shaanxi, China	Silt loam	Maize	S	3.99	265.1	40	305.1	314.9	1.27	30	133	24.55	0.7
(Li et al., 2013)	1992	Shaanxi, China	Silt loam	Maize	S	4.4	265.1	40	305.1	314.9	1.4	60	73.3	24.55	0.7
(Li et al., 2013)	1992	Shaanxi, China	Silt loam	Maize	S	5.05	265.1	40	305.1	314.9	1.6	90	56.13	24.55	0.7
(Li et al., 2013)	1992	Shaanxi, China	Silt loam	Maize	S	4.79	265.1	40	305.1	314.9	1.52	120	39.95	24.55	0.7
(Liu JL et al., 2014)	2010	Shaanxi, China	Silt loam	Maize	CK	10.59	496	0	496	364	2.14	225	47.08	19.3	1.64
(Liu JL et al., 2014)	2010	Shaanxi, China	Silt loam	Maize	P	13.63	496	0	496	369	2.75	225	60.56	19.3	1.64
(Liu JL et al., 2014)	2010	Shaanxi, China	Silt loam	Maize	P	14.81	496	0	496	388	2.99	225	65.82	19.3	1.64
(Liu JL et al., 2014)	2010	Shaanxi, China	Silt loam	Maize	P	15.83	496	0	496	338	3.19	225	70.33	19.3	1.64
(Liu JL et al., 2014)	2011	Shaanxi, China	Silt loam	Maize	CK	7.18	487	0	487	352	1.47	225	31.92	18.3	1.64
(Liu JL et al., 2014)	2011	Shaanxi, China	Silt loam	Maize	P	13.24	487	0	487	351	2.72	225	58.84	18.3	1.64

(Liu JL et al., 2014)	2011	Shaanxi, China	Silt loam	Maize	P	14.26	487	0	487	374	2.93	225	63.36	18.3	1.64
(Liu JL et al., 2014)	2011	Shaanxi, China	Silt loam	Maize	P	15.52	487	0	487	415	3.19	225	68.99	18.3	1.64
(Liu JL et al., 2014)	2012	Shaanxi, China	Silt loam	Maize	CK	9.65	358	0	358	385	2.7	225	42.9	19.3	1.64
(Liu JL et al., 2014)	2012	Shaanxi, China	Silt loam	Maize	P	13.27	358	0	358	379	3.71	225	58.99	19.3	1.64
(Liu JL et al., 2014)	2012	Shaanxi, China	Silt loam	Maize	P	14.54	358	0	358	430	4.06	225	64.63	19.3	1.64
(Liu JL et al., 2014)	2012	Shaanxi, China	Silt loam	Maize	P	15.14	358	0	358	430	4.23	225	67.27	19.3	1.64
(Gao et al., 2014)	2008	Shaanxi, China	Silt loam	Maize	P	9.84	269.3	0	269.3	269.3	3.65	390	25.23	18.34	1.93
(Gao et al., 2014)	2008	Shaanxi, China	Silt loam	Maize	P	9.42	269.3	0	269.3	269.3	3.5	390	24.16	18.34	1.93
(Gao et al., 2014)	2008	Shaanxi, China	Silt loam	Maize	P	7.44	269.3	0	269.3	269.3	2.76	390	19.08	18.34	1.93
(Gao et al., 2014)	2008	Shaanxi, China	Silt loam	Maize	P	6.63	269.3	0	269.3	269.3	2.46	390	16.99	18.34	1.93
(Gao et al., 2014)	2008	Shaanxi, China	Silt loam	Maize	CK	5.45	269.3	0	269.3	269.3	2.02	390	13.97	18.34	1.93
(Gao et al., 2014)	2009	Shaanxi, China	Silt loam	Maize	P	9.75	259.5	0	259.5	259.5	3.76	390	25	18.34	1.93
(Gao et al., 2014)	2009	Shaanxi, China	Silt loam	Maize	P	9.14	259.5	0	259.5	259.5	3.52	390	23.42	18.34	1.93
(Gao et al., 2014)	2009	Shaanxi, China	Silt loam	Maize	P	7.66	259.5	0	259.5	259.5	2.95	390	19.65	18.34	1.93
(Gao et al., 2014)	2009	Shaanxi, China	Silt loam	Maize	P	6.81	259.5	0	259.5	259.5	2.63	390	17.47	18.34	1.93
(Gao et al., 2014)	2009	Shaanxi, China	Silt loam	Maize	CK	5.12	259.5	0	259.5	259.5	1.97	390	13.12	18.34	1.93
(Han et al., 2013)	2008	Shaanxi, China	Silt loam	Maize	P	12.31	336.9	0	336.9	396.6	3.1	230	53.52	22.34	1.84
(Han et al., 2013)	2008	Shaanxi, China	Silt loam	Maize	P	12.29	336.9	0	336.9	402.1	3.06	230	53.44	22.34	1.84
(Han et al., 2013)	2008	Shaanxi, China	Silt loam	Maize	P	10.51	336.9	0	336.9	419.4	2.51	230	45.7	22.34	1.84
(Han et al., 2013)	2008	Shaanxi, China	Silt loam	Maize	CK	10.03	336.9	0	336.9	423.4	2.37	230	43.59	22.34	1.84
(Han et al., 2013)	2009	Shaanxi, China	Silt loam	Maize	P	11.46	391.6	0	391.6	365.2	3.14	230	49.84	22.34	1.84
(Han et al., 2013)	2009	Shaanxi, China	Silt loam	Maize	P	11.42	391.6	0	391.6	367.6	3.11	230	49.67	22.34	1.84
(Han et al., 2013)	2009	Shaanxi, China	Silt loam	Maize	P	10.25	391.6	0	391.6	385.5	2.66	230	44.56	22.34	1.84
(Han et al., 2013)	2009	Shaanxi, China	Silt loam	Maize	CK	9.79	391.6	0	391.6	367.1	2.67	230	42.55	22.34	1.84
(Han et al., 2013)	2010	Shaanxi, China	Silt loam	Maize	P	13.86	431	0	431	410	3.38	230	60.27	22.34	1.84
(Han et al., 2013)	2010	Shaanxi, China	Silt loam	Maize	P	13.6	431	0	431	408.6	3.33	230	59.14	22.34	1.84

(Han et al., 2013)	2010	Shaanxi, China	Silt loam	Maize	P	12.52	431	0	431	418.2	2.99	230	54.43	22.34	1.84
(Han et al., 2013)	2010	Shaanxi, China	Silt loam	Maize	CK	11.46	431	0	431	413.6	2.77	230	49.8	22.34	1.84
(Abd El-Wahed et al., 2013)	2009	Libya	Sandy	Maize	CK	6.78	0	560.66	560.66	560.66	1.21	0	NA	30.38	0.09
(Abd El-Wahed et al., 2013)	2009	Libya	Sandy	Maize	S	8.32	0	558.66	558.66	558.66	1.49	70	118.91	30.38	0.09
(Abd El-Wahed et al., 2013)	2009	Libya	Sandy	Maize	S	8.92	0	557.38	557.38	557.38	1.6	70	127.4	30.38	0.09
(Abd El-Wahed et al., 2013)	2009	Libya	Sandy	Maize	S	9.44	0	558.46	558.46	558.46	1.69	140	67.41	30.38	0.09
(Abd El-Wahed et al., 2013)	2009	Libya	Sandy	Maize	S	10.07	0	559.5	559.5	559.5	1.8	140	71.94	30.38	0.09
(Abd El-Wahed et al., 2013)	2009	Libya	Sandy	Maize	CK	4.89	0	474.66	474.66	474.66	1.03	0	NA	30.38	0.09
(Abd El-Wahed et al., 2013)	2009	Libya	Sandy	Maize	S	5.8	0	475.66	475.66	475.66	1.22	70	82.9	30.38	0.09
(Abd El-Wahed et al., 2013)	2009	Libya	Sandy	Maize	S	6.16	0	473.85	473.85	473.85	1.3	70	88	30.38	0.09
(Abd El-Wahed et al., 2013)	2009	Libya	Sandy	Maize	S	6.95	0	476.23	476.23	476.23	1.46	140	49.66	30.38	0.09
(Abd El-Wahed et al., 2013)	2009	Libya	Sandy	Maize	S	7.86	0	476.24	476.24	476.24	1.65	140	56.13	30.38	0.09
(Abd El-Wahed et al., 2013)	2009	Libya	Sandy	Maize	CK	4.2	0	392.71	392.71	392.71	1.07	0	NA	30.38	0.09
(Abd El-Wahed et al., 2013)	2009	Libya	Sandy	Maize	S	4.85	0	391.21	391.21	391.21	1.24	70	69.3	30.38	0.09
(Abd El-Wahed et al., 2013)	2009	Libya	Sandy	Maize	S	5.15	0	390.15	390.15	390.15	1.32	70	73.57	30.38	0.09
(Abd El-Wahed et al., 2013)	2009	Libya	Sandy	Maize	S	5.45	0	392.23	392.23	392.23	1.39	140	38.94	30.38	0.09
(Abd El-Wahed et al., 2013)	2009	Libya	Sandy	Maize	S	5.97	0	390.07	390.07	390.07	1.53	140	42.63	30.38	0.09
(Abd El-Wahed et al., 2013)	2009	Libya	Sandy	Maize	CK	5.12	0	682	682	682	0.75	0	NA	30.38	0.09
(Abd El-Wahed et al., 2013)	2009	Libya	Sandy	Maize	S	5.93	0	681.95	681.95	681.95	0.87	70	84.76	30.38	0.09
(Abd El-Wahed et al., 2013)	2009	Libya	Sandy	Maize	S	6.27	0	681.96	681.96	681.96	0.92	70	89.63	30.38	0.09
(Abd El-Wahed et al., 2013)	2009	Libya	Sandy	Maize	S	7.71	0	681.95	681.95	681.95	1.13	140	55.04	30.38	0.09
(Abd El-Wahed et al., 2013)	2009	Libya	Sandy	Maize	S	8.05	0	681.95	681.95	681.95	1.18	140	57.48	30.38	0.09
(Abd El-Wahed et al., 2013)	2009	Libya	Sandy	Maize	CK	3.88	0	579.7	579.7	579.7	0.67	0	NA	30.38	0.09
(Abd El-Wahed et al., 2013)	2009	Libya	Sandy	Maize	S	4.29	0	579.73	579.73	579.73	0.74	70	61.29	30.38	0.09
(Abd El-Wahed et al., 2013)	2009	Libya	Sandy	Maize	S	4.75	0	579.63	579.63	579.63	0.82	70	67.9	30.38	0.09
(Abd El-Wahed et al., 2013)	2009	Libya	Sandy	Maize	S	5.45	0	579.68	579.68	579.68	0.94	140	38.92	30.38	0.09

(Abd El-Wahed et al., 2013)	2009	Libya	Sandy	Maize	S	6.43	0	579.64	579.64	579.64	1.11	140	45.96	30.38	0.09
(Abd El-Wahed et al., 2013)	2009	Libya	Sandy	Maize	CK	2.86	0	477.33	477.33	477.33	0.6	0	NA	30.38	0.09
(Abd El-Wahed et al., 2013)	2009	Libya	Sandy	Maize	S	3.49	0	477.4	477.4	477.4	0.73	70	49.79	30.38	0.09
(Abd El-Wahed et al., 2013)	2009	Libya	Sandy	Maize	S	4.11	0	477.44	477.44	477.44	0.86	70	58.66	30.38	0.09
(Abd El-Wahed et al., 2013)	2009	Libya	Sandy	Maize	S	4.77	0	477.4	477.4	477.4	1	140	34.1	30.38	0.09
(Abd El-Wahed et al., 2013)	2009	Libya	Sandy	Maize	S	5.11	0	477.38	477.38	477.38	1.07	140	36.49	30.38	0.09
(Abd El-Wahed et al., 2013)	2010	Libya	Sandy	Maize	CK	6.81	0	558.2	558.2	558.2	1.22	0	NA	30.38	0.09
(Abd El-Wahed et al., 2013)	2010	Libya	Sandy	Maize	S	8.45	0	559.27	559.27	559.27	1.51	70	120.64	30.38	0.09
(Abd El-Wahed et al., 2013)	2010	Libya	Sandy	Maize	S	9	0	558.94	558.94	558.94	1.61	70	128.56	30.38	0.09
(Abd El-Wahed et al., 2013)	2010	Libya	Sandy	Maize	S	9.59	0	557.5	557.5	557.5	1.72	140	68.49	30.38	0.09
(Abd El-Wahed et al., 2013)	2010	Libya	Sandy	Maize	S	10.1	0	558.01	558.01	558.01	1.81	140	72.14	30.38	0.09
(Abd El-Wahed et al., 2013)	2010	Libya	Sandy	Maize	CK	4.91	0	476.7	476.7	476.7	1.03	0	NA	30.38	0.09
(Abd El-Wahed et al., 2013)	2010	Libya	Sandy	Maize	S	6	0	476.11	476.11	476.11	1.26	70	85.7	30.38	0.09
(Abd El-Wahed et al., 2013)	2010	Libya	Sandy	Maize	S	6.27	0	475	475	475	1.32	70	89.57	30.38	0.09
(Abd El-Wahed et al., 2013)	2010	Libya	Sandy	Maize	S	7.2	0	476.49	476.49	476.49	1.51	140	51.39	30.38	0.09
(Abd El-Wahed et al., 2013)	2010	Libya	Sandy	Maize	S	8.06	0	474	474	474	1.7	140	57.56	30.38	0.09
(Abd El-Wahed et al., 2013)	2010	Libya	Sandy	Maize	CK	4.35	0	391.89	391.89	391.89	1.11	0	NA	30.38	0.09
(Abd El-Wahed et al., 2013)	2010	Libya	Sandy	Maize	S	4.93	0	390.87	390.87	390.87	1.26	70	70.36	30.38	0.09
(Abd El-Wahed et al., 2013)	2010	Libya	Sandy	Maize	S	5.25	0	391.79	391.79	391.79	1.34	70	75	30.38	0.09
(Abd El-Wahed et al., 2013)	2010	Libya	Sandy	Maize	S	5.56	0	391.55	391.55	391.55	1.42	140	39.71	30.38	0.09
(Abd El-Wahed et al., 2013)	2010	Libya	Sandy	Maize	S	6	0	392.16	392.16	392.16	1.53	140	42.86	30.38	0.09
(Abd El-Wahed et al., 2013)	2010	Libya	Sandy	Maize	CK	5.18	0	681.97	681.97	681.97	0.76	0	NA	30.38	0.09
(Abd El-Wahed et al., 2013)	2010	Libya	Sandy	Maize	S	6.07	0	682.02	682.02	682.02	0.89	70	86.71	30.38	0.09
(Abd El-Wahed et al., 2013)	2010	Libya	Sandy	Maize	S	6.41	0	682.02	682.02	682.02	0.94	70	91.59	30.38	0.09
(Abd El-Wahed et al., 2013)	2010	Libya	Sandy	Maize	S	7.78	0	682.02	682.02	682.02	1.14	140	55.54	30.38	0.09
(Abd El-Wahed et al., 2013)	2010	Libya	Sandy	Maize	S	8.05	0	681.95	681.95	681.95	1.18	140	57.48	30.38	0.09

(Abd El-Wahed et al., 2013)	2010	Libya	Sandy	Maize	CK	4	0	579.71	579.71	579.71	0.69	0	NA	30.38	0.09
(Abd El-Wahed et al., 2013)	2010	Libya	Sandy	Maize	S	4.35	0	579.73	579.73	579.73	0.75	70	62.11	30.38	0.09
(Abd El-Wahed et al., 2013)	2010	Libya	Sandy	Maize	S	4.87	0	579.64	579.64	579.64	0.84	70	69.56	30.38	0.09
(Abd El-Wahed et al., 2013)	2010	Libya	Sandy	Maize	S	5.57	0	579.69	579.69	579.69	0.96	140	39.75	30.38	0.09
(Abd El-Wahed et al., 2013)	2010	Libya	Sandy	Maize	S	6.49	0	579.64	579.64	579.64	1.12	140	46.37	30.38	0.09
(Abd El-Wahed et al., 2013)	2010	Libya	Sandy	Maize	CK	3.06	0	477.34	477.34	477.34	0.64	0	NA	30.38	0.09
(Abd El-Wahed et al., 2013)	2010	Libya	Sandy	Maize	S	3.53	0	477.43	477.43	477.43	0.74	70	50.47	30.38	0.09
(Abd El-Wahed et al., 2013)	2010	Libya	Sandy	Maize	S	4.25	0	477.42	477.42	477.42	0.89	70	60.7	30.38	0.09
(Abd El-Wahed et al., 2013)	2010	Libya	Sandy	Maize	S	5.01	0	477.43	477.43	477.43	1.05	140	35.81	30.38	0.09
(Abd El-Wahed et al., 2013)	2010	Libya	Sandy	Maize	S	5.25	0	477.36	477.36	477.36	1.1	140	37.51	30.38	0.09
(Shen et al., 2012)	2009	Shandong, China	loamy	Maize	CK	7.7	476	0	476	437.4	1.76	150	51.36	25.8	2.05
(Shen et al., 2012)	2009	Shandong, China	loamy	Maize	S	8.75	476	0	476	435	2.01	150	58.36	25.8	2.05
(Shen et al., 2012)	2009	Shandong, China	loamy	Maize	S	9.08	476	0	476	454.36	2	150	60.56	25.8	2.05
(Shen et al., 2012)	2009	Shandong, China	loamy	Maize	CK	8.84	476	0	476	429.3	2.06	150	58.93	25.8	2.05
(Shen et al., 2012)	2009	Shandong, China	loamy	Maize	S	8.95	476	0	476	438.1	2.04	150	59.69	25.8	2.05
(Shen et al., 2012)	2009	Shandong, China	loamy	Maize	S	10.9	476	0	476	450.7	2.42	150	72.69	25.8	2.05
(Shen et al., 2012)	2010	Shandong, China	loamy	Maize	CK	10.98	488.7	0	488.7	352.8	3.11	150	73.21	25.8	2.05
(Shen et al., 2012)	2010	Shandong, China	loamy	Maize	S	11.85	488.7	0	488.7	375	3.16	150	79.01	25.8	2.05
(Shen et al., 2012)	2010	Shandong, China	loamy	Maize	S	11.71	488.7	0	488.7	369.3	3.17	150	78.09	25.8	2.05
(Shen et al., 2012)	2010	Shandong, China	loamy	Maize	CK	13.23	488.7	0	488.7	351.2	3.77	150	88.21	25.8	2.05
(Shen et al., 2012)	2010	Shandong, China	loamy	Maize	S	13.27	488.7	0	488.7	409.2	3.24	150	88.5	25.8	2.05
(Shen et al., 2012)	2010	Shandong, China	loamy	Maize	S	12.13	488.7	0	488.7	383.4	3.16	150	80.86	25.8	2.05
(Ram et al., 2012)	2003	Ludhiana, India	Sandy loam	Maize	CK	5.54	706	0	706	706	0.81	120	46.17	26.16	0.72
(Ram et al., 2012)	2003	Ludhiana, India	Sandy loam	Maize	CK	5.5	706	0	706	706	0.79	120	45.83	26.16	0.72
(Ram et al., 2012)	2003	Ludhiana, India	Sandy loam	Maize	CK	5.57	701	0	701	701	0.8	120	46.42	26.16	0.72
(Ram et al., 2012)	2003	Ludhiana, India	Sandy loam	Maize	S	5.68	694	0	694	694	0.83	120	47.33	26.16	0.72

(Ram et al., 2012)	2003	Ludhiana, India	Sandy loam	Maize	CK	5.57	659	0	659	659	0.87	120	46.42	26.16	0.72
(Ram et al., 2012)	2003	Ludhiana, India	Sandy loam	Maize	CK	5.55	708	0	708	708	0.78	120	46.25	26.16	0.72
(Ram et al., 2012)	2003	Ludhiana, India	Sandy loam	Maize	CK	5.62	660	0	660	660	0.86	120	46.83	26.16	0.72
(Ram et al., 2012)	2003	Ludhiana, India	Sandy loam	Maize	S	5.75	655	0	655	655	0.89	120	47.92	26.16	0.72
(Mupangwa et al., 2012)	2006	Zimbabwe	Silty clay loam	Maize	CK	0.68	250	0	250	250	0.27	149	4.54	21	0.45
(Mupangwa et al., 2012)	2006	Zimbabwe	Silty clay loam	Maize	S	1.05	250	0	250	250	0.42	149	7.01	21	0.45
(Mupangwa et al., 2012)	2006	Zimbabwe	Silty clay loam	Maize	S	1.71	250	0	250	250	0.68	149	11.49	21	0.45
(Mupangwa et al., 2012)	2006	Zimbabwe	Silty clay loam	Maize	S	1.54	250	0	250	250	0.61	149	10.31	21	0.45
(Mupangwa et al., 2012)	2006	Zimbabwe	Silty clay loam	Maize	S	0.9	250	0	250	250	0.36	149	6.05	21	0.45
(Mupangwa et al., 2012)	2006	Zimbabwe	Silty clay loam	Maize	S	2.02	250	0	250	250	0.81	149	13.54	21	0.45
(Mupangwa et al., 2012)	2006	Zimbabwe	Silty clay loam	Maize	S	1.55	250	0	250	250	0.62	149	10.43	21	0.45
(Mupangwa et al., 2012)	2006	Zimbabwe	Silty clay loam	Maize	CK	1.06	250	0	250	250	0.42	149	7.11	21	0.45
(Mupangwa et al., 2012)	2006	Zimbabwe	Silty clay loam	Maize	S	1.15	250	0	250	250	0.46	149	7.7	21	0.45
(Mupangwa et al., 2012)	2006	Zimbabwe	Silty clay loam	Maize	S	0.92	250	0	250	250	0.37	149	6.17	21	0.45
(Mupangwa et al., 2012)	2006	Zimbabwe	Silty clay loam	Maize	S	1.46	250	0	250	250	0.58	149	9.77	21	0.45
(Mupangwa et al., 2012)	2006	Zimbabwe	Silty clay loam	Maize	S	2.03	250	0	250	250	0.81	149	13.62	21	0.45
(Mupangwa et al., 2012)	2006	Zimbabwe	Silty clay loam	Maize	S	1.86	250	0	250	250	0.74	149	12.46	21	0.45
(Mupangwa et al., 2012)	2006	Zimbabwe	Silty clay loam	Maize	S	1.75	250	0	250	250	0.7	149	11.75	21	0.45
(Mupangwa et al., 2012)	2006	Zimbabwe	Silty clay loam	Maize	CK	0.74	250	0	250	250	0.29	149	4.93	21	0.45
(Mupangwa et al., 2012)	2006	Zimbabwe	Silty clay loam	Maize	S	0.97	250	0	250	250	0.39	149	6.53	21	0.45
(Mupangwa et al., 2012)	2006	Zimbabwe	Silty clay loam	Maize	S	1.21	250	0	250	250	0.48	149	8.12	21	0.45
(Mupangwa et al., 2012)	2006	Zimbabwe	Silty clay loam	Maize	S	0.93	250	0	250	250	0.37	149	6.23	21	0.45
(Mupangwa et al., 2012)	2006	Zimbabwe	Silty clay loam	Maize	S	1.57	250	0	250	250	0.63	149	10.54	21	0.45
(Mupangwa et al., 2012)	2006	Zimbabwe	Silty clay loam	Maize	S	2.12	250	0	250	250	0.85	149	14.25	21	0.45
(Mupangwa et al., 2012)	2006	Zimbabwe	Silty clay loam	Maize	S	2.13	250	0	250	250	0.85	149	14.29	21	0.45
(Mupangwa et al., 2012)	2007	Zimbabwe	Silty clay loam	Maize	CK	1.5	364	0	364	364	0.41	149	10.09	21	0.45

(Mupangwa et al., 2012)	2007	Zimbabwe	Silty clay loam	Maize	S	1.51	364	0	364	364	0.41	149	10.11	21	0.45
(Mupangwa et al., 2012)	2007	Zimbabwe	Silty clay loam	Maize	S	1.13	364	0	364	364	0.31	149	7.58	21	0.45
(Mupangwa et al., 2012)	2007	Zimbabwe	Silty clay loam	Maize	S	0.66	364	0	364	364	0.18	149	4.46	21	0.45
(Mupangwa et al., 2012)	2007	Zimbabwe	Silty clay loam	Maize	S	1.22	364	0	364	364	0.33	149	8.17	21	0.45
(Mupangwa et al., 2012)	2007	Zimbabwe	Silty clay loam	Maize	S	1.16	364	0	364	364	0.32	149	7.81	21	0.45
(Mupangwa et al., 2012)	2007	Zimbabwe	Silty clay loam	Maize	S	1.88	364	0	364	364	0.52	149	12.6	21	0.45
(Mupangwa et al., 2012)	2007	Zimbabwe	Silty clay loam	Maize	CK	1.26	364	0	364	364	0.35	149	8.44	21	0.45
(Mupangwa et al., 2012)	2007	Zimbabwe	Silty clay loam	Maize	S	2.21	364	0	364	364	0.61	149	14.84	21	0.45
(Mupangwa et al., 2012)	2007	Zimbabwe	Silty clay loam	Maize	S	1.19	364	0	364	364	0.33	149	8.01	21	0.45
(Mupangwa et al., 2012)	2007	Zimbabwe	Silty clay loam	Maize	S	1.2	364	0	364	364	0.33	149	8.07	21	0.45
(Mupangwa et al., 2012)	2007	Zimbabwe	Silty clay loam	Maize	S	1.16	364	0	364	364	0.32	149	7.8	21	0.45
(Mupangwa et al., 2012)	2007	Zimbabwe	Silty clay loam	Maize	S	1.69	364	0	364	364	0.46	149	11.32	21	0.45
(Mupangwa et al., 2012)	2007	Zimbabwe	Silty clay loam	Maize	S	1.1	364	0	364	364	0.3	149	7.38	21	0.45
(Mupangwa et al., 2012)	2007	Zimbabwe	Silty clay loam	Maize	CK	1.12	364	0	364	364	0.31	149	7.54	21	0.45
(Mupangwa et al., 2012)	2007	Zimbabwe	Silty clay loam	Maize	S	0.96	364	0	364	364	0.26	149	6.47	21	0.45
(Mupangwa et al., 2012)	2007	Zimbabwe	Silty clay loam	Maize	S	0.97	364	0	364	364	0.27	149	6.52	21	0.45
(Mupangwa et al., 2012)	2007	Zimbabwe	Silty clay loam	Maize	S	1.01	364	0	364	364	0.28	149	6.77	21	0.45
(Mupangwa et al., 2012)	2007	Zimbabwe	Silty clay loam	Maize	S	1.08	364	0	364	364	0.3	149	7.22	21	0.45
(Mupangwa et al., 2012)	2007	Zimbabwe	Silty clay loam	Maize	S	0.78	364	0	364	364	0.21	149	5.24	21	0.45
(Mupangwa et al., 2012)	2007	Zimbabwe	Silty clay loam	Maize	S	0.52	364	0	364	364	0.14	149	3.48	21	0.45
(Mupangwa et al., 2012)	2007	Zimbabwe	Silty clay loam	Maize	CK	1.37	364	0	364	364	0.38	149	9.19	21	0.45
(Mupangwa et al., 2012)	2007	Zimbabwe	Silty clay loam	Maize	S	1.37	364	0	364	364	0.38	149	9.17	21	0.45
(Mupangwa et al., 2012)	2007	Zimbabwe	Silty clay loam	Maize	S	1.46	364	0	364	364	0.4	149	9.82	21	0.45
(Mupangwa et al., 2012)	2007	Zimbabwe	Silty clay loam	Maize	S	1.35	364	0	364	364	0.37	149	9.05	21	0.45
(Mupangwa et al., 2012)	2007	Zimbabwe	Silty clay loam	Maize	S	1.02	364	0	364	364	0.28	149	6.85	21	0.45
(Mupangwa et al., 2012)	2007	Zimbabwe	Silty clay loam	Maize	S	1	364	0	364	364	0.27	149	6.69	21	0.45

(Mupangwa et al., 2012)	2007	Zimbabwe	Silty clay loam	Maize	S	1.15	364	0	364	364	0.32	149	7.7	21	0.45
(Mupangwa et al., 2012)	2007	Zimbabwe	Silty clay loam	Maize	CK	1	364	0	364	364	0.27	149	6.7	21	0.45
(Mupangwa et al., 2012)	2007	Zimbabwe	Silty clay loam	Maize	S	1.23	364	0	364	364	0.34	149	8.26	21	0.45
(Mupangwa et al., 2012)	2007	Zimbabwe	Silty clay loam	Maize	S	1.04	364	0	364	364	0.28	149	6.96	21	0.45
(Mupangwa et al., 2012)	2007	Zimbabwe	Silty clay loam	Maize	S	1.24	364	0	364	364	0.34	149	8.35	21	0.45
(Mupangwa et al., 2012)	2007	Zimbabwe	Silty clay loam	Maize	S	0.87	364	0	364	364	0.24	149	5.81	21	0.45
(Mupangwa et al., 2012)	2007	Zimbabwe	Silty clay loam	Maize	S	1.16	364	0	364	364	0.32	149	7.81	21	0.45
(Mupangwa et al., 2012)	2007	Zimbabwe	Silty clay loam	Maize	S	1.14	364	0	364	364	0.31	149	7.66	21	0.45
(Mupangwa et al., 2012)	2007	Zimbabwe	Silty clay loam	Maize	CK	0.93	364	0	364	364	0.25	149	6.21	21	0.45
(Mupangwa et al., 2012)	2007	Zimbabwe	Silty clay loam	Maize	S	1.13	364	0	364	364	0.31	149	7.6	21	0.45
(Mupangwa et al., 2012)	2007	Zimbabwe	Silty clay loam	Maize	S	0.88	364	0	364	364	0.24	149	5.91	21	0.45
(Mupangwa et al., 2012)	2007	Zimbabwe	Silty clay loam	Maize	S	1.24	364	0	364	364	0.34	149	8.34	21	0.45
(Mupangwa et al., 2012)	2007	Zimbabwe	Silty clay loam	Maize	S	0.84	364	0	364	364	0.23	149	5.66	21	0.45
(Mupangwa et al., 2012)	2007	Zimbabwe	Silty clay loam	Maize	S	1.01	364	0	364	364	0.28	149	6.78	21	0.45
(Mupangwa et al., 2012)	2007	Zimbabwe	Silty clay loam	Maize	S	1.05	364	0	364	364	0.29	149	7.07	21	0.45
(Mupangwa et al., 2012)	2007	Zimbabwe	Silty clay loam	Maize	CK	2.97	364	0	364	364	0.82	197	15.08	21	0.45
(Mupangwa et al., 2012)	2007	Zimbabwe	Silty clay loam	Maize	S	2.4	364	0	364	364	0.66	197	12.18	21	0.45
(Mupangwa et al., 2012)	2007	Zimbabwe	Silty clay loam	Maize	S	2.53	364	0	364	364	0.69	197	12.83	21	0.45
(Mupangwa et al., 2012)	2007	Zimbabwe	Silty clay loam	Maize	S	2.87	364	0	364	364	0.79	197	14.55	21	0.45
(Mupangwa et al., 2012)	2007	Zimbabwe	Silty clay loam	Maize	S	2.15	364	0	364	364	0.59	197	10.9	21	0.45
(Mupangwa et al., 2012)	2007	Zimbabwe	Silty clay loam	Maize	S	2.56	364	0	364	364	0.7	197	12.99	21	0.45
(Mupangwa et al., 2012)	2007	Zimbabwe	Silty clay loam	Maize	S	2.41	364	0	364	364	0.66	197	12.22	21	0.45
(Mupangwa et al., 2012)	2007	Zimbabwe	Silty clay loam	Maize	CK	2.03	364	0	364	364	0.56	197	10.32	21	0.45
(Mupangwa et al., 2012)	2007	Zimbabwe	Silty clay loam	Maize	S	2.82	364	0	364	364	0.77	197	14.31	21	0.45
(Mupangwa et al., 2012)	2007	Zimbabwe	Silty clay loam	Maize	S	2.24	364	0	364	364	0.61	197	11.36	21	0.45
(Mupangwa et al., 2012)	2007	Zimbabwe	Silty clay loam	Maize	S	1.85	364	0	364	364	0.51	197	9.4	21	0.45

(Mupangwa et al., 2012)	2007	Zimbabwe	Silty clay loam	Maize	S	1.7	364	0	364	364	0.47	197	8.61	21	0.45
(Mupangwa et al., 2012)	2007	Zimbabwe	Silty clay loam	Maize	S	1.76	364	0	364	364	0.48	197	8.95	21	0.45
(Mupangwa et al., 2012)	2007	Zimbabwe	Silty clay loam	Maize	S	1.55	364	0	364	364	0.43	197	7.85	21	0.45
(Mupangwa et al., 2012)	2007	Zimbabwe	Silty clay loam	Maize	CK	3.14	364	0	364	364	0.86	197	15.93	21	0.45
(Mupangwa et al., 2012)	2007	Zimbabwe	Silty clay loam	Maize	S	2.62	364	0	364	364	0.72	197	13.29	21	0.45
(Mupangwa et al., 2012)	2007	Zimbabwe	Silty clay loam	Maize	S	2.32	364	0	364	364	0.64	197	11.77	21	0.45
(Mupangwa et al., 2012)	2007	Zimbabwe	Silty clay loam	Maize	S	2.47	364	0	364	364	0.68	197	12.53	21	0.45
(Mupangwa et al., 2012)	2007	Zimbabwe	Silty clay loam	Maize	S	1.69	364	0	364	364	0.46	197	8.57	21	0.45
(Mupangwa et al., 2012)	2007	Zimbabwe	Silty clay loam	Maize	S	2.44	364	0	364	364	0.67	197	12.38	21	0.45
(Mupangwa et al., 2012)	2007	Zimbabwe	Silty clay loam	Maize	S	2.2	364	0	364	364	0.6	197	11.14	21	0.45
(Mupangwa et al., 2012)	2007	Zimbabwe	Silty clay loam	Maize	CK	2.4	364	0	364	364	0.66	197	12.18	21	0.45
(Mupangwa et al., 2012)	2007	Zimbabwe	Silty clay loam	Maize	S	2.79	364	0	364	364	0.77	197	14.17	21	0.45
(Mupangwa et al., 2012)	2007	Zimbabwe	Silty clay loam	Maize	S	2.28	364	0	364	364	0.63	197	11.56	21	0.45
(Mupangwa et al., 2012)	2007	Zimbabwe	Silty clay loam	Maize	S	1.98	364	0	364	364	0.54	197	10.04	21	0.45
(Mupangwa et al., 2012)	2007	Zimbabwe	Silty clay loam	Maize	S	3.57	364	0	364	364	0.98	197	18.12	21	0.45
(Mupangwa et al., 2012)	2007	Zimbabwe	Silty clay loam	Maize	S	3.53	364	0	364	364	0.97	197	17.89	21	0.45
(Mupangwa et al., 2012)	2007	Zimbabwe	Silty clay loam	Maize	S	2.88	364	0	364	364	0.79	197	14.62	21	0.45
(Mupangwa et al., 2012)	2007	Zimbabwe	Silty clay loam	Maize	CK	2.58	364	0	364	364	0.71	197	13.09	21	0.45
(Mupangwa et al., 2012)	2007	Zimbabwe	Silty clay loam	Maize	S	3.33	364	0	364	364	0.91	197	16.89	21	0.45
(Mupangwa et al., 2012)	2007	Zimbabwe	Silty clay loam	Maize	S	2.41	364	0	364	364	0.66	197	12.23	21	0.45
(Mupangwa et al., 2012)	2007	Zimbabwe	Silty clay loam	Maize	S	3.33	364	0	364	364	0.91	197	16.89	21	0.45
(Mupangwa et al., 2012)	2007	Zimbabwe	Silty clay loam	Maize	S	2.69	364	0	364	364	0.74	197	13.64	21	0.45
(Mupangwa et al., 2012)	2007	Zimbabwe	Silty clay loam	Maize	S	2.39	364	0	364	364	0.66	197	12.15	21	0.45
(Mupangwa et al., 2012)	2007	Zimbabwe	Silty clay loam	Maize	S	2.72	364	0	364	364	0.75	197	13.8	21	0.45
(Mupangwa et al., 2012)	2007	Zimbabwe	Silty clay loam	Maize	CK	3.23	364	0	364	364	0.89	197	16.38	21	0.45
(Mupangwa et al., 2012)	2007	Zimbabwe	Silty clay loam	Maize	S	3.48	364	0	364	364	0.96	197	17.68	21	0.45

(Mupangwa et al., 2012)	2007	Zimbabwe	Silty clay loam	Maize	S	3.73	364	0	364	364	1.02	197	18.92	21	0.45
(Mupangwa et al., 2012)	2007	Zimbabwe	Silty clay loam	Maize	S	3.59	364	0	364	364	0.99	197	18.23	21	0.45
(Mupangwa et al., 2012)	2007	Zimbabwe	Silty clay loam	Maize	S	2.8	364	0	364	364	0.77	197	14.21	21	0.45
(Mupangwa et al., 2012)	2007	Zimbabwe	Silty clay loam	Maize	S	3.6	364	0	364	364	0.99	197	18.26	21	0.45
(Mupangwa et al., 2012)	2007	Zimbabwe	Silty clay loam	Maize	S	3.67	364	0	364	364	1.01	197	18.61	21	0.45
(Khaledian et al., 2012)	2001	France	Loamy	Maize	CK	10.9	141	206	347	497	2.19	120	90.83	21	1.34
(Khaledian et al., 2012)	2001	France	Loamy	Maize	S	7.95	141	216	357	459	1.73	126	63.1	21	1.34
(Khaledian et al., 2012)	2002	France	Loamy	Maize	CK	11.9	311	346	657	544	2.19	190	62.63	21	1.34
(Khaledian et al., 2012)	2002	France	Loamy	Maize	S	10.68	311	292	603	504	2.12	173	61.73	21	1.34
(Khaledian et al., 2012)	2007	France	Loamy	Maize	CK	13.8	203	218	421	580	2.38	180	76.67	21	1.34
(Khaledian et al., 2012)	2007	France	Loamy	Maize	S	12.85	203	182	385	504	2.55	180	71.39	21	1.34
(Botha et al., 2012)	2000	South Africa	Clay	Maize	CK	3.09	228	0	228	266.67	1.16	43	71.93	18.3	1.29
(Botha et al., 2012)	2000	South Africa	Clay	Maize	S	3.46	228	0	228	319.51	1.08	43	80.35	18.3	1.29
(Botha et al., 2012)	2000	South Africa	Clay	Maize	S	3.52	228	0	228	290.91	1.21	43	81.84	18.3	1.29
(Botha et al., 2012)	2000	South Africa	Clay	Maize	S	3.96	228	0	228	297.83	1.33	43	92.14	18.3	1.29
(Botha et al., 2012)	2000	South Africa	Clay	Maize	S	3.5	228	0	228	310	1.13	43	81.4	18.3	1.29
(Botha et al., 2012)	2001	South Africa	Clay	Maize	CK	1.49	281	0	281	256	0.58	43	34.63	18.3	1.29
(Botha et al., 2012)	2001	South Africa	Clay	Maize	S	2.54	281	0	281	267.57	0.95	43	59.14	18.3	1.29
(Botha et al., 2012)	2001	South Africa	Clay	Maize	S	2.91	281	0	281	225.49	1.29	43	67.63	18.3	1.29
(Botha et al., 2012)	2001	South Africa	Clay	Maize	S	3.1	281	0	281	251.02	1.23	43	72.05	18.3	1.29
(Botha et al., 2012)	2001	South Africa	Clay	Maize	S	2.73	281	0	281	240.43	1.14	43	63.51	18.3	1.29
(Botha et al., 2012)	2002	South Africa	Clay	Maize	CK	1.52	247	0	247	325	0.47	43	35.37	18.3	1.29
(Botha et al., 2012)	2002	South Africa	Clay	Maize	S	3.28	247	0	247	342.42	0.96	43	76.3	18.3	1.29
(Botha et al., 2012)	2002	South Africa	Clay	Maize	S	3.33	247	0	247	332.43	1	43	77.33	18.3	1.29
(Botha et al., 2012)	2002	South Africa	Clay	Maize	S	3.61	247	0	247	336.11	1.07	43	83.88	18.3	1.29
(Botha et al., 2012)	2002	South Africa	Clay	Maize	S	3.29	247	0	247	341.18	0.96	43	76.47	18.3	1.29

(Botha et al., 2012)	2003	South Africa	Clay	Maize	CK	0.46	215	0	215	200	0.23	43	10.67	18.3	1.29
(Botha et al., 2012)	2003	South Africa	Clay	Maize	S	2.4	215	0	215	327.5	0.73	43	55.84	18.3	1.29
(Botha et al., 2012)	2003	South Africa	Clay	Maize	S	3.07	215	0	215	351.16	0.87	43	71.3	18.3	1.29
(Botha et al., 2012)	2003	South Africa	Clay	Maize	S	3.27	215	0	215	370.45	0.88	43	76.09	18.3	1.29
(Botha et al., 2012)	2003	South Africa	Clay	Maize	S	2.95	215	0	215	350	0.84	43	68.65	18.3	1.29
(Wang et al., 2011)	2006	Henan,China	Sandy loam	Maize	CK	6.2	476.9	0	476.9	426.92	1.3	184	33.7	25.14	1.36
(Wang et al., 2011)	2006	Henan,China	Sandy loam	Maize	S	7.08	476.9	0	476.9	410.24	1.48	184	38.48	25.14	1.36
(Wang et al., 2011)	2006	Henan,China	Sandy loam	Maize	CK	6.93	476.9	0	476.9	410.63	1.45	184	37.64	25.14	1.36
(Wang et al., 2011)	2006	Henan,China	Sandy loam	Maize	S	7.59	476.9	0	476.9	402.84	1.59	184	41.23	25.14	1.36
(Wang et al., 2011)	2007	Henan,China	Sandy loam	Maize	CK	4.8	443	0	443	461.38	1.08	184	26.11	25.14	1.36
(Wang et al., 2011)	2007	Henan,China	Sandy loam	Maize	S	5.3	443	0	443	460.45	1.2	184	28.82	25.14	1.36
(Wang et al., 2011)	2007	Henan,China	Sandy loam	Maize	CK	4.94	443	0	443	450.69	1.12	184	26.85	25.14	1.36
(Wang et al., 2011)	2007	Henan,China	Sandy loam	Maize	S	6.14	443	0	443	451.59	1.39	184	33.38	25.14	1.36
(Easson et al., 2000)	1996	UK	Sandy clay loam	Maize	CK	1.16	448	0	448	448	0.26	60	19.33	12.71	5.17
(Easson et al., 2000)	1996	UK	Sandy clay loam	Maize	CK	0.89	448	0	448	448	0.2	60	14.83	12.71	5.17
(Easson et al., 2000)	1996	UK	Sandy clay loam	Maize	CK	0.24	448	0	448	448	0.05	60	4	12.71	5.17
(Easson et al., 2000)	1996	UK	Sandy clay loam	Maize	P	5.5	448	0	448	448	1.23	60	91.67	12.71	5.17
(Easson et al., 2000)	1996	UK	Sandy clay loam	Maize	P	4.31	448	0	448	448	0.96	60	71.83	12.71	5.17
(Easson et al., 2000)	1996	UK	Sandy clay loam	Maize	P	1.34	448	0	448	448	0.3	60	22.33	12.71	5.17
(Easson et al., 2000)	1996	UK	Sandy clay loam	Maize	CK	1.77	448	0	448	448	0.4	60	29.5	12.71	5.17
(Easson et al., 2000)	1996	UK	Sandy clay loam	Maize	CK	1.32	448	0	448	448	0.29	60	22	12.71	5.17
(Easson et al., 2000)	1996	UK	Sandy clay loam	Maize	CK	0.56	448	0	448	448	0.13	60	9.33	12.71	5.17
(Easson et al., 2000)	1996	UK	Sandy clay loam	Maize	P	5.71	448	0	448	448	1.27	60	95.17	12.71	5.17
(Easson et al., 2000)	1996	UK	Sandy clay loam	Maize	P	4.58	448	0	448	448	1.02	60	76.33	12.71	5.17
(Easson et al., 2000)	1996	UK	Sandy clay loam	Maize	P	2.53	448	0	448	448	0.56	60	42.17	12.71	5.17
(Easson et al., 2000)	1997	UK	Sandy clay loam	Maize	CK	5.61	489	0	489	489	1.15	60	93.5	12.71	5.17

(Easson et al., 2000)	1997	UK	Sandy clay loam	Maize	CK	2.39	489	0	489	489	0.49	60	39.83	12.71	5.17
(Easson et al., 2000)	1997	UK	Sandy clay loam	Maize	P	11.05	489	0	489	489	2.26	60	184.17	12.71	5.17
(Easson et al., 2000)	1997	UK	Sandy clay loam	Maize	P	7.43	489	0	489	489	1.52	60	123.83	12.71	5.17
(Easson et al., 2000)	1997	UK	Sandy clay loam	Maize	CK	8.21	489	0	489	489	1.68	60	136.83	12.71	5.17
(Easson et al., 2000)	1997	UK	Sandy clay loam	Maize	CK	4.07	489	0	489	489	0.83	60	67.83	12.71	5.17
(Easson et al., 2000)	1997	UK	Sandy clay loam	Maize	P	7.48	489	0	489	489	1.53	60	124.67	12.71	5.17
(Easson et al., 2000)	1997	UK	Sandy clay loam	Maize	P	6.85	489	0	489	489	1.4	60	114.17	12.71	5.17
(Easson et al., 2000)	1997	UK	Sandy clay loam	Maize	CK	6.6	489	0	489	489	1.35	60	110	12.71	5.17
(Easson et al., 2000)	1997	UK	Sandy clay loam	Maize	CK	5.46	489	0	489	489	1.12	60	91	12.71	5.17
(Easson et al., 2000)	1997	UK	Sandy clay loam	Maize	CK	3.13	489	0	489	489	0.64	60	52.17	12.71	5.17
(Easson et al., 2000)	1997	UK	Sandy clay loam	Maize	P	7.6	489	0	489	489	1.55	60	126.67	12.71	5.17
(Easson et al., 2000)	1997	UK	Sandy clay loam	Maize	P	9.18	489	0	489	489	1.88	60	153	12.71	5.17
(Easson et al., 2000)	1997	UK	Sandy clay loam	Maize	P	5.91	489	0	489	489	1.21	60	98.5	12.71	5.17
(Easson et al., 2000)	1997	UK	Sandy clay loam	Maize	P	7.53	489	0	489	489	1.54	60	125.5	12.71	5.17
(Easson et al., 2000)	1997	UK	Sandy clay loam	Maize	P	8.93	489	0	489	489	1.83	60	148.83	12.71	5.17
(Easson et al., 2000)	1997	UK	Sandy clay loam	Maize	P	7.07	489	0	489	489	1.45	60	117.83	12.71	5.17
(Easson et al., 2000)	1997	UK	Sandy clay loam	Maize	P	9.37	489	0	489	489	1.92	60	156.17	12.71	5.17
(Easson et al., 2000)	1997	UK	Sandy clay loam	Maize	P	9.24	489	0	489	489	1.89	60	154	12.71	5.17
(Easson et al., 2000)	1997	UK	Sandy clay loam	Maize	P	7.2	489	0	489	489	1.47	60	120	12.71	5.17
(Easson et al., 2000)	1997	UK	Sandy clay loam	Maize	P	8.07	489	0	489	489	1.65	60	134.5	12.71	5.17
(Easson et al., 2000)	1997	UK	Sandy clay loam	Maize	P	10.33	489	0	489	489	2.11	60	172.17	12.71	5.17
(Easson et al., 2000)	1997	UK	Sandy clay loam	Maize	P	6.69	489	0	489	489	1.37	60	111.5	12.71	5.17
(Easson et al., 2000)	1997	UK	Sandy clay loam	Maize	P	9.79	489	0	489	489	2	60	163.17	12.71	5.17
(Easson et al., 2000)	1997	UK	Sandy clay loam	Maize	P	9.51	489	0	489	489	1.94	60	158.5	12.71	5.17
(Easson et al., 2000)	1997	UK	Sandy clay loam	Maize	P	8.12	489	0	489	489	1.66	60	135.33	12.71	5.17
(Easson et al., 2000)	1997	UK	Sandy clay loam	Maize	P	7.56	489	0	489	489	1.55	60	126	12.71	5.17

(Easson et al., 2000)	1997	UK	Sandy clay loam	Maize	P	7.81	489	0	489	489	1.6	60	130.17	12.71	5.17
(Easson et al., 2000)	1997	UK	Sandy clay loam	Maize	P	7.85	489	0	489	489	1.61	60	130.83	12.71	5.17
(Gill et al., 1996)	1991	Ludhiana, India	Sandy loam	Maize	CK	0.5	352.98	225	577.98	544.18	0.09	150	3.33	26.16	0.34
(Gill et al., 1996)	1991	Ludhiana, India	Sandy loam	Maize	CK	1.6	352.98	375	727.98	544.18	0.29	150	10.67	26.16	0.34
(Gill et al., 1996)	1991	Ludhiana, India	Sandy loam	Maize	CK	2.4	352.98	225	577.98	544.18	0.44	150	16	26.16	0.34
(Gill et al., 1996)	1991	Ludhiana, India	Sandy loam	Maize	CK	3.6	352.98	375	727.98	544.18	0.66	150	24	26.16	0.34
(Gill et al., 1996)	1991	Ludhiana, India	Sandy loam	Maize	CK	0.8	225.13	150	375.13	493.47	0.16	150	5.33	26.16	0.34
(Gill et al., 1996)	1991	Ludhiana, India	Sandy loam	Maize	CK	1.6	225.13	225	450.13	493.47	0.32	150	10.67	26.16	0.34
(Gill et al., 1996)	1991	Ludhiana, India	Sandy loam	Maize	CK	2.4	225.13	150	375.13	493.47	0.49	150	16	26.16	0.34
(Gill et al., 1996)	1991	Ludhiana, India	Sandy loam	Maize	CK	2.9	225.13	225	450.13	493.47	0.59	150	19.33	26.16	0.34
(Gill et al., 1996)	1992	Ludhiana, India	Sandy loam	Maize	CK	3.1	468.25	225	693.25	669.04	0.46	150	20.67	26.16	0.34
(Gill et al., 1996)	1992	Ludhiana, India	Sandy loam	Maize	S	4.2	468.25	225	693.25	669.04	0.63	150	28	26.16	0.34
(Gill et al., 1996)	1992	Ludhiana, India	Sandy loam	Maize	CK	3.5	468.25	375	843.25	669.04	0.52	150	23.33	26.16	0.34
(Gill et al., 1996)	1992	Ludhiana, India	Sandy loam	Maize	S	3.5	468.25	375	843.25	669.04	0.52	150	23.33	26.16	0.34
(Gill et al., 1996)	1992	Ludhiana, India	Sandy loam	Maize	CK	4.5	468.25	225	693.25	669.04	0.67	150	30	26.16	0.34
(Gill et al., 1996)	1992	Ludhiana, India	Sandy loam	Maize	S	5.1	468.25	225	693.25	669.04	0.76	150	34	26.16	0.34
(Gill et al., 1996)	1992	Ludhiana, India	Sandy loam	Maize	CK	4.9	468.25	375	843.25	669.04	0.73	150	32.67	26.16	0.34
(Gill et al., 1996)	1992	Ludhiana, India	Sandy loam	Maize	S	4.6	468.25	375	843.25	669.04	0.69	150	30.67	26.16	0.34
(Gill et al., 1996)	1992	Ludhiana, India	Sandy loam	Maize	CK	3.6	496.48	0	496.48	450.25	0.8	150	24	26.16	0.34
(Gill et al., 1996)	1992	Ludhiana, India	Sandy loam	Maize	S	4.3	496.48	0	496.48	450.25	0.96	150	28.67	26.16	0.34
(Gill et al., 1996)	1992	Ludhiana, India	Sandy loam	Maize	CK	3.8	496.48	0	496.48	450.25	0.84	150	25.33	26.16	0.34
(Gill et al., 1996)	1992	Ludhiana, India	Sandy loam	Maize	S	4	496.48	0	496.48	450.25	0.89	150	26.67	26.16	0.34
(Gill et al., 1996)	1992	Ludhiana, India	Sandy loam	Maize	CK	4.6	496.48	0	496.48	450.25	1.02	150	30.67	26.16	0.34
(Gill et al., 1996)	1992	Ludhiana, India	Sandy loam	Maize	S	3.9	496.48	0	496.48	450.25	0.87	150	26	26.16	0.34
(Gill et al., 1996)	1992	Ludhiana, India	Sandy loam	Maize	CK	4.5	496.48	0	496.48	450.25	1	150	30	26.16	0.34
(Gill et al., 1996)	1992	Ludhiana, India	Sandy loam	Maize	S	4.3	496.48	0	496.48	450.25	0.96	150	28.67	26.16	0.34

(Gill et al., 1996)	1993	Ludhiana, India	Sandy loam	Maize	CK	0.2	653.82	300	953.82	645.06	0.03	150	1.33	26.16	0.34
(Gill et al., 1996)	1993	Ludhiana, India	Sandy loam	Maize	S	1.5	653.82	300	953.82	645.06	0.23	150	10	26.16	0.34
(Gill et al., 1996)	1993	Ludhiana, India	Sandy loam	Maize	CK	0.5	653.82	450	1103.82	645.06	0.08	150	3.33	26.16	0.34
(Gill et al., 1996)	1993	Ludhiana, India	Sandy loam	Maize	S	1	653.82	450	1103.82	645.06	0.16	150	6.67	26.16	0.34
(Gill et al., 1996)	1993	Ludhiana, India	Sandy loam	Maize	CK	1.1	653.82	300	953.82	645.06	0.17	150	7.33	26.16	0.34
(Gill et al., 1996)	1993	Ludhiana, India	Sandy loam	Maize	S	3.7	653.82	300	953.82	645.06	0.57	150	24.67	26.16	0.34
(Gill et al., 1996)	1993	Ludhiana, India	Sandy loam	Maize	CK	2.7	653.82	450	1103.82	645.06	0.42	150	18	26.16	0.34
(Gill et al., 1996)	1993	Ludhiana, India	Sandy loam	Maize	S	3.2	653.82	450	1103.82	645.06	0.5	150	21.33	26.16	0.34
(Gill et al., 1996)	1993	Ludhiana, India	Sandy loam	Maize	CK	0.1	732.33	150	882.33	451.68	0.02	150	0.67	26.16	0.34
(Gill et al., 1996)	1993	Ludhiana, India	Sandy loam	Maize	S	0.6	732.33	150	882.33	451.68	0.13	150	4	26.16	0.34
(Gill et al., 1996)	1993	Ludhiana, India	Sandy loam	Maize	CK	0.3	732.33	225	957.33	451.68	0.07	150	2	26.16	0.34
(Gill et al., 1996)	1993	Ludhiana, India	Sandy loam	Maize	S	0.6	732.33	225	957.33	451.68	0.13	150	4	26.16	0.34
(Gill et al., 1996)	1993	Ludhiana, India	Sandy loam	Maize	CK	0.4	732.33	150	882.33	451.68	0.09	150	2.67	26.16	0.34
(Gill et al., 1996)	1993	Ludhiana, India	Sandy loam	Maize	S	0.8	732.33	150	882.33	451.68	0.18	150	5.33	26.16	0.34
(Gill et al., 1996)	1993	Ludhiana, India	Sandy loam	Maize	CK	0.5	732.33	225	957.33	451.68	0.11	150	3.33	26.16	0.34
(Gill et al., 1996)	1993	Ludhiana, India	Sandy loam	Maize	S	0.7	732.33	225	957.33	451.68	0.15	150	4.67	26.16	0.34
(Fisher et al., 1995)	1988	Mexico	Sandy loam	Maize	CK	2.91	450.6	0	450.6	450.6	0.69	40	72.78	19.02	1
(Fisher et al., 1995)	1988	Mexico	Sandy loam	Maize	P	3.81	450.6	0	450.6	450.6	0.85	40	95.13	19.02	1
(Fisher et al., 1995)	1988	Mexico	Sandy loam	Maize	P	3.56	450.6	0	450.6	450.6	0.74	40	88.9	19.02	1
(Fisher et al., 1995)	1988	Mexico	Sandy loam	Maize	P	3.72	450.6	0	450.6	450.6	0.73	40	92.88	19.02	1
(Fisher et al., 1995)	1988	Mexico	Sandy loam	Maize	P	3.31	450.6	0	450.6	450.6	0.67	40	82.83	19.02	1
(Fisher et al., 1995)	1988	Mexico	Sandy loam	Maize	P	3.46	450.6	0	450.6	450.6	0.71	40	86.4	19.02	1
(Gajri et al., 1994)	1988	Ludhiana, India	Loamy sand	Maize	CK	1.4	600	150	750	700	0.2	150	9.33	26.16	0.34
(Gajri et al., 1994)	1988	Ludhiana, India	Loamy sand	Maize	CK	2.8	600	150	750	700	0.4	150	18.67	26.16	0.34
(Gajri et al., 1994)	1988	Ludhiana, India	Loamy sand	Maize	S	2.7	600	150	750	700	0.39	150	18	26.16	0.34
(Gajri et al., 1994)	1988	Ludhiana, India	Loamy sand	Maize	S	2.9	600	150	750	700	0.41	150	19.33	26.16	0.34

(Gajri et al., 1994)	1988	Ludhiana, India	Loamy sand	Maize	CK	2.3	600	150	750	700	0.33	247.5	9.29	26.16	0.34
(Gajri et al., 1994)	1988	Ludhiana, India	Loamy sand	Maize	CK	3.6	600	150	750	700	0.51	247.5	14.55	26.16	0.34
(Gajri et al., 1994)	1988	Ludhiana, India	Loamy sand	Maize	S	3.5	600	150	750	700	0.5	247.5	14.14	26.16	0.34
(Gajri et al., 1994)	1988	Ludhiana, India	Loamy sand	Maize	S	3.4	600	150	750	700	0.49	247.5	13.74	26.16	0.34
(Gajri et al., 1994)	1988	Ludhiana, India	Sandy loam	Maize	CK	5	600	150	750	700	0.71	150	33.33	26.16	0.34
(Gajri et al., 1994)	1988	Ludhiana, India	Sandy loam	Maize	CK	4.6	600	150	750	700	0.66	150	30.67	26.16	0.34
(Gajri et al., 1994)	1988	Ludhiana, India	Sandy loam	Maize	S	4.6	600	150	750	700	0.66	150	30.67	26.16	0.34
(Gajri et al., 1994)	1988	Ludhiana, India	Sandy loam	Maize	S	4.6	600	150	750	700	0.66	150	30.67	26.16	0.34
(Gajri et al., 1994)	1988	Ludhiana, India	Sandy loam	Maize	CK	4.6	600	150	750	700	0.66	247.5	18.59	26.16	0.34
(Gajri et al., 1994)	1988	Ludhiana, India	Sandy loam	Maize	CK	4.8	600	150	750	700	0.69	247.5	19.39	26.16	0.34
(Gajri et al., 1994)	1988	Ludhiana, India	Sandy loam	Maize	S	4.5	600	150	750	700	0.64	247.5	18.18	26.16	0.34
(Gajri et al., 1994)	1988	Ludhiana, India	Sandy loam	Maize	S	4.8	600	150	750	700	0.69	247.5	19.39	26.16	0.34
(Gajri et al., 1994)	1989	Ludhiana, India	Loamy sand	Maize	CK	0.5	300	150	450	450	0.11	150	3.33	26.16	0.34
(Gajri et al., 1994)	1989	Ludhiana, India	Loamy sand	Maize	CK	3.4	300	150	450	450	0.76	150	22.67	26.16	0.34
(Gajri et al., 1994)	1989	Ludhiana, India	Loamy sand	Maize	S	3.9	300	150	450	450	0.87	150	26	26.16	0.34
(Gajri et al., 1994)	1989	Ludhiana, India	Loamy sand	Maize	S	4.8	300	150	450	450	1.07	150	32	26.16	0.34
(Gajri et al., 1994)	1989	Ludhiana, India	Loamy sand	Maize	CK	1.3	300	150	450	450	0.29	247.5	5.25	26.16	0.34
(Gajri et al., 1994)	1989	Ludhiana, India	Loamy sand	Maize	CK	4.9	300	150	450	450	1.09	247.5	19.8	26.16	0.34
(Gajri et al., 1994)	1989	Ludhiana, India	Loamy sand	Maize	S	4.2	300	150	450	450	0.93	247.5	16.97	26.16	0.34
(Gajri et al., 1994)	1989	Ludhiana, India	Loamy sand	Maize	S	5.5	300	150	450	450	1.22	247.5	22.22	26.16	0.34
(Gajri et al., 1994)	1989	Ludhiana, India	Sandy loam	Maize	CK	3.8	300	150	450	450	0.84	150	25.33	26.16	0.34
(Gajri et al., 1994)	1989	Ludhiana, India	Sandy loam	Maize	CK	4.7	300	150	450	450	1.04	150	31.33	26.16	0.34
(Gajri et al., 1994)	1989	Ludhiana, India	Sandy loam	Maize	S	4.5	300	150	450	450	1	150	30	26.16	0.34
(Gajri et al., 1994)	1989	Ludhiana, India	Sandy loam	Maize	S	5.1	300	150	450	450	1.13	150	34	26.16	0.34
(Gajri et al., 1994)	1989	Ludhiana, India	Sandy loam	Maize	CK	4.9	300	150	450	450	1.09	247.5	19.8	26.16	0.34
(Gajri et al., 1994)	1989	Ludhiana, India	Sandy loam	Maize	CK	4.9	300	150	450	450	1.09	247.5	19.8	26.16	0.34

(Gajri et al., 1994)	1989	Ludhiana, India	Sandy loam	Maize	S	4.6	300	150	450	450	1.02	247.5	18.59	26.16	0.34
(Gajri et al., 1994)	1989	Ludhiana, India	Sandy loam	Maize	S	5.2	300	150	450	450	1.16	247.5	21.01	26.16	0.34
(Gajri et al., 1994)	1990	Ludhiana, India	Loamy sand	Maize	CK	1	1000	150	1150	700	0.14	150	6.67	26.16	0.34
(Gajri et al., 1994)	1990	Ludhiana, India	Loamy sand	Maize	CK	2.1	1000	150	1150	700	0.3	150	14	26.16	0.34
(Gajri et al., 1994)	1990	Ludhiana, India	Loamy sand	Maize	S	2	1000	150	1150	700	0.29	150	13.33	26.16	0.34
(Gajri et al., 1994)	1990	Ludhiana, India	Loamy sand	Maize	S	2.8	1000	150	1150	700	0.4	150	18.67	26.16	0.34
(Gajri et al., 1994)	1990	Ludhiana, India	Loamy sand	Maize	CK	1.9	1000	150	1150	700	0.27	247.5	7.68	26.16	0.34
(Gajri et al., 1994)	1990	Ludhiana, India	Loamy sand	Maize	CK	2.8	1000	150	1150	700	0.4	247.5	11.31	26.16	0.34
(Gajri et al., 1994)	1990	Ludhiana, India	Loamy sand	Maize	S	2.7	1000	150	1150	700	0.39	247.5	10.91	26.16	0.34
(Gajri et al., 1994)	1990	Ludhiana, India	Loamy sand	Maize	S	3.3	1000	150	1150	700	0.47	247.5	13.33	26.16	0.34
(Gajri et al., 1994)	1990	Ludhiana, India	Sandy loam	Maize	CK	2.7	1000	150	1150	700	0.39	150	18	26.16	0.34
(Gajri et al., 1994)	1990	Ludhiana, India	Sandy loam	Maize	CK	3.6	1000	150	1150	700	0.51	150	24	26.16	0.34
(Gajri et al., 1994)	1990	Ludhiana, India	Sandy loam	Maize	S	3.7	1000	150	1150	700	0.53	150	24.67	26.16	0.34
(Gajri et al., 1994)	1990	Ludhiana, India	Sandy loam	Maize	S	3.8	1000	150	1150	700	0.54	150	25.33	26.16	0.34
(Gajri et al., 1994)	1990	Ludhiana, India	Sandy loam	Maize	CK	3.4	1000	150	1150	700	0.49	247.5	13.74	26.16	0.34
(Gajri et al., 1994)	1990	Ludhiana, India	Sandy loam	Maize	CK	4.2	1000	150	1150	700	0.6	247.5	16.97	26.16	0.34
(Gajri et al., 1994)	1990	Ludhiana, India	Sandy loam	Maize	S	4.3	1000	150	1150	700	0.61	247.5	17.37	26.16	0.34
(Gajri et al., 1994)	1990	Ludhiana, India	Sandy loam	Maize	S	4.2	1000	150	1150	700	0.6	247.5	16.97	26.16	0.34
(Yibirin et al., 1993)	1986	Ohio, USA	Silt loam	Maize	CK	7.4	418	0	418	418	1.77	300	24.67	21.1	2.24
(Yibirin et al., 1993)	1986	Ohio, USA	Silt loam	Maize	CK	9.2	418	0	418	418	2.2	300	30.67	21.1	2.24
(Yibirin et al., 1993)	1986	Ohio, USA	Silt loam	Maize	CK	9.8	418	0	418	418	2.34	300	32.67	21.1	2.24
(Yibirin et al., 1993)	1986	Ohio, USA	Silt loam	Maize	CK	10.4	418	0	418	418	2.49	300	34.67	21.1	2.24
(Yibirin et al., 1993)	1986	Ohio, USA	Silt loam	Maize	CK	10.4	418	0	418	418	2.49	300	34.67	21.1	2.24
(Yibirin et al., 1993)	1986	Ohio, USA	Silt loam	Maize	CK	9	418	0	418	418	2.15	300	30	21.1	2.24
(Yibirin et al., 1993)	1986	Ohio, USA	Silt loam	Maize	CK	9.6	418	0	418	418	2.3	300	32	21.1	2.24
(Yibirin et al., 1993)	1986	Ohio, USA	Silt loam	Maize	CK	10.2	418	0	418	418	2.44	300	34	21.1	2.24

(Yibirin et al., 1993)	1986	Ohio, USA	Silt loam	Maize	CK	10.3	418	0	418	418	2.46	300	34.33	21.1	2.24
(Yibirin et al., 1993)	1986	Ohio, USA	Silt loam	Maize	CK	10.9	418	0	418	418	2.61	300	36.33	21.1	2.24
(Yibirin et al., 1993)	1986	Ohio, USA	Silt loam	Maize	S	9.8	418	0	418	418	2.34	300	32.67	21.1	2.24
(Yibirin et al., 1993)	1986	Ohio, USA	Silt loam	Maize	S	9.6	418	0	418	418	2.3	300	32	21.1	2.24
(Yibirin et al., 1993)	1986	Ohio, USA	Silt loam	Maize	S	10.1	418	0	418	418	2.42	300	33.67	21.1	2.24
(Yibirin et al., 1993)	1986	Ohio, USA	Silt loam	Maize	S	10.4	418	0	418	418	2.49	300	34.67	21.1	2.24
(Yibirin et al., 1993)	1986	Ohio, USA	Silt loam	Maize	S	10.6	418	0	418	418	2.54	300	35.33	21.1	2.24
(Yibirin et al., 1993)	1986	Ohio, USA	Silt loam	Maize	S	10.2	418	0	418	418	2.44	300	34	21.1	2.24
(Yibirin et al., 1993)	1986	Ohio, USA	Silt loam	Maize	S	9.9	418	0	418	418	2.37	300	33	21.1	2.24
(Yibirin et al., 1993)	1986	Ohio, USA	Silt loam	Maize	S	10.1	418	0	418	418	2.42	300	33.67	21.1	2.24
(Yibirin et al., 1993)	1986	Ohio, USA	Silt loam	Maize	S	10.5	418	0	418	418	2.51	300	35	21.1	2.24
(Yibirin et al., 1993)	1986	Ohio, USA	Silt loam	Maize	S	10.7	418	0	418	418	2.56	300	35.67	21.1	2.24
(Yibirin et al., 1993)	1986	Ohio, USA	Silt loam	Maize	S	9.6	418	0	418	418	2.3	300	32	21.1	2.24
(Yibirin et al., 1993)	1986	Ohio, USA	Silt loam	Maize	S	10	418	0	418	418	2.39	300	33.33	21.1	2.24
(Yibirin et al., 1993)	1986	Ohio, USA	Silt loam	Maize	S	9.9	418	0	418	418	2.37	300	33	21.1	2.24
(Yibirin et al., 1993)	1986	Ohio, USA	Silt loam	Maize	S	9.7	418	0	418	418	2.32	300	32.33	21.1	2.24
(Yibirin et al., 1993)	1986	Ohio, USA	Silt loam	Maize	S	9.9	418	0	418	418	2.37	300	33	21.1	2.24
(Yibirin et al., 1993)	1986	Ohio, USA	Silt loam	Maize	S	9.8	418	0	418	418	2.34	300	32.67	21.1	2.24
(Yibirin et al., 1993)	1986	Ohio, USA	Silt loam	Maize	S	9.6	418	0	418	418	2.3	300	32	21.1	2.24
(Yibirin et al., 1993)	1986	Ohio, USA	Silt loam	Maize	S	9.9	418	0	418	418	2.37	300	33	21.1	2.24
(Yibirin et al., 1993)	1986	Ohio, USA	Silt loam	Maize	S	9.6	418	0	418	418	2.3	300	32	21.1	2.24
(Yibirin et al., 1993)	1986	Ohio, USA	Silt loam	Maize	S	10	418	0	418	418	2.39	300	33.33	21.1	2.24
(Yibirin et al., 1993)	1988	Ohio, USA	Silt loam	Maize	CK	6.2	301	0	301	301	2.06	300	20.67	21.1	2.24
(Yibirin et al., 1993)	1988	Ohio, USA	Silt loam	Maize	CK	6.5	301	0	301	301	2.16	300	21.67	21.1	2.24
(Yibirin et al., 1993)	1988	Ohio, USA	Silt loam	Maize	CK	7	301	0	301	301	2.33	300	23.33	21.1	2.24
(Yibirin et al., 1993)	1988	Ohio, USA	Silt loam	Maize	CK	8.4	301	0	301	301	2.79	300	28	21.1	2.24

(Yibirin et al., 1993)	1988	Ohio, USA	Silt loam	Maize	CK	8.3	301	0	301	301	2.76	300	27.67	21.1	2.24
(Yibirin et al., 1993)	1988	Ohio, USA	Silt loam	Maize	CK	6	301	0	301	301	1.99	300	20	21.1	2.24
(Yibirin et al., 1993)	1988	Ohio, USA	Silt loam	Maize	CK	7.2	301	0	301	301	2.39	300	24	21.1	2.24
(Yibirin et al., 1993)	1988	Ohio, USA	Silt loam	Maize	CK	7.4	301	0	301	301	2.46	300	24.67	21.1	2.24
(Yibirin et al., 1993)	1988	Ohio, USA	Silt loam	Maize	CK	8.9	301	0	301	301	2.96	300	29.67	21.1	2.24
(Yibirin et al., 1993)	1988	Ohio, USA	Silt loam	Maize	CK	9.2	301	0	301	301	3.06	300	30.67	21.1	2.24
(Yibirin et al., 1993)	1988	Ohio, USA	Silt loam	Maize	S	8.6	301	0	301	301	2.86	300	28.67	21.1	2.24
(Yibirin et al., 1993)	1988	Ohio, USA	Silt loam	Maize	S	8.1	301	0	301	301	2.69	300	27	21.1	2.24
(Yibirin et al., 1993)	1988	Ohio, USA	Silt loam	Maize	S	8.6	301	0	301	301	2.86	300	28.67	21.1	2.24
(Yibirin et al., 1993)	1988	Ohio, USA	Silt loam	Maize	S	9.1	301	0	301	301	3.02	300	30.33	21.1	2.24
(Yibirin et al., 1993)	1988	Ohio, USA	Silt loam	Maize	S	9.6	301	0	301	301	3.19	300	32	21.1	2.24
(Yibirin et al., 1993)	1988	Ohio, USA	Silt loam	Maize	S	9.5	301	0	301	301	3.16	300	31.67	21.1	2.24
(Yibirin et al., 1993)	1988	Ohio, USA	Silt loam	Maize	S	8.5	301	0	301	301	2.82	300	28.33	21.1	2.24
(Yibirin et al., 1993)	1988	Ohio, USA	Silt loam	Maize	S	9.7	301	0	301	301	3.22	300	32.33	21.1	2.24
(Yibirin et al., 1993)	1988	Ohio, USA	Silt loam	Maize	S	10	301	0	301	301	3.32	300	33.33	21.1	2.24
(Yibirin et al., 1993)	1988	Ohio, USA	Silt loam	Maize	S	10.2	301	0	301	301	3.39	300	34	21.1	2.24
(Yibirin et al., 1993)	1988	Ohio, USA	Silt loam	Maize	S	9.4	301	0	301	301	3.12	300	31.33	21.1	2.24
(Yibirin et al., 1993)	1988	Ohio, USA	Silt loam	Maize	S	9.9	301	0	301	301	3.29	300	33	21.1	2.24
(Yibirin et al., 1993)	1988	Ohio, USA	Silt loam	Maize	S	9.6	301	0	301	301	3.19	300	32	21.1	2.24
(Yibirin et al., 1993)	1988	Ohio, USA	Silt loam	Maize	S	9.7	301	0	301	301	3.22	300	32.33	21.1	2.24
(Yibirin et al., 1993)	1988	Ohio, USA	Silt loam	Maize	S	9.7	301	0	301	301	3.22	300	32.33	21.1	2.24
(Yibirin et al., 1993)	1988	Ohio, USA	Silt loam	Maize	S	10	301	0	301	301	3.32	300	33.33	21.1	2.24
(Yibirin et al., 1993)	1988	Ohio, USA	Silt loam	Maize	S	9.6	301	0	301	301	3.19	300	32	21.1	2.24
(Yibirin et al., 1993)	1988	Ohio, USA	Silt loam	Maize	S	9.5	301	0	301	301	3.16	300	31.67	21.1	2.24
(Yibirin et al., 1993)	1988	Ohio, USA	Silt loam	Maize	S	9.5	301	0	301	301	3.16	300	31.67	21.1	2.24
(Yibirin et al., 1993)	1988	Ohio, USA	Silt loam	Maize	S	11.8	301	0	301	301	3.92	300	39.33	21.1	2.24

(Vanderwerf 1993)	1985	The Netherlands	Loam	Maize	P	9.5	413	0	413	413	2.3	200	47.5	14.88	3
(Vanderwerf 1993)	1985	The Netherlands	Sand	Maize	P	6.1	383	0	383	383	1.59	200	30.5	14.88	3
(Vanderwerf 1993)	1985	The Netherlands	Loam	Maize	CK	7.8	413	0	413	413	1.89	200	39	14.88	3
(Vanderwerf 1993)	1985	The Netherlands	Sand	Maize	CK	4.4	383	0	383	383	1.15	200	22	14.88	3
(Vanderwerf 1993)	1985	The Netherlands	Loam	Maize	P	8.5	413	0	413	413	2.06	200	42.5	14.88	3
(Vanderwerf 1993)	1985	The Netherlands	Sand	Maize	P	6.1	383	0	383	383	1.59	200	30.5	14.88	3
(Vanderwerf 1993)	1985	The Netherlands	Loam	Maize	CK	7.9	413	0	413	413	1.91	200	39.5	14.88	3
(Vanderwerf 1993)	1985	The Netherlands	Sand	Maize	CK	5.1	383	0	383	383	1.33	200	25.5	14.88	3
(Vanderwerf 1993)	1986	The Netherlands	Loam	Maize	P	9.7	318	0	318	318	3.05	200	48.5	14.88	3
(Vanderwerf 1993)	1986	The Netherlands	Sand	Maize	P	6.6	362	0	362	362	1.82	200	33	14.88	3
(Vanderwerf 1993)	1986	The Netherlands	Loam	Maize	CK	7.5	318	0	318	318	2.36	200	37.5	14.88	3
(Vanderwerf 1993)	1986	The Netherlands	Sand	Maize	CK	3.4	362	0	362	362	0.94	200	17	14.88	3
(Vanderwerf 1993)	1986	The Netherlands	Loam	Maize	P	9.2	318	0	318	318	2.89	200	46	14.88	3
(Vanderwerf 1993)	1986	The Netherlands	Sand	Maize	P	6.1	362	0	362	362	1.69	200	30.5	14.88	3
(Vanderwerf 1993)	1986	The Netherlands	Loam	Maize	CK	8	318	0	318	318	2.52	200	40	14.88	3
(Vanderwerf 1993)	1986	The Netherlands	Sand	Maize	CK	4.6	362	0	362	362	1.27	200	23	14.88	3
(Vanderwerf 1993)	1987	The Netherlands	Loam	Maize	P	7.3	761	0	761	761	0.96	200	36.5	14.88	3
(Vanderwerf 1993)	1987	The Netherlands	Loam	Maize	CK	5.4	761	0	761	761	0.71	200	27	14.88	3
(Vanderwerf 1993)	1987	The Netherlands	Loam	Maize	P	7.1	761	0	761	761	0.93	200	35.5	14.88	3
(Vanderwerf 1993)	1987	The Netherlands	Loam	Maize	CK	5.9	761	0	761	761	0.78	200	29.5	14.88	3
(Gill et al., 1992)	1989	Zambia	Clay	Maize	CK	5.35	853	0	853	853	0.63	110	48.6	20.52	2.28
(Gill et al., 1992)	1989	Zambia	Clay	Maize	S	5.56	853	0	853	853	0.65	110	50.57	20.52	2.28
(Gill et al., 1992)	1989	Zambia	Clay	Maize	S	6.41	853	0	853	853	0.75	110	58.3	20.52	2.28
(Gill et al., 1992)	1989	Zambia	Clay	Maize	S	6.21	853	0	853	853	0.73	110	56.45	20.52	2.28
(Gill et al., 1992)	1989	Zambia	Clay	Maize	S	6.83	853	0	853	853	0.8	110	62.12	20.52	2.28
(Gill et al., 1992)	1990	Zambia	Clay	Maize	CK	1.72	520	0	520	520	0.33	110	15.67	20.52	2.28

(Gill et al., 1992)	1990	Zambia	Clay	Maize	S	1.79	520	0	520	520	0.34	110	16.25	20.52	2.28
(Gill et al., 1992)	1990	Zambia	Clay	Maize	S	1.97	520	0	520	520	0.38	110	17.9	20.52	2.28
(Gill et al., 1992)	1990	Zambia	Clay	Maize	S	1.79	520	0	520	520	0.34	110	16.3	20.52	2.28
(Gill et al., 1992)	1990	Zambia	Clay	Maize	S	1.53	520	0	520	520	0.29	110	13.94	20.52	2.28
(Sharma et al., 1991)	1982	India	Sandy clay loam	Maize	CK	4.62	185	262	447	447	1.03	150	30.8	29.13	1.12
(Sharma et al., 1991)	1982	India	Sandy clay loam	Maize	S	5.65	202	316	518	518	1.09	150	37.67	29.13	1.12
(Sharma et al., 1991)	1982	India	Sandy clay loam	Maize	CK	5.33	203	321	524	524	1.02	150	35.53	29.13	1.12
(Sharma et al., 1991)	1982	India	Sandy clay loam	Maize	S	6.87	224	404	628	628	1.09	150	45.8	29.13	1.12
(Sharma et al., 1991)	1982	India	Sandy clay loam	Maize	CK	5.6	245	376	621	621	0.9	150	37.33	29.13	1.12
(Sharma et al., 1991)	1982	India	Sandy clay loam	Maize	S	7.36	257	453	710	710	1.04	150	49.07	29.13	1.12
(Sharma et al., 1991)	1983	India	Sandy clay loam	Maize	CK	4.64	263	233	496	496	0.94	150	30.93	29.13	1.12
(Sharma et al., 1991)	1983	India	Sandy clay loam	Maize	S	5.31	282	282	564	564	0.94	150	35.4	29.13	1.12
(Sharma et al., 1991)	1983	India	Sandy clay loam	Maize	CK	5.67	293	341	634	634	0.89	150	37.8	29.13	1.12
(Sharma et al., 1991)	1983	India	Sandy clay loam	Maize	S	7.06	325	374	699	699	1.01	150	47.07	29.13	1.12
(Sharma et al., 1991)	1983	India	Sandy clay loam	Maize	CK	5.8	318	403	721	721	0.8	150	38.67	29.13	1.12
(Sharma et al., 1991)	1983	India	Sandy clay loam	Maize	S	7.2	321	449	770	770	0.94	150	48	29.13	1.12
(Sandhu et al., 1986)	1982	Ludhiana, India	Sandy loam	Maize	CK	1.65	137	450	587	587	0.28	0	NA	24.15	0.52
(Sandhu et al., 1986)	1982	Ludhiana, India	Sandy loam	Maize	CK	2.05	137	450	587	587	0.35	30	68.2	24.15	0.52
(Sandhu et al., 1986)	1982	Ludhiana, India	Sandy loam	Maize	CK	2.89	137	450	587	587	0.49	60	48.13	24.15	0.52
(Sandhu et al., 1986)	1982	Ludhiana, India	Sandy loam	Maize	CK	3.25	6	750	756	756	0.43	0	NA	24.15	0.52
(Sandhu et al., 1986)	1982	Ludhiana, India	Sandy loam	Maize	CK	3.12	6	750	756	756	0.41	30	104.1	24.15	0.52
(Sandhu et al., 1986)	1982	Ludhiana, India	Sandy loam	Maize	CK	3.54	6	750	756	756	0.47	60	58.93	24.15	0.52
(Sandhu et al., 1986)	1982	Ludhiana, India	Sandy loam	Maize	S	1.52	137	450	587	587	0.26	0	NA	24.15	0.52
(Sandhu et al., 1986)	1982	Ludhiana, India	Sandy loam	Maize	S	2.28	137	450	587	587	0.39	30	76.13	24.15	0.52
(Sandhu et al., 1986)	1982	Ludhiana, India	Sandy loam	Maize	S	2.86	137	450	587	587	0.49	60	47.58	24.15	0.52
(Sandhu et al., 1986)	1982	Ludhiana, India	Sandy loam	Maize	S	3.57	6	750	756	756	0.47	0	NA	24.15	0.52

(Sandhu et al., 1986)	1982	Ludhiana, India	Sandy loam	Maize	S	3.53	6	750	756	756	0.47	30	117.67	24.15	0.52
(Sandhu et al., 1986)	1982	Ludhiana, India	Sandy loam	Maize	S	3.51	6	750	756	756	0.46	60	58.57	24.15	0.52
(Sandhu et al., 1986)	1983	Ludhiana, India	Sandy loam	Maize	CK	0.83	6	750	756	756	0.11	0	NA	24.15	0.52
(Sandhu et al., 1986)	1983	Ludhiana, India	Sandy loam	Maize	CK	1.84	6	750	756	756	0.24	30	61.37	24.15	0.52
(Sandhu et al., 1986)	1983	Ludhiana, India	Sandy loam	Maize	CK	2.18	6	750	756	756	0.29	60	36.4	24.15	0.52
(Sandhu et al., 1986)	1983	Ludhiana, India	Sandy loam	Maize	CK	2.79	4	750	754	754	0.37	0	NA	24.15	0.52
(Sandhu et al., 1986)	1983	Ludhiana, India	Sandy loam	Maize	CK	3.02	4	750	754	754	0.4	30	100.63	24.15	0.52
(Sandhu et al., 1986)	1983	Ludhiana, India	Sandy loam	Maize	CK	2.97	4	750	754	754	0.39	60	49.55	24.15	0.52
(Sandhu et al., 1986)	1983	Ludhiana, India	Sandy loam	Maize	S	1.18	6	750	756	756	0.16	0	NA	24.15	0.52
(Sandhu et al., 1986)	1983	Ludhiana, India	Sandy loam	Maize	S	2.04	6	750	756	756	0.27	30	67.93	24.15	0.52
(Sandhu et al., 1986)	1983	Ludhiana, India	Sandy loam	Maize	S	2.8	6	750	756	756	0.37	60	46.62	24.15	0.52
(Sandhu et al., 1986)	1983	Ludhiana, India	Sandy loam	Maize	S	3.48	4	750	754	754	0.46	0	NA	24.15	0.52
(Sandhu et al., 1986)	1983	Ludhiana, India	Sandy loam	Maize	S	3.69	4	750	754	754	0.49	30	123.03	24.15	0.52
(Sandhu et al., 1986)	1983	Ludhiana, India	Sandy loam	Maize	S	3.64	4	750	754	754	0.48	60	60.58	24.15	0.52
(Smika et al., 1971)	1963	Nebraska,USA	silt loam	Wheat	CK	2.01	212.9	0	212.9	212.9	0.94	0	NA	4.9	1.5
(Smika et al., 1971)	1963	Nebraska,USA	silt loam	Wheat	CK	2.22	212.9	0	212.9	212.9	1.04	168	13.21	4.9	1.5
(Smika et al., 1971)	1963	Nebraska,USA	silt loam	Wheat	S	2.22	212.9	0	212.9	212.9	1.04	0	NA	4.9	1.5
(Smika et al., 1971)	1963	Nebraska,USA	silt loam	Wheat	S	2.36	212.9	0	212.9	212.9	1.11	168	14.05	4.9	1.5
(Smika et al., 1971)	1964	Nebraska,USA	silt loam	Wheat	CK	2.21	291.1	0	291.1	291.1	0.76	0	NA	4.9	1.5
(Smika et al., 1971)	1964	Nebraska,USA	silt loam	Wheat	CK	2.25	291.1	0	291.1	291.1	0.77	168	13.39	4.9	1.5
(Smika et al., 1971)	1964	Nebraska,USA	silt loam	Wheat	S	1.83	291.1	0	291.1	291.1	0.63	0	NA	4.9	1.5
(Smika et al., 1971)	1964	Nebraska,USA	silt loam	Wheat	S	2	291.1	0	291.1	291.1	0.69	168	11.9	4.9	1.5
(Bakajev et al., 1981)	1968	Kazakhstan	Silty clay loam	Wheat	CK	1.55	116	0	116	278.4	0.56	60	25.83	18.25	3.65
(Bakajev et al., 1981)	1968	Kazakhstan	Silty clay loam	Wheat	S	1.56	116	0	116	301.4	0.52	60	26	18.25	3.65
(Bakajev et al., 1981)	1968	Kazakhstan	Silty clay loam	Wheat	S	1.57	116	0	116	302	0.52	60	26.17	18.25	3.65
(Bakajev et al., 1981)	1968	Kazakhstan	Silty clay loam	Wheat	S	1.63	116	0	116	228	0.71	60	27.17	18.25	3.65

(Bakajev et al., 1981)	1968	Kazakhstan	Silty clay loam	Wheat	S	1.8	116	0	116	228	0.79	60	30	18.25	3.65
(Bakajev et al., 1981)	1969	Kazakhstan	Silty clay loam	Wheat	CK	1.08	204	0	204	366.4	0.29	60	18	18.25	3.65
(Bakajev et al., 1981)	1969	Kazakhstan	Silty clay loam	Wheat	S	1.39	204	0	204	389.4	0.36	60	23.17	18.25	3.65
(Bakajev et al., 1981)	1969	Kazakhstan	Silty clay loam	Wheat	S	1.42	204	0	204	390	0.36	60	23.67	18.25	3.65
(Bakajev et al., 1981)	1969	Kazakhstan	Silty clay loam	Wheat	S	1.51	204	0	204	316	0.48	60	25.17	18.25	3.65
(Bakajev et al., 1981)	1969	Kazakhstan	Silty clay loam	Wheat	S	1.59	204	0	204	316	0.5	60	26.5	18.25	3.65
(Bakajev et al., 1981)	1970	Kazakhstan	Silty clay loam	Wheat	CK	1.32	114	0	114	276.4	0.48	60	22	18.25	3.65
(Bakajev et al., 1981)	1970	Kazakhstan	Silty clay loam	Wheat	S	1.44	114	0	114	299.4	0.48	60	24	18.25	3.65
(Bakajev et al., 1981)	1970	Kazakhstan	Silty clay loam	Wheat	S	1.49	114	0	114	300	0.5	60	24.83	18.25	3.65
(Bakajev et al., 1981)	1970	Kazakhstan	Silty clay loam	Wheat	S	1.54	114	0	114	226	0.68	60	25.67	18.25	3.65
(Bakajev et al., 1981)	1970	Kazakhstan	Silty clay loam	Wheat	S	1.49	114	0	114	226	0.66	60	24.83	18.25	3.65
(Bakajev et al., 1981)	1972	Kazakhstan	Silty clay loam	Wheat	CK	1.68	184	0	184	346.4	0.48	60	28	18.25	3.65
(Bakajev et al., 1981)	1972	Kazakhstan	Silty clay loam	Wheat	S	1.32	184	0	184	369.4	0.36	60	22	18.25	3.65
(Bakajev et al., 1981)	1972	Kazakhstan	Silty clay loam	Wheat	S	1.38	184	0	184	370	0.37	60	23	18.25	3.65
(Bakajev et al., 1981)	1972	Kazakhstan	Silty clay loam	Wheat	S	1.2	184	0	184	296	0.41	60	20	18.25	3.65
(Bakajev et al., 1981)	1972	Kazakhstan	Silty clay loam	Wheat	S	1	184	0	184	296	0.34	60	16.67	18.25	3.65
(Bakajev et al., 1981)	1973	Kazakhstan	Silty clay loam	Wheat	CK	1.23	117	0	117	279.4	0.44	60	20.5	18.25	3.65
(Bakajev et al., 1981)	1973	Kazakhstan	Silty clay loam	Wheat	S	1.35	117	0	117	302.4	0.45	60	22.5	18.25	3.65
(Bakajev et al., 1981)	1973	Kazakhstan	Silty clay loam	Wheat	S	1.35	117	0	117	303	0.45	60	22.5	18.25	3.65
(Bakajev et al., 1981)	1973	Kazakhstan	Silty clay loam	Wheat	S	1.34	117	0	117	229	0.59	60	22.33	18.25	3.65
(Bakajev et al., 1981)	1973	Kazakhstan	Silty clay loam	Wheat	S	0.93	117	0	117	229	0.41	60	15.5	18.25	3.65
(Bakajev et al., 1981)	1974	Kazakhstan	Silty clay loam	Wheat	CK	0.95	133	0	133	295.4	0.32	60	15.83	18.25	3.65
(Bakajev et al., 1981)	1974	Kazakhstan	Silty clay loam	Wheat	S	1.25	133	0	133	318.4	0.39	60	20.83	18.25	3.65
(Bakajev et al., 1981)	1974	Kazakhstan	Silty clay loam	Wheat	S	1.25	133	0	133	319	0.39	60	20.83	18.25	3.65
(Bakajev et al., 1981)	1974	Kazakhstan	Silty clay loam	Wheat	S	1.34	133	0	133	245	0.55	60	22.33	18.25	3.65
(Bakajev et al., 1981)	1974	Kazakhstan	Silty clay loam	Wheat	S	1.36	133	0	133	245	0.56	60	22.67	18.25	3.65

(Bakajev et al., 1981)	1976	Kazakhstan	Silty clay loam	Wheat	CK	1.55	86	0	86	248.4	0.62	60	25.83	18.25	3.65
(Bakajev et al., 1981)	1976	Kazakhstan	Silty clay loam	Wheat	S	1.65	86	0	86	271.4	0.61	60	27.5	18.25	3.65
(Bakajev et al., 1981)	1976	Kazakhstan	Silty clay loam	Wheat	S	1.68	86	0	86	272	0.62	60	28	18.25	3.65
(Bakajev et al., 1981)	1976	Kazakhstan	Silty clay loam	Wheat	S	1.83	86	0	86	198	0.92	60	30.5	18.25	3.65
(Bakajev et al., 1981)	1977	Kazakhstan	Silty clay loam	Wheat	CK	1.2	124	0	124	286.4	0.42	60	20	18.25	3.65
(Bakajev et al., 1981)	1977	Kazakhstan	Silty clay loam	Wheat	S	1.37	124	0	124	309.4	0.44	60	22.83	18.25	3.65
(Bakajev et al., 1981)	1977	Kazakhstan	Silty clay loam	Wheat	S	1.41	124	0	124	310	0.45	60	23.5	18.25	3.65
(Bakajev et al., 1981)	1977	Kazakhstan	Silty clay loam	Wheat	S	1.51	124	0	124	236	0.64	60	25.17	18.25	3.65
(Bakajev et al., 1981)	1978	Kazakhstan	Silty clay loam	Wheat	CK	2.15	85	0	85	247.4	0.87	60	35.83	18.25	3.65
(Bakajev et al., 1981)	1978	Kazakhstan	Silty clay loam	Wheat	S	2.32	85	0	85	270.4	0.86	60	38.67	18.25	3.65
(Bakajev et al., 1981)	1978	Kazakhstan	Silty clay loam	Wheat	S	2.33	85	0	85	271	0.86	60	38.83	18.25	3.65
(Bakajev et al., 1981)	1978	Kazakhstan	Silty clay loam	Wheat	S	2.55	85	0	85	197	1.29	60	42.5	18.25	3.65
(De et al., 1983)	1978	Hyderabad, India	Clay loam	Wheat	CK	2.04	22.1	50	72.1	176.6	1.16	120	17	23.03	1.72
(De et al., 1983)	1978	Hyderabad, India	Clay loam	Wheat	S	2.67	22.1	50	72.1	157.42	1.7	120	22.25	23.03	1.72
(De et al., 1983)	1978	Hyderabad, India	Clay loam	Wheat	CK	2.85	22.1	100	122.1	226.78	1.26	120	23.75	23.03	1.72
(De et al., 1983)	1978	Hyderabad, India	Clay loam	Wheat	S	3.49	22.1	100	122.1	180.34	1.94	120	29.08	23.03	1.72
(De et al., 1983)	1978	Hyderabad, India	Clay loam	Wheat	CK	3.38	22.1	150	172.1	265.74	1.27	120	28.17	23.03	1.72
(De et al., 1983)	1978	Hyderabad, India	Clay loam	Wheat	S	3.78	22.1	150	172.1	217.7	1.74	120	31.5	23.03	1.72
(De et al., 1983)	1978	Hyderabad, India	Clay loam	Wheat	CK	3.7	22.1	200	222.1	287.06	1.29	120	30.83	23.03	1.72
(De et al., 1983)	1978	Hyderabad, India	Clay loam	Wheat	S	4	22.1	200	222.1	256.66	1.56	120	33.33	23.03	1.72
(De et al., 1983)	1979	Hyderabad, India	Loamy sand	Wheat	CK	1.5	28.1	100	128.1	150.89	0.99	120	12.5	23.03	1.72
(De et al., 1983)	1979	Hyderabad, India	Loamy sand	Wheat	S	1.99	28.1	100	128.1	153.77	1.29	120	16.58	23.03	1.72
(De et al., 1983)	1979	Hyderabad, India	Loamy sand	Wheat	CK	1.74	28.1	200	228.1	221.66	0.78	120	14.5	23.03	1.72
(De et al., 1983)	1979	Hyderabad, India	Loamy sand	Wheat	S	2.13	28.1	200	228.1	225.83	0.94	120	17.75	23.03	1.72
(De et al., 1983)	1979	Hyderabad, India	Loamy sand	Wheat	CK	1.97	28.1	300	328.1	350.88	0.56	120	16.42	23.03	1.72
(De et al., 1983)	1979	Hyderabad, India	Loamy sand	Wheat	S	2.43	28.1	300	328.1	352.49	0.69	120	20.25	23.03	1.72

(Sharma et al., 1990)	1986	New Delhi, India	Sandy loam	Wheat	S	1.49	179	0	179	366.56	0.41	50	29.8	9.05	3.4
(Sharma et al., 1990)	1986	New Delhi, India	Sandy loam	Wheat	S	1.46	179	0	179	413.22	0.35	50	29.2	9.05	3.4
(Sharma et al., 1990)	1986	New Delhi, India	Sandy loam	Wheat	S	1.85	179	0	179	500.78	0.37	50	37	9.05	3.4
(Sharma et al., 1990)	1986	New Delhi, India	Sandy loam	Wheat	S	1.58	179	0	179	340.78	0.46	50	31.6	9.05	3.4
(Sharma et al., 1990)	1986	New Delhi, India	Sandy loam	Wheat	S	1.88	179	0	179	530.11	0.35	50	37.6	9.05	3.4
(Sharma et al., 1990)	1986	New Delhi, India	Sandy loam	Wheat	S	1.86	179	0	179	523	0.36	50	37.2	9.05	3.4
(Sharma et al., 1990)	1986	New Delhi, India	Sandy loam	Wheat	S	1.53	179	0	179	366.11	0.42	50	30.6	9.05	3.4
(Sharma et al., 1990)	1986	New Delhi, India	Sandy loam	Wheat	CK	1.22	179	0	179	229.22	0.53	50	24.4	9.05	3.4
(Sharma et al., 1990)	1987	New Delhi, India	Sandy loam	Wheat	S	0.68	124	0	124	333.33	0.2	50	13.6	9.05	3.4
(Sharma et al., 1990)	1987	New Delhi, India	Sandy loam	Wheat	S	0.58	124	0	124	325.78	0.18	50	11.6	9.05	3.4
(Sharma et al., 1990)	1987	New Delhi, India	Sandy loam	Wheat	S	0.65	124	0	124	354.67	0.18	50	13	9.05	3.4
(Sharma et al., 1990)	1987	New Delhi, India	Sandy loam	Wheat	S	0.49	124	0	124	257.78	0.19	50	9.8	9.05	3.4
(Sharma et al., 1990)	1987	New Delhi, India	Sandy loam	Wheat	S	0.64	124	0	124	319.11	0.2	50	12.8	9.05	3.4
(Sharma et al., 1990)	1987	New Delhi, India	Sandy loam	Wheat	S	0.74	124	0	124	322.22	0.23	50	14.8	9.05	3.4
(Sharma et al., 1990)	1987	New Delhi, India	Sandy loam	Wheat	S	0.52	124	0	124	270.22	0.19	50	10.4	9.05	3.4
(Sharma et al., 1990)	1987	New Delhi, India	Sandy loam	Wheat	CK	0.22	124	0	124	223.11	0.1	50	4.4	9.05	3.4
(Sandhu et al., 1992)	1980	New Delhi, India	Sandy loam	Wheat	CK	2.44	333	0	333	388	0.63	0	NA	15.94	0.55
(Sandhu et al., 1992)	1980	New Delhi, India	Sandy loam	Wheat	CK	3.1	333	0	333	388	0.8	40	77.43	15.94	0.55
(Sandhu et al., 1992)	1980	New Delhi, India	Sandy loam	Wheat	CK	3.49	333	0	333	388	0.9	60	58.1	15.94	0.55
(Sandhu et al., 1992)	1980	New Delhi, India	Sandy loam	Wheat	CK	3.88	333	0	333	388	1	80	48.44	15.94	0.55
(Sandhu et al., 1992)	1980	New Delhi, India	Sandy loam	Wheat	S	3.19	333	0	333	343	0.93	0	NA	15.94	0.55
(Sandhu et al., 1992)	1980	New Delhi, India	Sandy loam	Wheat	S	3.58	333	0	333	343	1.04	40	89.58	15.94	0.55
(Sandhu et al., 1992)	1980	New Delhi, India	Sandy loam	Wheat	S	3.81	333	0	333	343	1.11	60	63.43	15.94	0.55
(Sandhu et al., 1992)	1980	New Delhi, India	Sandy loam	Wheat	S	3.92	333	0	333	343	1.14	80	48.96	15.94	0.55
(Sandhu et al., 1992)	1981	New Delhi, India	Sandy loam	Wheat	CK	2.11	409	0	409	461	0.46	0	NA	15.94	0.55
(Sandhu et al., 1992)	1981	New Delhi, India	Sandy loam	Wheat	CK	2.59	409	0	409	461	0.56	20	129.5	15.94	0.55

(Sandhu et al., 1992)	1981	New Delhi, India	Sandy loam	Wheat	CK	2.77	409	0	409	461	0.6	40	69.25	15.94	0.55
(Sandhu et al., 1992)	1981	New Delhi, India	Sandy loam	Wheat	CK	3.16	409	0	409	461	0.69	60	52.73	15.94	0.55
(Sandhu et al., 1992)	1981	New Delhi, India	Sandy loam	Wheat	S	2.39	409	0	409	452	0.53	0	NA	15.94	0.55
(Sandhu et al., 1992)	1981	New Delhi, India	Sandy loam	Wheat	S	2.7	409	0	409	452	0.6	20	135.05	15.94	0.55
(Sandhu et al., 1992)	1981	New Delhi, India	Sandy loam	Wheat	S	3.26	409	0	409	452	0.72	40	81.45	15.94	0.55
(Sandhu et al., 1992)	1981	New Delhi, India	Sandy loam	Wheat	S	3.41	409	0	409	452	0.75	60	56.85	15.94	0.55
(Sandhu et al., 1992)	1981	New Delhi, India	Sandy loam	Wheat	S	3.46	409	0	409	395	0.88	0	NA	15.94	0.55
(Sandhu et al., 1992)	1981	New Delhi, India	Sandy loam	Wheat	S	3.33	409	0	409	395	0.84	20	166.6	15.94	0.55
(Sandhu et al., 1992)	1981	New Delhi, India	Sandy loam	Wheat	S	3.81	409	0	409	395	0.96	40	95.28	15.94	0.55
(Sandhu et al., 1992)	1981	New Delhi, India	Sandy loam	Wheat	S	4.16	409	0	409	395	1.05	60	69.38	15.94	0.55
(Sandhu et al., 1992)	1982	New Delhi, India	Sandy loam	Wheat	CK	2.33	334	0	334	319	0.73	0	NA	15.94	0.55
(Sandhu et al., 1992)	1982	New Delhi, India	Sandy loam	Wheat	CK	2.44	334	0	334	319	0.76	40	60.93	15.94	0.55
(Sandhu et al., 1992)	1982	New Delhi, India	Sandy loam	Wheat	CK	2.79	334	0	334	319	0.87	60	46.43	15.94	0.55
(Sandhu et al., 1992)	1982	New Delhi, India	Sandy loam	Wheat	CK	2.96	334	0	334	319	0.93	80	37	15.94	0.55
(Sandhu et al., 1992)	1982	New Delhi, India	Sandy loam	Wheat	S	3.06	334	0	334	322	0.95	0	NA	15.94	0.55
(Sandhu et al., 1992)	1982	New Delhi, India	Sandy loam	Wheat	S	3.33	334	0	334	322	1.03	40	83.13	15.94	0.55
(Sandhu et al., 1992)	1982	New Delhi, India	Sandy loam	Wheat	S	3.46	334	0	334	322	1.07	60	57.67	15.94	0.55
(Sandhu et al., 1992)	1982	New Delhi, India	Sandy loam	Wheat	S	3.36	334	0	334	322	1.04	80	41.96	15.94	0.55
(Sandhu et al., 1992)	1983	New Delhi, India	Sandy loam	Wheat	CK	1.87	142	0	142	323	0.58	0	NA	15.94	0.55
(Sandhu et al., 1992)	1983	New Delhi, India	Sandy loam	Wheat	CK	2.08	142	0	142	323	0.64	40	52.08	15.94	0.55
(Sandhu et al., 1992)	1983	New Delhi, India	Sandy loam	Wheat	CK	2.37	142	0	142	323	0.73	60	39.52	15.94	0.55
(Sandhu et al., 1992)	1983	New Delhi, India	Sandy loam	Wheat	CK	2.89	142	0	142	323	0.89	80	36.09	15.94	0.55
(Sandhu et al., 1992)	1983	New Delhi, India	Sandy loam	Wheat	S	1.88	142	0	142	325	0.58	0	NA	15.94	0.55
(Sandhu et al., 1992)	1983	New Delhi, India	Sandy loam	Wheat	S	2.09	142	0	142	325	0.64	40	52.33	15.94	0.55
(Sandhu et al., 1992)	1983	New Delhi, India	Sandy loam	Wheat	S	2.39	142	0	142	325	0.74	60	39.85	15.94	0.55
(Sandhu et al., 1992)	1983	New Delhi, India	Sandy loam	Wheat	S	2.89	142	0	142	325	0.89	80	36.13	15.94	0.55

(Sandhu et al., 1992)	1983	New Delhi, India	Sandy loam	Wheat	S	2.66	142	0	142	324	0.82	0	NA	15.94	0.55
(Sandhu et al., 1992)	1983	New Delhi, India	Sandy loam	Wheat	S	2.88	142	0	142	324	0.89	40	71.93	15.94	0.55
(Sandhu et al., 1992)	1983	New Delhi, India	Sandy loam	Wheat	S	3.34	142	0	142	324	1.03	60	55.63	15.94	0.55
(Sandhu et al., 1992)	1983	New Delhi, India	Sandy loam	Wheat	S	3.56	142	0	142	324	1.1	80	44.53	15.94	0.55
(Sandhu et al., 1992)	1984	New Delhi, India	Sandy loam	Wheat	CK	1.71	24	0	24	203	0.84	0	NA	15.94	0.55
(Sandhu et al., 1992)	1984	New Delhi, India	Sandy loam	Wheat	CK	2.17	24	0	24	203	1.07	40	54.15	15.94	0.55
(Sandhu et al., 1992)	1984	New Delhi, India	Sandy loam	Wheat	CK	2.21	24	0	24	203	1.09	60	36.87	15.94	0.55
(Sandhu et al., 1992)	1984	New Delhi, India	Sandy loam	Wheat	CK	2.38	24	0	24	203	1.17	80	29.75	15.94	0.55
(Sandhu et al., 1992)	1984	New Delhi, India	Sandy loam	Wheat	S	2.21	24	0	24	203	1.09	0	NA	15.94	0.55
(Sandhu et al., 1992)	1984	New Delhi, India	Sandy loam	Wheat	S	2.76	24	0	24	203	1.36	40	69.08	15.94	0.55
(Sandhu et al., 1992)	1984	New Delhi, India	Sandy loam	Wheat	S	2.89	24	0	24	203	1.42	60	48.15	15.94	0.55
(Sandhu et al., 1992)	1984	New Delhi, India	Sandy loam	Wheat	S	2.67	24	0	24	203	1.31	80	33.31	15.94	0.55
(Sandhu et al., 1992)	1984	New Delhi, India	Sandy loam	Wheat	S	2.61	24	0	24	205	1.27	0	NA	15.94	0.55
(Sandhu et al., 1992)	1984	New Delhi, India	Sandy loam	Wheat	S	3.05	24	0	24	205	1.49	40	76.2	15.94	0.55
(Sandhu et al., 1992)	1984	New Delhi, India	Sandy loam	Wheat	S	3.19	24	0	24	205	1.56	60	53.22	15.94	0.55
(Sandhu et al., 1992)	1984	New Delhi, India	Sandy loam	Wheat	S	3.14	24	0	24	205	1.53	80	39.21	15.94	0.55
(Sandhu et al., 1992)	1985	New Delhi, India	Sandy loam	Wheat	CK	2.47	143	0	143	326	0.76	0	NA	15.94	0.55
(Sandhu et al., 1992)	1985	New Delhi, India	Sandy loam	Wheat	CK	3.35	143	0	143	326	1.03	40	83.65	15.94	0.55
(Sandhu et al., 1992)	1985	New Delhi, India	Sandy loam	Wheat	CK	3.62	143	0	143	326	1.11	60	60.25	15.94	0.55
(Sandhu et al., 1992)	1985	New Delhi, India	Sandy loam	Wheat	CK	3.4	143	0	143	326	1.04	80	42.53	15.94	0.55
(Sandhu et al., 1992)	1985	New Delhi, India	Sandy loam	Wheat	S	2.73	143	0	143	325	0.84	0	NA	15.94	0.55
(Sandhu et al., 1992)	1985	New Delhi, India	Sandy loam	Wheat	S	3.62	143	0	143	325	1.12	40	90.6	15.94	0.55
(Sandhu et al., 1992)	1985	New Delhi, India	Sandy loam	Wheat	S	3.54	143	0	143	325	1.09	60	59.02	15.94	0.55
(Sandhu et al., 1992)	1985	New Delhi, India	Sandy loam	Wheat	S	3.42	143	0	143	325	1.05	80	42.78	15.94	0.55
(Sandhu et al., 1992)	1985	New Delhi, India	Sandy loam	Wheat	S	3.38	143	0	143	326	1.04	0	NA	15.94	0.55
(Sandhu et al., 1992)	1985	New Delhi, India	Sandy loam	Wheat	S	3.79	143	0	143	326	1.16	40	94.7	15.94	0.55

(Sandhu et al., 1992)	1985	New Delhi, India	Sandy loam	Wheat	S	4.14	143	0	143	326	1.27	60	68.92	15.94	0.55
(Sandhu et al., 1992)	1985	New Delhi, India	Sandy loam	Wheat	S	4.57	143	0	143	326	1.4	80	57.16	15.94	0.55
(Acharya et al., 1994)	1980	Himachal Pradesh, India	Silty clay loam	Wheat	CK	2.7	595	0	595	480.7	0.56	120	22.5	15.04	1.29
(Acharya et al., 1994)	1980	Himachal Pradesh, India	Silty clay loam	Wheat	S	3.46	595	0	595	480.7	0.72	120	28.86	15.04	1.29
(Acharya et al., 1994)	1980	Himachal Pradesh, India	Silty clay loam	Wheat	CK	2.58	595	0	595	480.7	0.54	120	21.46	15.04	1.29
(Acharya et al., 1994)	1980	Himachal Pradesh, India	Silty clay loam	Wheat	CK	3.05	595	0	595	480.7	0.63	120	25.42	15.04	1.29
(Acharya et al., 1994)	1981	Himachal Pradesh, India	Silty clay loam	Wheat	CK	2.3	1017	0	1017	511.2	0.45	120	19.17	15.04	1.29
(Acharya et al., 1994)	1981	Himachal Pradesh, India	Silty clay loam	Wheat	S	3.46	1017	0	1017	511.2	0.68	120	28.86	15.04	1.29
(Acharya et al., 1994)	1981	Himachal Pradesh, India	Silty clay loam	Wheat	CK	1.95	1017	0	1017	511.2	0.38	120	16.25	15.04	1.29
(Acharya et al., 1994)	1981	Himachal Pradesh, India	Silty clay loam	Wheat	CK	3.23	1017	0	1017	511.2	0.63	120	26.88	15.04	1.29
(Acharya et al., 1994)	1982	Himachal Pradesh, India	Silty clay loam	Wheat	CK	2.17	592	0	592	585.2	0.37	120	18.1	15.04	1.29
(Acharya et al., 1994)	1982	Himachal Pradesh, India	Silty clay loam	Wheat	S	2.72	592	0	592	585.2	0.46	120	22.68	15.04	1.29
(Acharya et al., 1994)	1982	Himachal Pradesh, India	Silty clay loam	Wheat	CK	2.08	592	0	592	585.2	0.36	120	17.33	15.04	1.29
(Acharya et al., 1994)	1982	Himachal Pradesh, India	Silty clay loam	Wheat	S	2.41	592	0	592	585.2	0.41	120	20.12	15.04	1.29
(Acharya et al., 1994)	1982	Himachal Pradesh, India	Silty clay loam	Wheat	CK	2.63	592	0	592	585.2	0.45	120	21.91	15.04	1.29
(Acharya et al., 1994)	1983	Himachal Pradesh, India	Silty clay loam	Wheat	CK	2.38	239	0	239	339	0.7	120	19.87	15.04	1.29
(Acharya et al., 1994)	1983	Himachal Pradesh, India	Silty clay loam	Wheat	S	2.97	239	0	239	339	0.87	120	24.71	15.04	1.29
(Acharya et al., 1994)	1983	Himachal Pradesh, India	Silty clay loam	Wheat	CK	2.16	239	0	239	339	0.64	120	18.03	15.04	1.29
(Acharya et al., 1994)	1983	Himachal Pradesh, India	Silty clay loam	Wheat	S	2.74	239	0	239	339	0.81	120	22.83	15.04	1.29
(Acharya et al., 1994)	1983	Himachal Pradesh, India	Silty clay loam	Wheat	CK	2.31	239	0	239	339	0.68	120	19.22	15.04	1.29
(Acharya et al., 1994)	1984	Himachal Pradesh, India	Silty clay loam	Wheat	CK	1.69	346	0	346	446	0.38	120	14.05	15.04	1.29
(Acharya et al., 1994)	1984	Himachal Pradesh, India	Silty clay loam	Wheat	S	2.14	346	0	346	446	0.48	120	17.81	15.04	1.29
(Acharya et al., 1994)	1984	Himachal Pradesh, India	Silty clay loam	Wheat	CK	1.34	346	0	346	446	0.3	120	11.19	15.04	1.29
(Acharya et al., 1994)	1984	Himachal Pradesh, India	Silty clay loam	Wheat	S	2.25	346	0	346	446	0.5	120	18.72	15.04	1.29
(Acharya et al., 1994)	1984	Himachal Pradesh, India	Silty clay loam	Wheat	CK	1.93	346	0	346	446	0.43	120	16.05	15.04	1.29
(Acharya et al., 1994)	1985	Himachal Pradesh, India	Silty clay loam	Wheat	CK	1.55	426	0	426	526	0.29	120	12.91	15.04	1.29

(Acharya et al., 1994)	1985	Himachal Pradesh, India	Silty clay loam	Wheat	S	1.92	426	0	426	526	0.37	120	16.02	15.04	1.29
(Acharya et al., 1994)	1985	Himachal Pradesh, India	Silty clay loam	Wheat	CK	1.48	426	0	426	526	0.28	120	12.29	15.04	1.29
(Acharya et al., 1994)	1985	Himachal Pradesh, India	Silty clay loam	Wheat	S	2.11	426	0	426	526	0.4	120	17.62	15.04	1.29
(Acharya et al., 1994)	1985	Himachal Pradesh, India	Silty clay loam	Wheat	CK	1.56	426	0	426	526	0.3	120	13	15.04	1.29
(Yunusa et al., 1994)	1988	Merredin,Au	Sandy loam	Wheat	CK	2.05	181	0	181	202.87	1.01	60	34.15	10	1.59
(Yunusa et al., 1994)	1988	Merredin,Au	Sandy loam	Wheat	S	2.16	181	0	181	184.87	1.17	60	36.05	10	1.59
(Acharya et al., 1998)	1989	Himachal Pradesh, India	Silty clay loam	Wheat	S	2.81	428.7	0	428.7	428.7	0.66	60	46.83	16.41	1.38
(Acharya et al., 1998)	1989	Himachal Pradesh, India	Silty clay loam	Wheat	S	3.27	428.7	0	428.7	428.7	0.76	120	27.26	16.41	1.38
(Acharya et al., 1998)	1989	Himachal Pradesh, India	Silty clay loam	Wheat	S	3.1	428.7	0	428.7	428.7	0.72	60	51.7	16.41	1.38
(Acharya et al., 1998)	1989	Himachal Pradesh, India	Silty clay loam	Wheat	S	3.38	428.7	0	428.7	428.7	0.79	120	28.18	16.41	1.38
(Acharya et al., 1998)	1989	Himachal Pradesh, India	Silty clay loam	Wheat	S	2.63	428.7	0	428.7	428.7	0.61	60	43.83	16.41	1.38
(Acharya et al., 1998)	1989	Himachal Pradesh, India	Silty clay loam	Wheat	S	3.04	428.7	0	428.7	428.7	0.71	120	25.36	16.41	1.38
(Acharya et al., 1998)	1989	Himachal Pradesh, India	Silty clay loam	Wheat	S	2.84	428.7	0	428.7	428.7	0.66	60	47.35	16.41	1.38
(Acharya et al., 1998)	1989	Himachal Pradesh, India	Silty clay loam	Wheat	S	3.14	428.7	0	428.7	428.7	0.73	120	26.19	16.41	1.38
(Acharya et al., 1998)	1989	Himachal Pradesh, India	Silty clay loam	Wheat	CK	1.53	428.7	0	428.7	428.7	0.36	60	25.52	16.41	1.38
(Acharya et al., 1998)	1989	Himachal Pradesh, India	Silty clay loam	Wheat	CK	1.83	428.7	0	428.7	428.7	0.43	120	15.25	16.41	1.38
(Acharya et al., 1998)	1990	Himachal Pradesh, India	Silty clay loam	Wheat	S	3.49	416.2	0	416.2	416.2	0.84	60	58.2	16.41	1.38
(Acharya et al., 1998)	1990	Himachal Pradesh, India	Silty clay loam	Wheat	S	4.29	416.2	0	416.2	416.2	1.03	120	35.76	16.41	1.38
(Acharya et al., 1998)	1990	Himachal Pradesh, India	Silty clay loam	Wheat	S	4.12	416.2	0	416.2	416.2	0.99	60	68.72	16.41	1.38
(Acharya et al., 1998)	1990	Himachal Pradesh, India	Silty clay loam	Wheat	S	4.27	416.2	0	416.2	416.2	1.03	120	35.6	16.41	1.38
(Acharya et al., 1998)	1990	Himachal Pradesh, India	Silty clay loam	Wheat	S	3.5	416.2	0	416.2	416.2	0.84	60	58.35	16.41	1.38
(Acharya et al., 1998)	1990	Himachal Pradesh, India	Silty clay loam	Wheat	S	4.02	416.2	0	416.2	416.2	0.97	120	33.51	16.41	1.38
(Acharya et al., 1998)	1990	Himachal Pradesh, India	Silty clay loam	Wheat	S	4.05	416.2	0	416.2	416.2	0.97	60	67.5	16.41	1.38
(Acharya et al., 1998)	1990	Himachal Pradesh, India	Silty clay loam	Wheat	S	4.29	416.2	0	416.2	416.2	1.03	120	35.77	16.41	1.38
(Acharya et al., 1998)	1990	Himachal Pradesh, India	Silty clay loam	Wheat	CK	2.23	416.2	0	416.2	416.2	0.54	60	37.18	16.41	1.38
(Acharya et al., 1998)	1990	Himachal Pradesh, India	Silty clay loam	Wheat	CK	2.27	416.2	0	416.2	416.2	0.55	120	18.92	16.41	1.38

(Acharya et al., 1998)	1991	Himachal Pradesh, India	Silty clay loam	Wheat	S	2.27	440.4	0	440.4	440.4	0.52	60	37.85	16.41	1.38
(Acharya et al., 1998)	1991	Himachal Pradesh, India	Silty clay loam	Wheat	S	2.71	440.4	0	440.4	440.4	0.62	120	22.59	16.41	1.38
(Acharya et al., 1998)	1991	Himachal Pradesh, India	Silty clay loam	Wheat	S	2.53	440.4	0	440.4	440.4	0.57	60	42.2	16.41	1.38
(Acharya et al., 1998)	1991	Himachal Pradesh, India	Silty clay loam	Wheat	S	2.94	440.4	0	440.4	440.4	0.67	120	24.52	16.41	1.38
(Acharya et al., 1998)	1991	Himachal Pradesh, India	Silty clay loam	Wheat	S	1.85	440.4	0	440.4	440.4	0.42	60	30.83	16.41	1.38
(Acharya et al., 1998)	1991	Himachal Pradesh, India	Silty clay loam	Wheat	S	2.33	440.4	0	440.4	440.4	0.53	120	19.42	16.41	1.38
(Acharya et al., 1998)	1991	Himachal Pradesh, India	Silty clay loam	Wheat	S	2.25	440.4	0	440.4	440.4	0.51	60	37.53	16.41	1.38
(Acharya et al., 1998)	1991	Himachal Pradesh, India	Silty clay loam	Wheat	S	2.62	440.4	0	440.4	440.4	0.6	120	21.86	16.41	1.38
(Acharya et al., 1998)	1991	Himachal Pradesh, India	Silty clay loam	Wheat	CK	1.23	440.4	0	440.4	440.4	0.28	60	20.52	16.41	1.38
(Acharya et al., 1998)	1991	Himachal Pradesh, India	Silty clay loam	Wheat	CK	1.28	440.4	0	440.4	440.4	0.29	120	10.68	16.41	1.38
(Niu et al., 1998)	1995	Lanzhou, China	Sandy loam	Wheat	P	4.56	135	300	435	435	1.05	237	19.22	16.05	1.22
(Niu et al., 1998)	1995	Lanzhou, China	Sandy loam	Wheat	CK	3.35	135	300	435	435	0.77	237	14.12	16.05	1.22
(Niu et al., 1998)	1996	Lanzhou, China	Sandy loam	Wheat	P	4.89	135	375	510	510	0.96	237	20.62	16.05	1.22
(Niu et al., 1998)	1996	Lanzhou, China	Sandy loam	Wheat	CK	3.57	135	375	510	510	0.7	237	15.07	16.05	1.22
(Sharma et al., 1998)	1990	Dehradun, India	Silty clay loam	Wheat	CK	3.06	274.9	0	274.9	329.1	0.93	60	50.98	18.21	1.02
(Sharma et al., 1998)	1990	Dehradun, India	Silty clay loam	Wheat	S	3.73	274.9	0	274.9	360.8	1.03	60	62.1	18.21	1.02
(Sharma et al., 1998)	1990	Dehradun, India	Silty clay loam	Wheat	S	3.87	274.9	0	274.9	358.9	1.08	60	64.52	18.21	1.02
(Sharma et al., 1998)	1990	Dehradun, India	Silty clay loam	Wheat	S	4.22	274.9	0	274.9	367.9	1.15	60	70.25	18.21	1.02
(Sharma et al., 1998)	1991	Dehradun, India	Silty clay loam	Wheat	CK	2.42	166.7	0	166.7	244.3	0.99	60	40.35	18.21	1.02
(Sharma et al., 1998)	1991	Dehradun, India	Silty clay loam	Wheat	S	2.92	166.7	0	166.7	248.4	1.18	60	48.73	18.21	1.02
(Sharma et al., 1998)	1991	Dehradun, India	Silty clay loam	Wheat	S	3.06	166.7	0	166.7	246.9	1.24	60	51.05	18.21	1.02
(Sharma et al., 1998)	1991	Dehradun, India	Silty clay loam	Wheat	S	3.16	166.7	0	166.7	250.7	1.26	60	52.68	18.21	1.02
(Sharma et al., 1998)	1992	Dehradun, India	Silty clay loam	Wheat	CK	2.34	195	0	195	241	0.97	60	39.05	18.21	1.02
(Sharma et al., 1998)	1992	Dehradun, India	Silty clay loam	Wheat	S	2.97	195	0	195	242.4	1.23	60	49.53	18.21	1.02
(Sharma et al., 1998)	1992	Dehradun, India	Silty clay loam	Wheat	S	3.37	195	0	195	259.5	1.3	60	56.2	18.21	1.02
(Sharma et al., 1998)	1992	Dehradun, India	Silty clay loam	Wheat	S	3.5	195	0	195	265.7	1.32	60	58.25	18.21	1.02

(Badaruddin et al., 1999)	1990	Dinajpur, Bangladesh	Silt loam	Wheat	CK	2.5	53	425	478	478	0.52	120	20.83	23.3	0.91
(Badaruddin et al., 1999)	1990	Dinajpur, Bangladesh	Silt loam	Wheat	CK	2.9	53	425	478	478	0.61	180	16.11	23.3	0.91
(Badaruddin et al., 1999)	1990	Dinajpur, Bangladesh	Silt loam	Wheat	S	3.1	53	425	478	478	0.65	220	14.09	23.3	0.91
(Badaruddin et al., 1999)	1990	Dinajpur, Bangladesh	Silt loam	Wheat	S	2.7	53	425	478	478	0.56	120	22.5	23.3	0.91
(Badaruddin et al., 1999)	1991	Dinajpur, Bangladesh	Silt loam	Wheat	CK	2.31	53	400	453	453	0.51	120	19.27	23.3	0.91
(Badaruddin et al., 1999)	1991	Dinajpur, Bangladesh	Silt loam	Wheat	S	2.82	53	400	453	453	0.62	220	12.81	23.3	0.91
(Badaruddin et al., 1999)	1991	Dinajpur, Bangladesh	Silt loam	Wheat	CK	2.9	53	400	453	453	0.64	180	16.11	23.3	0.91
(Badaruddin et al., 1999)	1991	Dinajpur, Bangladesh	Silt loam	Wheat	CK	2.75	53	400	453	453	0.61	120	22.92	23.3	0.91
(Badaruddin et al., 1999)	1991	Dinajpur, Bangladesh	Silt loam	Wheat	CK	2.41	53	500	553	553	0.44	120	20.11	23.3	0.91
(Badaruddin et al., 1999)	1991	WadMedani, Sudan	Silt	Wheat	CK	4.06	0	600	600	600	0.68	86	47.21	25.5	0.34
(Badaruddin et al., 1999)	1991	WadMedani, Sudan	Silt	Wheat	S	4.6	0	600	600	600	0.77	186	24.72	25.5	0.34
(Badaruddin et al., 1999)	1991	WadMedani, Sudan	Silt	Wheat	CK	3.84	0	600	600	600	0.64	129	29.73	25.5	0.34
(Badaruddin et al., 1999)	1991	WadMedani, Sudan	Silt	Wheat	S	4.35	0	600	600	600	0.72	86	50.55	25.5	0.34
(Badaruddin et al., 1999)	1991	WadMedani, Sudan	Silt	Wheat	CK	4.36	0	600	600	600	0.73	86	50.73	25.5	0.34
(Badaruddin et al., 1999)	1990	WadMedani, Sudan	Silt	Wheat	CK	3.4	0	425	425	425	0.8	86	39.53	25.5	0.34
(Badaruddin et al., 1999)	1990	WadMedani, Sudan	Silt	Wheat	CK	3.5	0	425	425	425	0.82	129	27.13	25.5	0.34
(Badaruddin et al., 1999)	1990	WadMedani, Sudan	Silt	Wheat	S	3.8	0	425	425	425	0.89	186	20.43	25.5	0.34
(Badaruddin et al., 1999)	1990	WadMedani, Sudan	Silt	Wheat	S	3.7	0	425	425	425	0.87	86	43.02	25.5	0.34
(Badaruddin et al., 1999)	1990	Mexico	Vertisol	Wheat	CK	4.8	15	425	440	440	1.09	200	24	20.54	2.15
(Badaruddin et al., 1999)	1990	Mexico	Vertisol	Wheat	CK	5.2	15	425	440	440	1.18	300	17.33	20.54	2.15
(Badaruddin et al., 1999)	1990	Mexico	Vertisol	Wheat	S	5.5	15	425	440	440	1.25	300	18.33	20.54	2.15
(Badaruddin et al., 1999)	1990	Mexico	Vertisol	Wheat	S	5.1	15	425	440	440	1.16	200	25.5	20.54	2.15
(Badaruddin et al., 1999)	1990	Mexico	Vertisol	Wheat	CK	3.5	70	425	495	495	0.71	200	17.5	20.54	2.15
(Badaruddin et al., 1999)	1990	Mexico	Vertisol	Wheat	CK	4.1	70	425	495	495	0.83	300	13.67	20.54	2.15
(Badaruddin et al., 1999)	1990	Mexico	Vertisol	Wheat	S	4.1	70	425	495	495	0.83	300	13.67	20.54	2.15
(Badaruddin et al., 1999)	1990	Mexico	Vertisol	Wheat	S	3.9	70	425	495	495	0.79	200	19.5	20.54	2.15

(Badaruddin et al., 1999)	1992	Mexico	Vertisol	Wheat	CK	4.9	15	425	440	440	1.11	200	24.5	20.54	2.15
(Badaruddin et al., 1999)	1992	Mexico	Vertisol	Wheat	S	5.9	15	425	440	440	1.34	300	19.67	20.54	2.15
(Badaruddin et al., 1999)	1992	Mexico	Vertisol	Wheat	S	5.2	15	425	440	440	1.18	200	26	20.54	2.15
(Badaruddin et al., 1999)	1992	Mexico	Vertisol	Wheat	CK	5.2	15	425	440	440	1.18	300	17.33	20.54	2.15
(Badaruddin et al., 1999)	1992	Mexico	Vertisol	Wheat	CK	3.7	15	425	440	440	0.84	0	NA	20.54	2.15
(Badaruddin et al., 1999)	1993	Mexico	Vertisol	Wheat	CK	4.3	70	425	495	495	0.87	200	21.5	22.68	2.15
(Badaruddin et al., 1999)	1993	Mexico	Vertisol	Wheat	S	5.5	70	425	495	495	1.11	300	18.33	22.68	2.15
(Badaruddin et al., 1999)	1993	Mexico	Vertisol	Wheat	S	4.6	70	425	495	495	0.93	200	23	22.68	2.15
(Badaruddin et al., 1999)	1993	Mexico	Vertisol	Wheat	CK	5	70	425	495	495	1.01	300	16.67	22.68	2.15
(Badaruddin et al., 1999)	1993	Mexico	Vertisol	Wheat	CK	2.1	70	425	495	495	0.42	0	NA	22.68	2.15
(Li et al., 1999)	1996	Lanzhou, China	Silt loam	Wheat	CK	5.45	142.7	0	142.7	262.9	2.07	90	60.52	18.5	2.24
(Li et al., 1999)	1996	Lanzhou, China	Silt loam	Wheat	P	8.21	142.7	0	142.7	280.8	2.92	90	91.19	18.5	2.24
(Li et al., 1999)	1996	Lanzhou, China	Silt loam	Wheat	P	7.85	142.7	0	142.7	299.1	2.62	90	87.19	18.5	2.24
(Li et al., 1999)	1996	Lanzhou, China	Silt loam	Wheat	P	6.7	142.7	0	142.7	267.4	2.51	90	74.47	18.5	2.24
(Tolk et al., 1999)	1994	Texas, USA	Mulch	Wheat	S	6.91	200	100	300	473	1.48	110	62.82	21.27	1.72
(Tolk et al., 1999)	1994	Texas, USA	No mulch	Wheat	CK	7.04	200	100	300	472	1.49	110	64	21.27	1.72
(Tolk et al., 1999)	1995	Texas, USA	Mulch	Wheat	S	5.92	200	162.5	362.5	412	1.44	140	42.29	21.27	1.72
(Tolk et al., 1999)	1995	Texas, USA	No mulch	Wheat	CK	4.94	200	162.5	362.5	391	1.26	140	35.29	21.27	1.72
(Tolk et al., 1999)	1995	Texas, USA	Mulch	Wheat	S	5.48	200	162.5	362.5	395	1.38	140	39.14	21.27	1.72
(Tolk et al., 1999)	1995	Texas, USA	No mulch	Wheat	CK	4.11	200	162.5	362.5	361	1.14	140	29.36	21.27	1.72
(Tolk et al., 1999)	1995	Texas, USA	Mulch	Wheat	S	6.56	200	162.5	362.5	451	1.45	140	46.86	21.27	1.72
(Tolk et al., 1999)	1995	Texas, USA	No Mulch	Wheat	CK	5.69	200	162.5	362.5	420	1.36	140	40.64	21.27	1.72
(Tolk et al., 1999)	1995	Texas, USA	Mulch	Wheat	S	5.75	200	162.5	362.5	390	1.48	140	41.07	21.27	1.72
(Tolk et al., 1999)	1995	Texas, USA	No mulch	Wheat	CK	5.01	200	162.5	362.5	392	1.27	140	35.79	21.27	1.72
(Sharma et al., 2000)	1993	Himachal Pradesh, India	Silty clay loam	Wheat	S	0.63	294.7	0	294.7	444.7	0.14	120	5.25	16.41	1.5
(Sharma et al., 2000)	1993	Himachal Pradesh, India	Silty clay loam	Wheat	S	0.57	273.2	0	273.2	423.2	0.13	120	4.75	16.41	1.5

(Sharma et al., 2000)	1993	Himachal Pradesh, India	Silty clay loam	Wheat	S	0.6	273.2	0	273.2	423.2	0.14	120	5	16.41	1.5
(Sharma et al., 2000)	1993	Himachal Pradesh, India	Silty clay loam	Wheat	S	0.67	294.7	0	294.7	444.7	0.15	120	5.58	16.41	1.5
(Sharma et al., 2000)	1993	Himachal Pradesh, India	Silty clay loam	Wheat	S	0.52	273.2	0	273.2	423.2	0.12	120	4.33	16.41	1.5
(Sharma et al., 2000)	1993	Himachal Pradesh, India	Silty clay loam	Wheat	S	0.58	273.2	0	273.2	423.2	0.14	120	4.83	16.41	1.5
(Sharma et al., 2000)	1993	Himachal Pradesh, India	Silty clay loam	Wheat	S	0.55	294.7	0	294.7	444.7	0.12	120	4.58	16.41	1.5
(Sharma et al., 2000)	1993	Himachal Pradesh, India	Silty clay loam	Wheat	S	0.44	273.2	0	273.2	423.2	0.1	120	3.67	16.41	1.5
(Sharma et al., 2000)	1993	Himachal Pradesh, India	Silty clay loam	Wheat	S	0.53	273.2	0	273.2	423.2	0.13	120	4.42	16.41	1.5
(Sharma et al., 2000)	1993	Himachal Pradesh, India	Silty clay loam	Wheat	CK	0.36	294.7	0	294.7	444.7	0.08	120	3	16.41	1.5
(Sharma et al., 2000)	1993	Himachal Pradesh, India	Silty clay loam	Wheat	CK	0.42	273.2	0	273.2	423.2	0.1	120	3.5	16.41	1.5
(Sharma et al., 2000)	1993	Himachal Pradesh, India	Silty clay loam	Wheat	CK	0.42	273.2	0	273.2	423.2	0.1	120	3.5	16.41	1.5
(Sharma et al., 2000)	1994	Himachal Pradesh, India	Silty clay loam	Wheat	S	1.92	297.9	0	297.9	447.9	0.43	120	16	16.41	1.5
(Sharma et al., 2000)	1994	Himachal Pradesh, India	Silty clay loam	Wheat	S	1.83	293.6	0	293.6	443.6	0.41	120	15.25	16.41	1.5
(Sharma et al., 2000)	1994	Himachal Pradesh, India	Silty clay loam	Wheat	S	2.87	270.8	0	270.8	420.8	0.68	120	23.92	16.41	1.5
(Sharma et al., 2000)	1994	Himachal Pradesh, India	Silty clay loam	Wheat	S	1.71	297.9	0	297.9	447.9	0.38	120	14.25	16.41	1.5
(Sharma et al., 2000)	1994	Himachal Pradesh, India	Silty clay loam	Wheat	S	2.14	293.6	0	293.6	443.6	0.48	120	17.83	16.41	1.5
(Sharma et al., 2000)	1994	Himachal Pradesh, India	Silty clay loam	Wheat	S	2.96	270.8	0	270.8	420.8	0.7	120	24.67	16.41	1.5
(Sharma et al., 2000)	1994	Himachal Pradesh, India	Silty clay loam	Wheat	S	1.31	297.9	0	297.9	447.9	0.29	120	10.92	16.41	1.5
(Sharma et al., 2000)	1994	Himachal Pradesh, India	Silty clay loam	Wheat	S	1.77	293.6	0	293.6	443.6	0.4	120	14.75	16.41	1.5
(Sharma et al., 2000)	1994	Himachal Pradesh, India	Silty clay loam	Wheat	S	2.86	270.8	0	270.8	420.8	0.68	120	23.83	16.41	1.5
(Sharma et al., 2000)	1994	Himachal Pradesh, India	Silty clay loam	Wheat	CK	1.1	297.9	0	297.9	447.9	0.25	120	9.17	16.41	1.5
(Sharma et al., 2000)	1994	Himachal Pradesh, India	Silty clay loam	Wheat	CK	1.09	293.6	0	293.6	443.6	0.25	120	9.08	16.41	1.5
(Sharma et al., 2000)	1994	Himachal Pradesh, India	Silty clay loam	Wheat	CK	1.75	270.8	0	270.8	420.8	0.42	120	14.58	16.41	1.5
(Sharma et al., 2000)	1995	Himachal Pradesh, India	Silty clay loam	Wheat	S	1.96	245	0	245	395	0.5	120	16.33	16.41	1.5
(Sharma et al., 2000)	1995	Himachal Pradesh, India	Silty clay loam	Wheat	S	2.31	238.2	0	238.2	388.2	0.6	120	19.25	16.41	1.5
(Sharma et al., 2000)	1995	Himachal Pradesh, India	Silty clay loam	Wheat	S	1.93	229.4	0	229.4	379.4	0.51	120	16.08	16.41	1.5
(Sharma et al., 2000)	1995	Himachal Pradesh, India	Silty clay loam	Wheat	S	1.64	245	0	245	395	0.42	120	13.67	16.41	1.5

(Sharma et al., 2000)	1995	Himachal Pradesh, India	Silty clay loam	Wheat	S	2.08	238.2	0	238.2	388.2	0.54	120	17.33	16.41	1.5
(Sharma et al., 2000)	1995	Himachal Pradesh, India	Silty clay loam	Wheat	S	1.58	229.4	0	229.4	379.4	0.42	120	13.17	16.41	1.5
(Sharma et al., 2000)	1995	Himachal Pradesh, India	Silty clay loam	Wheat	CK	0.78	245	0	245	395	0.2	120	6.5	16.41	1.5
(Sharma et al., 2000)	1995	Himachal Pradesh, India	Silty clay loam	Wheat	CK	0.91	238.2	0	238.2	388.2	0.23	120	7.58	16.41	1.5
(Sharma et al., 2000)	1995	Himachal Pradesh, India	Silty clay loam	Wheat	CK	0.86	229.4	0	229.4	379.4	0.23	120	7.17	16.41	1.5
(Sharma et al., 2000)	1995	Himachal Pradesh, India	Silty clay loam	Wheat	S	1.47	245	0	245	395	0.37	120	12.25	16.41	1.5
(Sharma et al., 2000)	1995	Himachal Pradesh, India	Silty clay loam	Wheat	S	1.35	238.2	0	238.2	388.2	0.35	120	11.25	16.41	1.5
(Sharma et al., 2000)	1995	Himachal Pradesh, India	Silty clay loam	Wheat	S	1.29	229.4	0	229.4	379.4	0.34	120	10.75	16.41	1.5
(Sharma et al., 2000)	1996	Himachal Pradesh, India	Silty clay loam	Wheat	S	2.69	229.1	0	229.1	379.1	0.71	120	22.42	16.41	1.5
(Sharma et al., 2000)	1996	Himachal Pradesh, India	Silty clay loam	Wheat	S	2.8	226.8	0	226.8	376.8	0.74	120	23.33	16.41	1.5
(Sharma et al., 2000)	1996	Himachal Pradesh, India	Silty clay loam	Wheat	S	2.84	217.4	0	217.4	367.4	0.77	120	23.67	16.41	1.5
(Sharma et al., 2000)	1996	Himachal Pradesh, India	Silty clay loam	Wheat	S	2.6	229.1	0	229.1	379.1	0.69	120	21.67	16.41	1.5
(Sharma et al., 2000)	1996	Himachal Pradesh, India	Silty clay loam	Wheat	S	2.62	226.8	0	226.8	376.8	0.7	120	21.83	16.41	1.5
(Sharma et al., 2000)	1996	Himachal Pradesh, India	Silty clay loam	Wheat	S	2.62	217.4	0	217.4	367.4	0.71	120	21.83	16.41	1.5
(Sharma et al., 2000)	1996	Himachal Pradesh, India	Silty clay loam	Wheat	CK	1.29	229.1	0	229.1	379.1	0.34	120	10.75	16.41	1.5
(Sharma et al., 2000)	1996	Himachal Pradesh, India	Silty clay loam	Wheat	CK	1.16	226.8	0	226.8	376.8	0.31	120	9.67	16.41	1.5
(Sharma et al., 2000)	1996	Himachal Pradesh, India	Silty clay loam	Wheat	CK	1.78	217.4	0	217.4	367.4	0.48	120	14.83	16.41	1.5
(Sharma et al., 2000)	1996	Himachal Pradesh, India	Silty clay loam	Wheat	S	1.4	229.1	0	229.1	379.1	0.37	120	11.67	16.41	1.5
(Sharma et al., 2000)	1996	Himachal Pradesh, India	Silty clay loam	Wheat	S	1.48	226.8	0	226.8	376.8	0.39	120	12.33	16.41	1.5
(Sharma et al., 2000)	1996	Himachal Pradesh, India	Silty clay loam	Wheat	S	1.96	217.4	0	217.4	367.4	0.53	120	16.33	16.41	1.5
(Zhang et al., 2003)	1997	Luancheng, China	Loam	Wheat	CK	5.41	127	0	127	300	1.8	150	36.09	8.29	1.4
(Zhang et al., 2003)	1997	Luancheng, China	Loam	Wheat	CK	6.09	127	60	187	334	1.82	150	40.59	8.29	1.4
(Zhang et al., 2003)	1997	Luancheng, China	Loam	Wheat	CK	5.96	127	120	247	366	1.63	150	39.72	8.29	1.4
(Zhang et al., 2003)	1997	Luancheng, China	Loam	Wheat	CK	5.96	127	120	247	359	1.66	150	39.7	8.29	1.4
(Zhang et al., 2003)	1997	Luancheng, China	Loam	Wheat	CK	5.92	127	180	307	390	1.52	150	39.47	8.29	1.4
(Zhang et al., 2003)	1997	Luancheng, China	Loam	Wheat	CK	5.24	127	240	367	408	1.28	150	34.93	8.29	1.4

(Zhang et al., 2003)	1997	Luancheng, China	Loam	Wheat	CK	5.25	127	228	355	401	1.31	150	35	8.29	1.4
(Zhang et al., 2003)	1998	Luancheng, China	Loam	Wheat	CK	5.33	60	0	60	323	1.65	150	35.51	8.29	1.4
(Zhang et al., 2003)	1998	Luancheng, China	Loam	Wheat	CK	6.25	60	60	120	368	1.7	150	41.67	8.29	1.4
(Zhang et al., 2003)	1998	Luancheng, China	Loam	Wheat	CK	7	60	120	180	438	1.6	150	46.65	8.29	1.4
(Zhang et al., 2003)	1998	Luancheng, China	Loam	Wheat	CK	7.02	60	120	180	431	1.63	150	46.77	8.29	1.4
(Zhang et al., 2003)	1998	Luancheng, China	Loam	Wheat	CK	7.02	60	180	240	451	1.56	150	46.81	8.29	1.4
(Zhang et al., 2003)	1998	Luancheng, China	Loam	Wheat	CK	6.94	60	240	300	478	1.45	150	46.25	8.29	1.4
(Zhang et al., 2003)	1998	Luancheng, China	Loam	Wheat	CK	6.33	60	275	335	460	1.38	150	42.19	8.29	1.4
(Zhang et al., 2003)	1998	Luancheng, China	Loam	Wheat	S	7.14	80	60	140	367	1.95	150	47.59	8.29	1.4
(Zhang et al., 2003)	1998	Luancheng, China	Loam	Wheat	CK	6.69	80	60	140	390	1.72	150	44.61	8.29	1.4
(Zhang et al., 2003)	1999	Luancheng, China	Loam	Wheat	CK	5.1	54	0	54	293	1.74	150	34.03	8.29	1.4
(Zhang et al., 2003)	1999	Luancheng, China	Loam	Wheat	CK	6.38	54	60	114	357	1.79	150	42.54	8.29	1.4
(Zhang et al., 2003)	1999	Luancheng, China	Loam	Wheat	CK	7.09	54	120	174	414	1.71	150	47.29	8.29	1.4
(Zhang et al., 2003)	1999	Luancheng, China	Loam	Wheat	CK	7.12	54	120	174	404	1.76	150	47.49	8.29	1.4
(Zhang et al., 2003)	1999	Luancheng, China	Loam	Wheat	CK	7.39	54	180	234	443	1.67	150	49.29	8.29	1.4
(Zhang et al., 2003)	1999	Luancheng, China	Loam	Wheat	CK	6.94	54	240	294	488	1.42	150	46.25	8.29	1.4
(Zhang et al., 2003)	1999	Luancheng, China	Loam	Wheat	CK	6.72	54	283	337	463	1.45	150	44.81	8.29	1.4
(Li FM et al.,2004)	1999	Gansu,China	Silt loam	Wheat	CK	2.56	240	56.5	296.5	296.6	0.86	75	34.07	16.05	1.47
(Li FM et al.,2004)	1999	Gansu,China	Silt loam	Wheat	CK	2.44	240	86.5	326.5	336.7	0.72	75	32.48	16.05	1.47
(Li FM et al.,2004)	1999	Gansu,China	Silt loam	Wheat	P	2.46	240	56.5	296.5	301.4	0.81	75	32.8	16.05	1.47
(Li FM et al.,2004)	1999	Gansu,China	Silt loam	Wheat	P	3.31	240	86.5	326.5	361	0.92	75	44.1	16.05	1.47
(Li FM et al.,2004)	2000	Gansu,China	Silt loam	Wheat	CK	0.84	91	0	91	209.4	0.4	75	11.14	16.05	1.47
(Li FM et al.,2004)	2000	Gansu,China	Silt loam	Wheat	CK	1.37	91	30	121	197.2	0.7	75	18.31	16.05	1.47
(Li FM et al.,2004)	2000	Gansu,China	Silt loam	Wheat	P	1.22	91	0	91	202.8	0.59	75	16.24	16.05	1.47
(Li FM et al.,2004)	2000	Gansu,China	Silt loam	Wheat	P	1.79	91	30	121	216.9	0.82	75	23.88	16.05	1.47
(Niu et al., 2004)	2001	Gansu, China	Sandy loam	Wheat	P	7.49	243.5	480	723.5	723.5	1.04	150	49.95	16.05	1.49

(Niu et al., 2004)	2001	Gansu, China	Sandy loam	Wheat	CK	5.87	243.5	480	723.5	723.5	0.81	150	39.15	16.05	1.49
(Niu et al., 2004)	2002	Gansu, China	Sandy loam	Wheat	P	7.72	243.5	480	723.5	723.5	1.07	150	51.49	16.05	1.49
(Niu et al., 2004)	2002	Gansu, China	Sandy loam	Wheat	CK	6.07	243.5	480	723.5	723.5	0.84	150	40.45	16.05	1.49
(Zhang et al., 2004)	1998	Luancheng, China	Loam	Wheat	CK	5.32	63.2	332	395.2	375.2	1.42	132.6	40.12	8.29	1.2
(Zhang et al., 2004)	1998	Luancheng, China	Loam	Wheat	CK	5.33	63.2	319.5	382.7	391.6	1.36	132.6	40.2	8.29	1.2
(Zhang et al., 2004)	1998	Luancheng, China	Loam	Wheat	CK	5.88	63.2	282.3	345.5	390.4	1.51	132.6	44.34	8.29	1.2
(Zhang et al., 2004)	1998	Luancheng, China	Loam	Wheat	CK	5.41	63.2	442.1	505.3	435.3	1.24	132.6	40.8	8.29	1.2
(Zhang et al., 2004)	1998	Luancheng, China	Loam	Wheat	S	2.55	63.2	85	148.2	209.5	1.22	132.6	19.23	8.29	1.2
(Zhang et al., 2004)	1999	Luancheng, China	Loam	Wheat	CK	5.67	53.5	361.5	415	381	1.49	132.6	42.76	8.29	1.2
(Zhang et al., 2004)	1999	Luancheng, China	Loam	Wheat	CK	5.49	53.5	403.9	457.4	419.5	1.31	132.6	41.4	8.29	1.2
(Zhang et al., 2004)	1999	Luancheng, China	Loam	Wheat	CK	5.58	53.5	327.9	381.4	386.7	1.44	132.6	42.08	8.29	1.2
(Zhang et al., 2004)	1999	Luancheng, China	Loam	Wheat	CK	5.31	53.5	484.8	538.3	456.7	1.16	132.6	40.05	8.29	1.2
(Zhang et al., 2004)	1999	Luancheng, China	Loam	Wheat	S	3.55	53.5	170	223.5	221.4	1.6	132.6	26.77	8.29	1.2
(Zhang et al., 2004)	2000	Luancheng, China	Loam	Wheat	CK	5.22	139.3	314	453.3	403.6	1.29	132.6	39.37	8.29	1.2
(Zhang et al., 2004)	2000	Luancheng, China	Loam	Wheat	CK	5.11	139.3	310	449.3	405.6	1.26	132.6	38.54	8.29	1.2
(Zhang et al., 2004)	2000	Luancheng, China	Loam	Wheat	CK	5	139.3	296.7	436	403.8	1.24	132.6	37.71	8.29	1.2
(Zhang et al., 2004)	2000	Luancheng, China	Loam	Wheat	CK	5.46	139.3	444.7	584	453	1.21	132.6	41.18	8.29	1.2
(Zhang et al., 2004)	2000	Luancheng, China	Loam	Wheat	S	3.33	139.3	160	299.3	277.7	1.2	132.6	25.11	8.29	1.2
(Huang et al., 2005)	1997	Gansu,China	Silt loam	Wheat	CK	0.82	184	0	184	239.2	0.34	120	6.82	16.05	0.85
(Huang et al., 2005)	1997	Gansu,China	Silt loam	Wheat	S	1.24	184	0	184	219.5	0.57	120	10.33	16.05	0.85
(Huang et al., 2005)	1997	Gansu,China	Silt loam	Wheat	CK	1.25	184	15	199	250.6	0.5	120	10.42	16.05	0.85
(Huang et al., 2005)	1997	Gansu,China	Silt loam	Wheat	CK	1.38	184	35	219	260.8	0.53	120	11.53	16.05	0.85
(Huang et al., 2005)	1997	Gansu,China	Silt loam	Wheat	CK	1.65	184	45	229	271.1	0.61	120	13.75	16.05	0.85
(Huang et al., 2005)	1998	Gansu,China	Silt loam	Wheat	CK	2.06	278	0	278	369.6	0.56	120	17.18	16.05	0.85
(Huang et al., 2005)	1998	Gansu,China	Silt loam	Wheat	S	2.6	278	0	278	355.3	0.73	120	21.68	16.05	0.85
(Huang et al., 2005)	1998	Gansu,China	Silt loam	Wheat	CK	2.52	278	10	288	349.9	0.72	120	21	16.05	0.85

(Huang et al., 2005)	1998	Gansu,China	Silt loam	Wheat	CK	2.88	278	20	298	338.8	0.85	120	24.03	16.05	0.85
(Huang et al., 2005)	1998	Gansu,China	Silt loam	Wheat	CK	3.23	278	30	308	364.4	0.89	120	26.92	16.05	0.85
(Li et al., 2005)	1998	Gansu, China	Silt loam	Wheat	CK	2.52	115	65	180	198.7	1.27	90	27.97	16.05	1.17
(Li et al., 2005)	1998	Gansu, China	Silt loam	Wheat	P	2.73	115	65	180	189.1	1.45	90	30.38	16.05	1.17
(Li et al., 2005)	1998	Gansu, China	Silt loam	Wheat	CK	1.27	115	65	180	204.3	0.62	90	14.1	16.05	1.17
(Li et al., 2005)	1998	Gansu, China	Silt loam	Wheat	P	1.63	115	65	180	195.4	0.83	90	18.06	16.05	1.17
(Rahman et al.,2005)	2000	Bangladesh	Sandy loam	Wheat	CK	0.58	2	425	427	427	0.14	0	NA	20	0.81
(Rahman et al.,2005)	2000	Bangladesh	Sandy loam	Wheat	CK	1.93	2	425	427	427	0.45	80	24.13	20	0.81
(Rahman et al.,2005)	2000	Bangladesh	Sandy loam	Wheat	CK	2.36	2	425	427	427	0.55	120	19.67	20	0.81
(Rahman et al.,2005)	2000	Bangladesh	Sandy loam	Wheat	CK	3.06	2	425	427	427	0.72	160	19.13	20	0.81
(Rahman et al.,2005)	2000	Bangladesh	Sandy loam	Wheat	S	0.77	2	425	427	427	0.18	0	NA	20	0.81
(Rahman et al.,2005)	2000	Bangladesh	Sandy loam	Wheat	S	2.83	2	425	427	427	0.66	80	35.38	20	0.81
(Rahman et al.,2005)	2000	Bangladesh	Sandy loam	Wheat	S	3.7	2	425	427	427	0.87	120	30.83	20	0.81
(Rahman et al.,2005)	2000	Bangladesh	Sandy loam	Wheat	S	3.81	2	425	427	427	0.89	160	23.81	20	0.81
(Rahman et al.,2005)	2000	Bangladesh	Sandy loam	Wheat	S	0.79	2	425	427	427	0.19	0	NA	20	0.81
(Rahman et al.,2005)	2000	Bangladesh	Sandy loam	Wheat	S	2.92	2	425	427	427	0.68	80	36.5	20	0.81
(Rahman et al.,2005)	2000	Bangladesh	Sandy loam	Wheat	S	3.93	2	425	427	427	0.92	120	32.75	20	0.81
(Rahman et al.,2005)	2000	Bangladesh	Sandy loam	Wheat	S	3.98	2	425	427	427	0.93	160	24.88	20	0.81
(Rahman et al.,2005)	2001	Bangladesh	Sandy loam	Wheat	CK	0.31	115	305	420	420	0.07	0	NA	20	0.81
(Rahman et al.,2005)	2001	Bangladesh	Sandy loam	Wheat	CK	1.52	115	305	420	420	0.36	80	19	20	0.81
(Rahman et al.,2005)	2001	Bangladesh	Sandy loam	Wheat	CK	1.96	115	305	420	420	0.47	120	16.33	20	0.81
(Rahman et al.,2005)	2001	Bangladesh	Sandy loam	Wheat	CK	2.54	115	305	420	420	0.6	160	15.88	20	0.81
(Rahman et al.,2005)	2001	Bangladesh	Sandy loam	Wheat	S	0.67	115	305	420	420	0.16	0	NA	20	0.81
(Rahman et al.,2005)	2001	Bangladesh	Sandy loam	Wheat	S	2.17	115	305	420	420	0.52	80	27.13	20	0.81
(Rahman et al.,2005)	2001	Bangladesh	Sandy loam	Wheat	S	3.74	115	305	420	420	0.89	120	31.17	20	0.81
(Rahman et al.,2005)	2001	Bangladesh	Sandy loam	Wheat	S	3.62	115	305	420	420	0.86	160	22.63	20	0.81

(Rahman et al.,2005)	2001	Bangladesh	Sandy loam	Wheat	S	0.69	115	305	420	420	0.16	0	NA	20	0.81
(Rahman et al.,2005)	2001	Bangladesh	Sandy loam	Wheat	S	2.21	115	305	420	420	0.53	80	27.63	20	0.81
(Rahman et al.,2005)	2001	Bangladesh	Sandy loam	Wheat	S	3.92	115	305	420	420	0.93	120	32.67	20	0.81
(Rahman et al.,2005)	2001	Bangladesh	Sandy loam	Wheat	S	3.73	115	305	420	420	0.89	160	23.31	20	0.81
(Xie et al., 2005)	1990	Gansu, China	sandy silt loam	Wheat	P	3.69	34	100	134	281	1.31	150	24.62	13.43	1.64
(Xie et al., 2005)	1990	Gansu, China	sandy silt loam	Wheat	P	5.96	34	404.52	438.52	567	1.05	150	39.76	13.43	1.64
(Xie et al., 2005)	1990	Gansu, China	sandy silt loam	Wheat	P	6.69	34	507.74	541.74	653	1.02	150	44.58	13.43	1.64
(Xie et al., 2005)	1990	Gansu, China	sandy silt loam	Wheat	P	7.08	34	543.87	577.87	691	1.02	150	47.21	13.43	1.64
(Xie et al., 2005)	1990	Gansu, China	sandy silt loam	Wheat	P	6.74	34	693.55	727.55	744	0.91	150	44.94	13.43	1.64
(Xie et al., 2005)	1990	Gansu, China	sandy silt loam	Wheat	P	6.66	34	776.13	810.13	771	0.86	150	44.42	13.43	1.64
(Xie et al., 2005)	1990	Gansu, China	sandy silt loam	Wheat	CK	2.03	34	100	134	240	0.85	150	13.55	13.43	1.64
(Xie et al., 2005)	1990	Gansu, China	sandy silt loam	Wheat	CK	2.84	34	327.1	361.1	434	0.65	150	18.91	13.43	1.64
(Xie et al., 2005)	1990	Gansu, China	sandy silt loam	Wheat	CK	4.75	34	445.81	479.81	510	0.93	150	31.69	13.43	1.64
(Xie et al., 2005)	1990	Gansu, China	sandy silt loam	Wheat	CK	6.1	34	543.87	577.87	640	0.95	150	40.68	13.43	1.64
(Xie et al., 2005)	1990	Gansu, China	sandy silt loam	Wheat	CK	6.48	34	714.19	748.19	705	0.92	150	43.21	13.43	1.64
(Xie et al., 2005)	1990	Gansu, China	sandy silt loam	Wheat	CK	6.32	34	786.45	820.45	746	0.85	150	42.12	13.43	1.64
(Xie et al., 2005)	1991	Gansu, China	sandy silt loam	Wheat	P	3.25	52	100	152	257	1.26	150	21.66	13.43	1.64
(Xie et al., 2005)	1991	Gansu, China	sandy silt loam	Wheat	P	5.76	52	409.78	461.78	527	1.09	150	38.37	13.43	1.64
(Xie et al., 2005)	1991	Gansu, China	sandy silt loam	Wheat	P	6.32	52	503.45	555.45	636	0.99	150	42.14	13.43	1.64
(Xie et al., 2005)	1991	Gansu, China	sandy silt loam	Wheat	P	6.85	52	548.03	600.03	682	1	150	45.69	13.43	1.64
(Xie et al., 2005)	1991	Gansu, China	sandy silt loam	Wheat	P	6.92	52	636.73	688.73	736	0.94	150	46.15	13.43	1.64
(Xie et al., 2005)	1991	Gansu, China	sandy silt loam	Wheat	P	6.76	52	730.43	782.43	759	0.89	150	45.09	13.43	1.64
(Xie et al., 2005)	1991	Gansu, China	sandy silt loam	Wheat	CK	1.97	52	100	152	210	0.94	150	13.11	13.43	1.64
(Xie et al., 2005)	1991	Gansu, China	sandy silt loam	Wheat	CK	2.94	52	318.96	370.96	412	0.71	150	19.61	13.43	1.64
(Xie et al., 2005)	1991	Gansu, China	sandy silt loam	Wheat	CK	4.48	52	451.9	503.9	492	0.91	150	29.86	13.43	1.64
(Xie et al., 2005)	1991	Gansu, China	sandy silt loam	Wheat	CK	5.84	52	550.46	602.46	617	0.94	150	38.91	13.43	1.64

(Xie et al., 2005)	1991	Gansu, China	sandy silt loam	Wheat	CK	6.26	52	707.91	759.91	694	0.9	150	41.7	13.43	1.64
(Xie et al., 2005)	1991	Gansu, China	sandy silt loam	Wheat	CK	6.43	52	777.04	829.04	752	0.85	150	42.85	13.43	1.64
(Chen et al., 2007)	2000	Luancheng, China	Loam	Wheat	CK	7.31	85.5	205	290.5	483.77	1.51	150	48.7	9.08	1.7
(Chen et al., 2007)	2000	Luancheng, China	Loam	Wheat	S	7.29	85.5	210	295.5	461.52	1.58	150	48.61	9.08	1.7
(Chen et al., 2007)	2000	Luancheng, China	Loam	Wheat	S	6.87	85.5	210	295.5	443.42	1.55	150	45.82	9.08	1.7
(Chen et al., 2007)	2001	Luancheng, China	Loam	Wheat	CK	5.01	135.2	170	305.2	400.48	1.25	150	33.37	9.08	1.7
(Chen et al., 2007)	2001	Luancheng, China	Loam	Wheat	S	4.57	135.2	189	324.2	384.12	1.19	150	30.47	9.08	1.7
(Chen et al., 2007)	2001	Luancheng, China	Loam	Wheat	S	4.78	135.2	228	363.2	394.96	1.21	150	31.86	9.08	1.7
(Chen et al., 2007)	2002	Luancheng, China	Loam	Wheat	CK	3.84	171.4	210	381.4	365.43	1.05	150	25.58	9.08	1.7
(Chen et al., 2007)	2002	Luancheng, China	Loam	Wheat	S	3.7	171.4	227	398.4	366.34	1.01	150	24.67	9.08	1.7
(Chen et al., 2007)	2002	Luancheng, China	Loam	Wheat	S	3.49	171.4	227	398.4	345.45	1.01	150	23.26	9.08	1.7
(Chen et al., 2007)	2003	Luancheng, China	Loam	Wheat	CK	6.64	214.1	145	359.1	445.77	1.49	150	44.28	9.08	1.7
(Chen et al., 2007)	2003	Luancheng, China	Loam	Wheat	S	6.12	214.1	145	359.1	419.32	1.46	150	40.81	9.08	1.7
(Chen et al., 2007)	2003	Luancheng, China	Loam	Wheat	S	6.47	214.1	145	359.1	431.4	1.5	150	43.14	9.08	1.7
(Chen et al., 2007)	2004	Luancheng, China	Loam	Wheat	CK	5.33	114.8	207	321.8	394.67	1.35	150	35.52	9.08	1.7
(Chen et al., 2007)	2004	Luancheng, China	Loam	Wheat	S	5.14	114.8	207	321.8	389.24	1.32	150	34.25	9.08	1.7
(Chen et al., 2007)	2004	Luancheng, China	Loam	Wheat	S	4.72	114.8	207	321.8	360.08	1.31	150	31.45	9.08	1.7
(Zhang et al., 2007)	2002	Shaanxi, China	Silt loam	Wheat	S	4.07	208	0	208	228	1.78	180	22.61	9.06	1.48
(Zhang et al., 2007)	2002	Shaanxi, China	Silt loam	Wheat	CK	3.76	208	0	208	240	1.57	180	20.89	9.06	1.48
(Zhang et al., 2007)	2002	Shaanxi, China	Silt loam	Wheat	CK	3.47	208	0	208	226	1.54	180	19.28	9.06	1.48
(Zhang et al., 2007)	2003	Shaanxi, China	Silt loam	Wheat	S	3.01	216	0	216	257	1.17	180	16.72	9.06	1.48
(Zhang et al., 2007)	2003	Shaanxi, China	Silt loam	Wheat	CK	2.51	216	0	216	245	1.02	180	13.94	9.06	1.48
(Zhang et al., 2007)	2003	Shaanxi, China	Silt loam	Wheat	CK	2.39	216	0	216	229	1.04	180	13.28	9.06	1.48
(Zhang et al., 2007)	2004	Shaanxi, China	Silt loam	Wheat	S	5.47	349	0	349	347	1.58	180	30.39	9.06	1.48
(Zhang et al., 2007)	2004	Shaanxi, China	Silt loam	Wheat	CK	6.33	349	0	349	368	1.72	180	35.17	9.06	1.48
(Zhang et al., 2007)	2004	Shaanxi, China	Silt loam	Wheat	CK	5.74	349	0	349	371	1.55	180	31.89	9.06	1.48

(Chakraborty et al., 2008)	2004	New Delhi, India	Sandy loam	Wheat	CK	4.2	52.6	120	172.6	481	0.87	120	34.99	19.71	0.52
(Chakraborty et al., 2008)	2004	New Delhi, India	Sandy loam	Wheat	P	5.02	52.6	120	172.6	421	1.19	120	41.83	19.71	0.52
(Chakraborty et al., 2008)	2004	New Delhi, India	Sandy loam	Wheat	P	4.08	52.6	120	172.6	433	0.94	120	33.99	19.71	0.52
(Chakraborty et al., 2008)	2004	New Delhi, India	Sandy loam	Wheat	S	5.14	52.6	120	172.6	402	1.28	120	42.86	19.71	0.52
(Chakraborty et al., 2008)	2004	New Delhi, India	Sandy loam	Wheat	CK	5.68	52.6	240	292.6	520	1.09	120	47.29	19.71	0.52
(Chakraborty et al., 2008)	2005	New Delhi, India	Sandy loam	Wheat	CK	3.73	23.8	120	143.8	384	0.97	120	31.07	19.71	0.52
(Chakraborty et al., 2008)	2005	New Delhi, India	Sandy loam	Wheat	P	3.87	23.8	120	143.8	400	0.97	120	32.25	19.71	0.52
(Chakraborty et al., 2008)	2005	New Delhi, India	Sandy loam	Wheat	S	4.1	23.8	120	143.8	398	1.03	120	34.17	19.71	0.52
(Chakraborty et al., 2008)	2005	New Delhi, India	Sandy loam	Wheat	CK	4.32	23.8	240	263.8	461	0.94	120	35.99	19.71	0.52
(Zhang SL et al., 2009)	2001	Shaanxi, China	Silt loam	Wheat	CK	3.76	208	0	208	333	1.13	180	20.89	9.63	1.48
(Zhang SL et al., 2009)	2001	Shaanxi, China	Silt loam	Wheat	S	4.07	208	0	208	371	1.1	180	22.61	9.63	1.48
(Zhang SL et al., 2009)	2001	Shaanxi, China	Silt loam	Wheat	CK	1.5	208	0	208	298	0.5	180	8.33	9.63	0.84
(Zhang SL et al., 2009)	2001	Shaanxi, China	Silt loam	Wheat	S	1.53	208	0	208	310	0.49	180	8.5	9.63	0.84
(Zhang SL et al., 2009)	2001	Shaanxi, China	Silt loam	Wheat	CK	2.64	208	0	208	319	0.83	180	14.67	9.63	0.86
(Zhang SL et al., 2009)	2001	Shaanxi, China	Silt loam	Wheat	S	3.12	208	0	208	349	0.89	180	17.33	9.63	0.86
(Zhang SL et al., 2009)	2002	Shaanxi, China	Silt loam	Wheat	CK	2.51	216	0	216	387	0.65	180	13.94	9.63	1.48
(Zhang SL et al., 2009)	2002	Shaanxi, China	Silt loam	Wheat	S	3.01	216	0	216	406	0.74	180	16.72	9.63	1.48
(Zhang SL et al., 2009)	2002	Shaanxi, China	Silt loam	Wheat	CK	1.16	216	0	216	366	0.32	180	6.44	9.63	0.84
(Zhang SL et al., 2009)	2002	Shaanxi, China	Silt loam	Wheat	S	1.51	216	0	216	374	0.4	180	8.39	9.63	0.84
(Zhang SL et al., 2009)	2002	Shaanxi, China	Silt loam	Wheat	CK	4.03	216	0	216	388	1.04	180	22.39	9.63	0.86
(Zhang SL et al., 2009)	2002	Shaanxi, China	Silt loam	Wheat	S	4.49	216	0	216	393	1.14	180	24.94	9.63	0.86
(Zhang SL et al., 2009)	2003	Shaanxi, China	Silt loam	Wheat	CK	6.33	349	0	349	500	1.27	180	35.17	9.63	1.48
(Zhang SL et al., 2009)	2003	Shaanxi, China	Silt loam	Wheat	S	5.47	349	0	349	527	1.04	180	30.39	9.63	1.48
(Zhang SL et al., 2009)	2003	Shaanxi, China	Silt loam	Wheat	CK	2.94	349	0	349	451	0.65	180	16.33	9.63	0.84
(Zhang SL et al., 2009)	2003	Shaanxi, China	Silt loam	Wheat	S	2.74	349	0	349	459	0.6	180	15.22	9.63	0.84
(Zhang SL et al., 2009)	2003	Shaanxi, China	Silt loam	Wheat	CK	5.14	349	0	349	474	1.08	180	28.56	9.63	0.86

(Zhang SL et al., 2009)	2003	Shaanxi, China	Silt loam	Wheat	S	5.31	349	0	349	500	1.06	180	29.5	9.63	0.86
(Balwinder-Singh et al., 2011)	2006	Punjab, India	Clay loam	Wheat	S	4.23	159	75	234	360	1.18	120	35.25	13.78	0.51
(Balwinder-Singh et al., 2011)	2006	Punjab, India	Clay loam	Wheat	S	4.51	159	75	234	341	1.32	120	37.58	13.78	0.51
(Balwinder-Singh et al., 2011)	2006	Punjab, India	Clay loam	Wheat	CK	3.89	159	150	309	367	1.06	120	32.42	13.78	0.51
(Balwinder-Singh et al., 2011)	2006	Punjab, India	Clay loam	Wheat	CK	3.99	159	75	234	345	1.16	120	33.25	13.78	0.51
(Balwinder-Singh et al., 2011)	2007	Punjab, India	Clay loam	Wheat	S	3.84	88	150	238	363	1.06	120	32	13.78	0.51
(Balwinder-Singh et al., 2011)	2007	Punjab, India	Clay loam	Wheat	S	4.09	88	225	313	400	1.02	120	34.08	13.78	0.51
(Balwinder-Singh et al., 2011)	2007	Punjab, India	Clay loam	Wheat	S	4.34	88	150	238	369	1.18	120	36.17	13.78	0.51
(Balwinder-Singh et al., 2011)	2007	Punjab, India	Clay loam	Wheat	S	3.58	88	150	238	362	0.99	120	29.83	13.78	0.51
(Balwinder-Singh et al., 2011)	2007	Punjab, India	Clay loam	Wheat	S	3.27	88	75	163	306	1.07	120	27.25	13.78	0.51
(Balwinder-Singh et al., 2011)	2007	Punjab, India	Clay loam	Wheat	S	3.92	88	150	238	357	1.1	120	32.67	13.78	0.51
(Balwinder-Singh et al., 2011)	2007	Punjab, India	Clay loam	Wheat	CK	3.62	88	225	313	384	0.94	120	30.17	13.78	0.51
(Balwinder-Singh et al., 2011)	2007	Punjab, India	Clay loam	Wheat	CK	3.98	88	225	313	404	0.99	120	33.17	13.78	0.51
(Balwinder-Singh et al., 2011)	2007	Punjab, India	Clay loam	Wheat	CK	4.22	88	150	238	350	1.21	120	35.17	13.78	0.51
(Balwinder-Singh et al., 2011)	2007	Punjab, India	Clay loam	Wheat	CK	3.59	88	150	238	354	1.01	120	29.92	13.78	0.51
(Balwinder-Singh et al., 2011)	2007	Punjab, India	Clay loam	Wheat	CK	3.13	88	75	163	290	1.08	120	26.08	13.78	0.51
(Balwinder-Singh et al., 2011)	2007	Punjab, India	Clay loam	Wheat	CK	3.52	88	150	238	353	1	120	29.33	13.78	0.51
(Sharma et al., 2011)	2005	Jammu, India	Sandy loam	Wheat	CK	1.02	156	0	156	156	0.66	60	17.07	16.66	0.59
(Sharma et al., 2011)	2005	Jammu, India	Sandy loam	Wheat	S	1.35	156	0	156	156	0.86	60	22.43	16.66	0.59
(Sharma et al., 2011)	2005	Jammu, India	Sandy loam	Wheat	P	1.46	156	0	156	156	0.93	60	24.27	16.66	0.59
(Liu et al., 2014)	2011	Gansu, China	Silt loam	Wheat	CK	0.76	138.2	0	138.2	226.7	0.34	0	NA	21.61	1.22
(Liu et al., 2014)	2011	Gansu, China	Silt loam	Wheat	CK	0.84	138.2	0	138.2	200.4	0.42	70	12.04	21.61	1.22
(Liu et al., 2014)	2011	Gansu, China	Silt loam	Wheat	CK	0.86	138.2	0	138.2	207.7	0.41	140	6.11	21.61	1.22
(Liu et al., 2014)	2011	Gansu, China	Silt loam	Wheat	CK	0.96	138.2	0	138.2	204.9	0.47	280	3.41	21.61	1.22
(Liu et al., 2014)	2011	Gansu, China	Silt loam	Wheat	CK	1.09	138.2	0	138.2	203.2	0.54	420	2.6	21.61	1.22
(Liu et al., 2014)	2011	Gansu, China	Silt loam	Wheat	CK	1.26	138.2	0	138.2	193	0.65	560	2.25	21.61	1.22

(Chakraborty et al., 2010)	2002	New Delhi, India	Sandy loam	Wheat	CK	1.22	37.8	190	227.8	329	0.37	0	NA	17.92	0.52
(Chakraborty et al., 2010)	2002	New Delhi, India	Sandy loam	Wheat	CK	2.39	37.8	190	227.8	354	0.67	60	39.82	17.92	0.52
(Chakraborty et al., 2010)	2002	New Delhi, India	Sandy loam	Wheat	CK	4.25	37.8	190	227.8	430	0.99	120	35.42	17.92	0.52
(Chakraborty et al., 2010)	2002	New Delhi, India	Sandy loam	Wheat	CK	4.79	37.8	190	227.8	457	1.05	150	31.96	17.92	0.52
(Chakraborty et al., 2010)	2002	New Delhi, India	Sandy loam	Wheat	P	1.34	37.8	190	227.8	298	0.45	0	NA	17.92	0.52
(Chakraborty et al., 2010)	2002	New Delhi, India	Sandy loam	Wheat	P	2.88	37.8	190	227.8	323	0.89	60	48.03	17.92	0.52
(Chakraborty et al., 2010)	2002	New Delhi, India	Sandy loam	Wheat	P	4.34	37.8	190	227.8	350	1.24	120	36.2	17.92	0.52
(Chakraborty et al., 2010)	2002	New Delhi, India	Sandy loam	Wheat	P	4.98	37.8	190	227.8	441	1.13	150	33.21	17.92	0.52
(Chakraborty et al., 2010)	2002	New Delhi, India	Sandy loam	Wheat	S	1.73	37.8	190	227.8	288	0.6	0	NA	17.92	0.52
(Chakraborty et al., 2010)	2002	New Delhi, India	Sandy loam	Wheat	S	2.85	37.8	190	227.8	320	0.89	60	47.47	17.92	0.52
(Chakraborty et al., 2010)	2002	New Delhi, India	Sandy loam	Wheat	S	4.87	37.8	190	227.8	404	1.21	120	40.59	17.92	0.52
(Chakraborty et al., 2010)	2002	New Delhi, India	Sandy loam	Wheat	S	5.14	37.8	190	227.8	431	1.19	150	34.29	17.92	0.52
(Chakraborty et al., 2010)	2002	New Delhi, India	Sandy loam	Wheat	S	1.54	37.8	190	227.8	292	0.53	0	NA	17.92	0.52
(Chakraborty et al., 2010)	2002	New Delhi, India	Sandy loam	Wheat	S	3.18	37.8	190	227.8	364	0.87	60	52.98	17.92	0.52
(Chakraborty et al., 2010)	2002	New Delhi, India	Sandy loam	Wheat	S	5.03	37.8	190	227.8	429	1.22	120	41.93	17.92	0.52
(Chakraborty et al., 2010)	2002	New Delhi, India	Sandy loam	Wheat	S	5.4	37.8	190	227.8	412	1.26	150	35.99	17.92	0.52
(Chakraborty et al., 2010)	2003	New Delhi, India	Sandy loam	Wheat	CK	0.98	73.5	190	263.5	313	0.31	0	NA	17.92	0.52
(Chakraborty et al., 2010)	2003	New Delhi, India	Sandy loam	Wheat	CK	2.08	73.5	190	263.5	338	0.61	60	34.63	17.92	0.52
(Chakraborty et al., 2010)	2003	New Delhi, India	Sandy loam	Wheat	CK	3.75	73.5	190	263.5	414	0.91	120	31.24	17.92	0.52
(Chakraborty et al., 2010)	2003	New Delhi, India	Sandy loam	Wheat	CK	4.17	73.5	190	263.5	461	0.91	150	27.81	17.92	0.52
(Chakraborty et al., 2010)	2003	New Delhi, India	Sandy loam	Wheat	P	1.3	73.5	190	263.5	334	0.39	0	NA	17.92	0.52
(Chakraborty et al., 2010)	2003	New Delhi, India	Sandy loam	Wheat	P	2.66	73.5	190	263.5	340	0.78	60	44.25	17.92	0.52
(Chakraborty et al., 2010)	2003	New Delhi, India	Sandy loam	Wheat	P	3.78	73.5	190	263.5	362	1.04	120	31.49	17.92	0.52
(Chakraborty et al., 2010)	2003	New Delhi, India	Sandy loam	Wheat	P	4.5	73.5	190	263.5	445	1.01	150	30.02	17.92	0.52
(Chakraborty et al., 2010)	2003	New Delhi, India	Sandy loam	Wheat	S	1.51	73.5	190	263.5	292	0.52	0	NA	17.92	0.52
(Chakraborty et al., 2010)	2003	New Delhi, India	Sandy loam	Wheat	S	2.73	73.5	190	263.5	304	0.9	60	45.53	17.92	0.52

(Chakraborty et al., 2010)	2003	New Delhi, India	Sandy loam	Wheat	S	4.24	73.5	190	263.5	388	1.09	120	35.31	17.92	0.52
(Chakraborty et al., 2010)	2003	New Delhi, India	Sandy loam	Wheat	S	4.47	73.5	190	263.5	434	1.03	150	29.83	17.92	0.52
(Chakraborty et al., 2010)	2003	New Delhi, India	Sandy loam	Wheat	S	1.34	73.5	190	263.5	306	0.44	0	NA	17.92	0.52
(Chakraborty et al., 2010)	2003	New Delhi, India	Sandy loam	Wheat	S	3.02	73.5	190	263.5	348	0.87	60	50.35	17.92	0.52
(Chakraborty et al., 2010)	2003	New Delhi, India	Sandy loam	Wheat	S	4.38	73.5	190	263.5	396	1.11	120	36.48	17.92	0.52
(Chakraborty et al., 2010)	2003	New Delhi, India	Sandy loam	Wheat	S	4.7	73.5	190	263.5	413	1.14	150	31.31	17.92	0.52
(Gao et al, 2009)	2003	Shaanxi, China	Silt loam	Wheat	CK	2.14	255	0	255	255	0.84	120	17.85	10.85	1.14
(Gao et al, 2009)	2003	Shaanxi, China	Silt loam	Wheat	S	2.27	255	0	255	255	0.89	120	18.88	10.85	1.14
(Gao et al, 2009)	2003	Shaanxi, China	Silt loam	Wheat	P	3.16	255	0	255	255	1.24	120	26.3	10.85	1.14
(Gao et al, 2009)	2003	Shaanxi, China	Silt loam	Wheat	P	3.04	255	0	255	255	1.19	120	25.3	10.85	1.14
(Gao et al, 2009)	2004	Shaanxi, China	Silt loam	Wheat	CK	3.95	235	0	235	235	1.68	120	32.92	10.85	1.14
(Gao et al, 2009)	2004	Shaanxi, China	Silt loam	Wheat	S	3.38	235	0	235	235	1.44	120	28.13	10.85	1.14
(Gao et al, 2009)	2004	Shaanxi, China	Silt loam	Wheat	P	4.01	235	0	235	235	1.71	120	33.4	10.85	1.14
(Gao et al, 2009)	2004	Shaanxi, China	Silt loam	Wheat	P	3.11	235	0	235	235	1.32	120	25.93	10.85	1.14
(Gao et al, 2009)	2005	Shaanxi, China	Silt loam	Wheat	CK	3.84	249	0	249	249	1.54	120	31.97	10.85	1.14
(Gao et al, 2009)	2005	Shaanxi, China	Silt loam	Wheat	S	4.38	249	0	249	249	1.76	120	36.49	10.85	1.14
(Gao et al, 2009)	2005	Shaanxi, China	Silt loam	Wheat	P	4.86	249	0	249	249	1.95	120	40.47	10.85	1.14
(Gao et al, 2009)	2005	Shaanxi, China	Silt loam	Wheat	P	4.67	249	0	249	249	1.87	120	38.9	10.85	1.14
(Liu et al., 2005)	2001	Chengdu, China	Loam	Wheat	CK	5.77	211	0	211	411	1.4	120	48.08	23.84	3.97
(Liu et al., 2005)	2001	Chengdu, China	Loam	Wheat	CK	4.9	211	0	211	411	1.19	120	40.83	23.84	3.97
(Liu et al., 2005)	2001	Chengdu, China	Loam	Wheat	S	5.21	211	0	211	411	1.27	120	43.42	23.84	3.97
(Liu et al., 2005)	2001	Chengdu, China	Loam	Wheat	S	5.29	211	0	211	411	1.29	120	44.08	23.84	3.97
(Liu et al., 2005)	2001	Chengdu, China	Loam	Wheat	CK	4.62	211	0	211	411	1.12	120	38.5	12.17	3.97
(Fan et al., 2005)	2000	Chengdu, China	Sandy loam	Wheat	CK	2.96	114.3	0	114.3	314.3	0.94	0	NA	12.17	1.84
(Fan et al., 2005)	2000	Chengdu, China	Sandy loam	Wheat	CK	3.09	114.3	0	114.3	314.3	0.98	0	NA	12.17	1.84
(Fan et al., 2005)	2000	Chengdu, China	Sandy loam	Wheat	S	3.33	114.3	0	114.3	314.3	1.06	0	NA	12.17	1.84

(Fan et al., 2005)	2000	Chengdu, China	Sandy loam	Wheat	CK	4.29	114.3	0	114.3	314.3	1.36	60	71.45	12.17	1.84
(Fan et al., 2005)	2000	Chengdu, China	Sandy loam	Wheat	CK	4.45	114.3	0	114.3	314.3	1.42	60	74.15	12.17	1.84
(Fan et al., 2005)	2000	Chengdu, China	Sandy loam	Wheat	S	4.49	114.3	0	114.3	314.3	1.43	60	74.78	12.17	1.84
(Fan et al., 2005)	2000	Chengdu, China	Sandy loam	Wheat	CK	5.05	114.3	0	114.3	314.3	1.61	120	42.06	12.17	1.84
(Fan et al., 2005)	2000	Chengdu, China	Sandy loam	Wheat	CK	5.25	114.3	0	114.3	314.3	1.67	120	43.78	12.17	1.84
(Fan et al., 2005)	2000	Chengdu, China	Sandy loam	Wheat	S	5.96	114.3	0	114.3	314.3	1.9	120	49.69	12.17	1.84
(Fan et al., 2005)	2000	Chengdu, China	Sandy loam	Wheat	CK	4.58	114.3	0	114.3	314.3	1.46	180	25.47	12.17	1.84
(Fan et al., 2005)	2000	Chengdu, China	Sandy loam	Wheat	CK	4.77	114.3	0	114.3	314.3	1.52	180	26.49	12.17	1.84
(Fan et al., 2005)	2000	Chengdu, China	Sandy loam	Wheat	S	4.86	114.3	0	114.3	314.3	1.55	180	27.01	12.17	1.84
(Fan et al., 2005)	2001	Chengdu, China	Sandy loam	Wheat	CK	3.03	149.3	0	149.3	349.3	0.87	0	NA	12.17	1.84
(Fan et al., 2005)	2001	Chengdu, China	Sandy loam	Wheat	CK	2.6	149.3	0	149.3	349.3	0.74	0	NA	12.17	1.84
(Fan et al., 2005)	2001	Chengdu, China	Sandy loam	Wheat	S	3.49	149.3	0	149.3	349.3	1	0	NA	12.17	1.84
(Fan et al., 2005)	2001	Chengdu, China	Sandy loam	Wheat	CK	4.26	149.3	0	149.3	349.3	1.22	60	71.05	12.17	1.84
(Fan et al., 2005)	2001	Chengdu, China	Sandy loam	Wheat	CK	3.55	149.3	0	149.3	349.3	1.02	60	59.13	12.17	1.84
(Fan et al., 2005)	2001	Chengdu, China	Sandy loam	Wheat	S	4.43	149.3	0	149.3	349.3	1.27	60	73.77	12.17	1.84
(Fan et al., 2005)	2001	Chengdu, China	Sandy loam	Wheat	CK	4.86	149.3	0	149.3	349.3	1.39	120	40.52	12.17	1.84
(Fan et al., 2005)	2001	Chengdu, China	Sandy loam	Wheat	CK	4.45	149.3	0	149.3	349.3	1.27	120	37.07	12.17	1.84
(Fan et al., 2005)	2001	Chengdu, China	Sandy loam	Wheat	S	4.48	149.3	0	149.3	349.3	1.28	120	37.33	12.17	1.84
(Fan et al., 2005)	2001	Chengdu, China	Sandy loam	Wheat	CK	4.84	149.3	0	149.3	349.3	1.39	180	26.88	12.17	1.84
(Fan et al., 2005)	2001	Chengdu, China	Sandy loam	Wheat	CK	4.14	149.3	0	149.3	349.3	1.19	180	23.02	12.17	1.84
(Fan et al., 2005)	2001	Chengdu, China	Sandy loam	Wheat	S	4.36	149.3	0	149.3	349.3	1.25	180	24.24	12.17	1.84
(Fan et al., 2005)	2002	Chengdu, China	Sandy loam	Wheat	CK	3.1	156.3	0	156.3	356.3	0.87	0	NA	12.17	1.84
(Fan et al., 2005)	2002	Chengdu, China	Sandy loam	Wheat	CK	3.44	156.3	0	156.3	356.3	0.97	0	NA	12.17	1.84
(Fan et al., 2005)	2002	Chengdu, China	Sandy loam	Wheat	S	3.86	156.3	0	156.3	356.3	1.08	0	NA	12.17	1.84
(Fan et al., 2005)	2002	Chengdu, China	Sandy loam	Wheat	CK	4.38	156.3	0	156.3	356.3	1.23	60	73	12.17	1.84
(Fan et al., 2005)	2002	Chengdu, China	Sandy loam	Wheat	CK	4.51	156.3	0	156.3	356.3	1.27	60	75.22	12.17	1.84

(Fan et al., 2005)	2002	Chengdu, China	Sandy loam	Wheat	S	4.05	156.3	0	156.3	356.3	1.14	60	67.43	12.17	1.84
(Fan et al., 2005)	2002	Chengdu, China	Sandy loam	Wheat	CK	4.37	156.3	0	156.3	356.3	1.23	120	36.4	12.17	1.84
(Fan et al., 2005)	2002	Chengdu, China	Sandy loam	Wheat	CK	3.83	156.3	0	156.3	356.3	1.08	120	31.94	12.17	1.84
(Fan et al., 2005)	2002	Chengdu, China	Sandy loam	Wheat	S	5.13	156.3	0	156.3	356.3	1.44	120	42.74	12.17	1.84
(Fan et al., 2005)	2002	Chengdu, China	Sandy loam	Wheat	CK	4.4	156.3	0	156.3	356.3	1.23	180	24.44	12.17	1.84
(Fan et al., 2005)	2002	Chengdu, China	Sandy loam	Wheat	CK	4.31	156.3	0	156.3	356.3	1.21	180	23.95	12.17	1.84
(Fan et al., 2005)	2002	Chengdu, China	Sandy loam	Wheat	S	4.28	156.3	0	156.3	356.3	1.2	180	23.75	12.17	1.84
(Liu et al., 2003)	1999	Chengdu, China	Sandy loam	Wheat	CK	4.55	223.6	0	223.6	423.6	1.07	60	75.75	12.17	2.12
(Liu et al., 2003)	1999	Chengdu, China	Sandy loam	Wheat	CK	5.61	223.6	0	223.6	423.6	1.32	120	46.75	12.17	2.12
(Liu et al., 2003)	1999	Chengdu, China	Sandy loam	Wheat	CK	4.71	223.6	0	223.6	423.6	1.11	60	78.5	12.17	2.12
(Liu et al., 2003)	1999	Chengdu, China	Sandy loam	Wheat	CK	5.89	223.6	0	223.6	423.6	1.39	120	49.07	12.17	2.12
(Liu et al., 2003)	1999	Chengdu, China	Sandy loam	Wheat	S	6.24	223.6	0	223.6	423.6	1.47	60	104	12.17	2.12
(Liu et al., 2003)	1999	Chengdu, China	Sandy loam	Wheat	S	6.04	223.6	0	223.6	423.6	1.43	120	50.32	12.17	2.12
(Liu et al., 2003)	2000	Chengdu, China	Sandy loam	Wheat	CK	4.84	177	0	177	377	1.28	60	80.63	12.17	2.12
(Liu et al., 2003)	2000	Chengdu, China	Sandy loam	Wheat	CK	5.26	177	0	177	377	1.4	120	43.86	12.17	2.12
(Liu et al., 2003)	2000	Chengdu, China	Sandy loam	Wheat	CK	4.31	177	0	177	377	1.14	60	71.83	12.17	2.12
(Liu et al., 2003)	2000	Chengdu, China	Sandy loam	Wheat	CK	5.43	177	0	177	377	1.44	120	45.28	12.17	2.12
(Liu et al., 2003)	2000	Chengdu, China	Sandy loam	Wheat	S	5.91	177	0	177	377	1.57	60	98.43	12.17	2.12
(Liu et al., 2003)	2000	Chengdu, China	Sandy loam	Wheat	S	5.47	177	0	177	377	1.45	120	45.61	12.17	2.12
(Liu et al., 2003)	2001	Chengdu, China	Sandy loam	Wheat	CK	4.51	252.6	0	252.6	452.6	1	60	75.1	12.17	2.12
(Liu et al., 2003)	2001	Chengdu, China	Sandy loam	Wheat	CK	4.51	252.6	0	252.6	452.6	1	120	37.58	12.17	2.12
(Liu et al., 2003)	2001	Chengdu, China	Sandy loam	Wheat	CK	4.39	252.6	0	252.6	452.6	0.97	60	73.12	12.17	2.12
(Liu et al., 2003)	2001	Chengdu, China	Sandy loam	Wheat	CK	4.77	252.6	0	252.6	452.6	1.05	120	39.74	12.17	2.12
(Liu et al., 2003)	2001	Chengdu, China	Sandy loam	Wheat	S	5.06	252.6	0	252.6	452.6	1.12	60	84.4	12.17	2.12
(Liu et al., 2003)	2001	Chengdu, China	Sandy loam	Wheat	S	4.92	252.6	0	252.6	452.6	1.09	120	40.98	12.17	2.12
(Ram et al., 2013)	2008	Ludhiana, India	Sandy loam	Wheat	CK	5.23	81.2	262.5	343.7	418	1.28	150	34.87	13.78	0.26

(Ram et al., 2013)	2008	Ludhiana, India	Sandy loam	Wheat	S	5.86	81.2	262.5	343.7	407	1.47	150	39.07	13.78	0.26
(Ram et al., 2013)	2008	Ludhiana, India	Sandy loam	Wheat	S	5.84	81.2	262.5	343.7	400	1.51	150	38.93	13.78	0.26
(Ram et al., 2013)	2008	Ludhiana, India	Sandy loam	Wheat	S	5.8	81.2	262.5	343.7	389	1.54	150	38.67	13.78	0.26
(Ram et al., 2013)	2009	Ludhiana, India	Sandy loam	Wheat	CK	4.94	53.9	262.5	316.4	480	1.04	150	32.93	13.78	0.26
(Ram et al., 2013)	2009	Ludhiana, India	Sandy loam	Wheat	S	5.78	53.9	262.5	316.4	474	1.24	150	38.53	13.78	0.26
(Ram et al., 2013)	2009	Ludhiana, India	Sandy loam	Wheat	S	5.94	53.9	262.5	316.4	466	1.29	150	39.6	13.78	0.26
(Ram et al., 2013)	2009	Ludhiana, India	Sandy loam	Wheat	S	6.04	53.9	262.5	316.4	458	1.34	150	40.27	13.78	0.26
(Ram et al., 2013)	2010	Ludhiana, India	Sandy loam	Wheat	CK	4.9	100.2	262.5	362.7	422	1.2	150	32.67	13.78	0.26
(Ram et al., 2013)	2010	Ludhiana, India	Sandy loam	Wheat	S	5.91	100.2	262.5	362.7	415	1.48	150	39.4	13.78	0.26
(Ram et al., 2013)	2010	Ludhiana, India	Sandy loam	Wheat	S	6.17	100.2	262.5	362.7	404	1.57	150	41.13	13.78	0.26
(Ram et al., 2013)	2010	Ludhiana, India	Sandy loam	Wheat	S	6.26	100.2	262.5	362.7	401	1.62	150	41.73	13.78	0.26
(Ram et al., 2012)	2003	Ludhiana, India	Sandy loam	Wheat	CK	5.34	100	476	576	576	0.09	120	44.5	12	0.72
(Ram et al., 2012)	2003	Ludhiana, India	Sandy loam	Wheat	CK	5.3	100	478	578	578	0.92	120	44.17	12	0.72
(Ram et al., 2012)	2003	Ludhiana, India	Sandy loam	Wheat	CK	5.32	100	477	577	577	0.92	120	44.33	12	0.72
(Ram et al., 2012)	2003	Ludhiana, India	Sandy loam	Wheat	S	5.37	100	475	575	575	0.94	120	44.75	12	0.72
(Ram et al., 2012)	2003	Ludhiana, India	Sandy loam	Wheat	CK	5.33	100	478	578	578	0.93	120	44.42	12	0.72
(Ram et al., 2012)	2003	Ludhiana, India	Sandy loam	Wheat	CK	5.33	100	366	466	466	1.15	120	44.42	12	0.72
(Ram et al., 2012)	2003	Ludhiana, India	Sandy loam	Wheat	CK	5.21	100	367	467	467	1.12	120	43.42	12	0.72
(Ram et al., 2012)	2003	Ludhiana, India	Sandy loam	Wheat	S	5.31	100	365	465	465	1.14	120	44.25	12	0.72
(Khaledian et al., 2012)	2004	France	Loamy	Wheat	CK	6.3	229	50	279	436	1.44	151	41.72	12.16	1.34
(Khaledian et al., 2012)	2004	France	Loamy	Wheat	S	3.1	224	36	260	318	0.97	121	25.62	12.16	1.34
(Khaledian et al., 2012)	2005	France	Loamy	Wheat	CK	5.98	71	93	164	419	1.43	160	37.38	12.16	1.34
(Khaledian et al., 2012)	2005	France	Loamy	Wheat	S	2.95	71	90	161	359	0.82	140	21.07	12.16	1.34
(Hu et al., 2012)	2009	Gansu, China	Silt loam	Wheat	CK	1.26	237	0	237	247	0.51	34.5	36.58	19.16	1.36
(Hu et al., 2012)	2009	Gansu, China	Silt loam	Wheat	P	2.46	237	0	237	247	0.99	34.5	71.16	19.16	1.36
(Hu et al., 2012)	2010	Gansu, China	Silt loam	Wheat	CK	1.35	307.7	0	307.7	387.7	0.35	34.5	38.99	19.16	1.36

(Hu et al., 2012)	2010	Gansu, China	Silt loam	Wheat	P	2.33	307.7	0	307.7	337.7	0.69	34.5	67.6	19.16	1.36
(Balwinder-Singh et al., 2011a)	2007	Ludhiana, India	Clay loam	Wheat	S	4.5	159	75	234	340	1.92	120	37.5	13.78	0.06
(Balwinder-Singh et al., 2011a)	2007	Ludhiana, India	Clay loam	Wheat	CK	4	159	75	234	345	1.71	120	33.33	13.78	0.06
(Balwinder-Singh et al., 2011a)	2008	Ludhiana, India	Clay loam	Wheat	S	4.1	88	225	313	400	1.31	120	34.17	13.78	0.06
(Balwinder-Singh et al., 2011a)	2008	Ludhiana, India	Clay loam	Wheat	CK	4	88	225	313	404	1.28	120	33.33	13.78	0.06
(Li et al., 2008)	2002	Shandong, China	Loam	Wheat	CK	4.99	222.2	0	222.2	287.5	2.25	138	36.16	9.63	1.31
(Li et al., 2008)	2002	Shandong, China	Loam	Wheat	CK	5.29	222.2	60	282.2	334.4	1.87	138	38.31	9.63	1.31
(Li et al., 2008)	2002	Shandong, China	Loam	Wheat	CK	5.41	222.2	120	342.2	398.9	1.58	138	39.17	9.63	1.31
(Li et al., 2008)	2002	Shandong, China	Loam	Wheat	CK	5.99	222.2	180	402.2	451.7	1.49	138	43.38	9.63	1.31
(Li et al., 2008)	2002	Shandong, China	Loam	Wheat	S	4.6	222.2	0	222.2	278.4	2.07	138	33.33	9.63	1.31
(Li et al., 2008)	2002	Shandong, China	Loam	Wheat	S	4.67	222.2	60	282.2	335.2	1.65	138	33.82	9.63	1.31
(Li et al., 2008)	2002	Shandong, China	Loam	Wheat	S	5.01	222.2	120	342.2	400.6	1.46	138	36.32	9.63	1.31
(Li et al., 2008)	2002	Shandong, China	Loam	Wheat	S	5.54	222.2	180	402.2	437.2	1.38	138	40.14	9.63	1.31
(Glab et al., 2008)	2005	Poland	Silt loam	Wheat	CK	5.54	352.67	0	352.67	352.67	1.57	60	92.33	6.31	2.24
(Glab et al., 2008)	2005	Poland	Silt loam	Wheat	S	6.79	352.67	0	352.67	352.67	1.93	60	113.17	6.31	2.24
(Glab et al., 2008)	2005	Poland	Silt loam	Wheat	CK	6.53	352.67	0	352.67	352.67	1.85	60	108.83	6.31	2.24
(Glab et al., 2008)	2005	Poland	Silt loam	Wheat	S	7	352.67	0	352.67	352.67	1.98	60	116.67	6.31	2.24
(Landau et al., 2007)	1997	Israel	Sandy loam	Wheat	S	0.99	207	0	207	207	0.48	75	13.25	11.2	0.46
(Landau et al., 2007)	1997	Israel	Sandy loam	Wheat	CK	0.9	207	0	207	207	0.44	75	12.01	11.2	0.46
(Landau et al., 2007)	1999	Israel	Sandy loam	Wheat	S	0.98	173	0	173	173	0.57	75	13.04	11.2	0.46
(Landau et al., 2007)	1999	Israel	Sandy loam	Wheat	CK	0.9	173	0	173	173	0.52	75	12	11.2	0.46
(Landau et al., 2007)	2000	Israel	Sandy loam	Wheat	S	1.51	284	0	284	284	0.53	75	20.13	11.2	0.46
(Landau et al., 2007)	2000	Israel	Sandy loam	Wheat	CK	1.55	284	0	284	284	0.55	75	20.67	11.2	0.46
(Du et al., 2006)	2002	Gansu, China	Silt loam	Wheat	P	7.58	100	120	220	320	2.37	128	59.23	16.05	1.47
(Du et al., 2006)	2002	Gansu, China	Silt loam	Wheat	P	6.83	100	60	160	260	2.63	128	53.33	16.05	1.47
(Du et al., 2006)	2002	Gansu, China	Silt loam	Wheat	P	5.22	100	0	100	200	2.61	128	40.77	16.05	1.47

(Du et al., 2006)	2002	Gansu, China	Silt loam	Wheat	CK	5.57	100	120	220	320	1.74	128	43.5	16.05	1.47
(Du et al., 2006)	2002	Gansu, China	Silt loam	Wheat	CK	4.82	100	60	160	260	1.86	128	37.69	16.05	1.47
(Du et al., 2006)	2002	Gansu, China	Silt loam	Wheat	CK	3.78	100	0	100	200	1.89	128	29.55	16.05	1.47
(Amir et al., 1996)	1989	Israel	Sandy loam	Wheat	CK	1.2	243	0	243	243	0.49	150	8	15.26	0.46
(Amir et al., 1996)	1989	Israel	Sandy loam	Wheat	S	2.51	243	0	243	243	1.03	150	16.73	15.26	0.46
(Amir et al., 1996)	1989	Israel	Sandy loam	Wheat	P	2.53	243	0	243	243	1.04	150	16.87	15.26	0.46
(Amir et al., 1996)	1989	Israel	Sandy loam	Wheat	CK	2.45	243	0	243	243	1.01	150	16.33	15.26	0.46
(Amir et al., 1996)	1989	Israel	Sandy loam	Wheat	S	3.14	243	0	243	243	1.29	150	20.93	15.26	0.46
(Amir et al., 1996)	1989	Israel	Sandy loam	Wheat	P	3.85	243	0	243	243	1.58	150	25.67	15.26	0.46
(Amir et al., 1996)	1991	Israel	Sandy loam	Wheat	CK	5.21	380	0	380	380	1.37	150	34.73	15.26	0.46
(Amir et al., 1996)	1991	Israel	Sandy loam	Wheat	S	4.9	380	0	380	380	1.29	150	32.67	15.26	0.46
(Amir et al., 1996)	1991	Israel	Sandy loam	Wheat	CK	5.63	380	0	380	380	1.48	150	37.53	15.26	0.46
(Amir et al., 1996)	1991	Israel	Sandy loam	Wheat	S	5.09	380	0	380	380	1.34	150	33.93	15.26	0.46
(Amir et al., 1996)	1992	Israel	Sandy loam	Wheat	CK	1.59	251	0	251	251	0.63	150	10.6	15.26	0.46
(Amir et al., 1996)	1992	Israel	Sandy loam	Wheat	S	2.42	251	0	251	251	0.96	150	16.13	15.26	0.46
(Amir et al., 1996)	1992	Israel	Sandy loam	Wheat	CK	2.41	251	0	251	251	0.96	150	16.07	15.26	0.46
(Amir et al., 1996)	1992	Israel	Sandy loam	Wheat	S	5.17	251	0	251	150	3.45	150	34.47	15.26	0.46
(Amir et al., 1996)	1993	Israel	Sandy loam	Wheat	CK	0.93	163	0	163	163	0.57	150	6.2	15.26	0.46
(Amir et al., 1996)	1993	Israel	Sandy loam	Wheat	S	2.82	163	0	163	163	1.73	150	18.8	15.26	0.46
(Amir et al., 1996)	1993	Israel	Sandy loam	Wheat	CK	1.63	163	0	163	163	1	150	10.87	15.26	0.46
(Amir et al., 1996)	1993	Israel	Sandy loam	Wheat	CK	2.21	163	0	163	122	1.81	150	14.73	15.26	0.46
(Amir et al., 1996)	1993	Israel	Sandy loam	Wheat	S	3.11	163	0	163	122	2.55	150	20.73	15.26	0.46
(Li FM et al., 2004a)	1999	Gansu, China	Silt loam	Wheat	CK	2.42	239.9	0	239.9	336.7	0.72	75	32.32	16.05	1.47
(Li FM et al., 2004a)	1999	Gansu, China	Silt loam	Wheat	P	3.14	239.9	0	239.9	335.1	0.94	75	41.85	16.05	1.47
(Li FM et al., 2004a)	1999	Gansu, China	Silt loam	Wheat	P	3.31	239.9	0	239.9	361	0.92	75	44.07	16.05	1.47
(Li FM et al., 2004a)	1999	Gansu, China	Silt loam	Wheat	P	3.12	239.9	0	239.9	342	0.91	75	41.64	16.05	1.47

(Li FM et al., 2004a)	2000	Gansu, China	Silt loam	Wheat	CK	0.83	91.1	59	150.1	209.4	0.4	75	11.12	16.05	1.47
(Li FM et al., 2004a)	2000	Gansu, China	Silt loam	Wheat	P	0.89	91.1	59	150.1	200.4	0.44	75	11.81	16.05	1.47
(Li FM et al., 2004a)	2000	Gansu, China	Silt loam	Wheat	P	1.2	91.1	59	150.1	202.8	0.59	75	16.04	16.05	1.47
(Li FM et al., 2004a)	2000	Gansu, China	Silt loam	Wheat	P	1.2	91.1	59	150.1	203	0.59	75	16.02	16.05	1.47

Supplementary Table S2. Comparison of 3 models with different weighting methods.

Crop	Model	Weight	df	AIC	BIC	logLik
Wheat	1	No weight	6.00	-353.73	-327.69	182.86
	2	Sample size	6.00	232.91	258.95	-110.46
	3	Replicates	6.00	148.88	174.91	-68.44
Maize	1	No weight	6.00	446.01	473.63	-217.00
	2	Sample size	6.00	1501.58	1529.20	-744.79
	3	Replicates	6.00	1083.49	1111.11	-535.75

References

1 Triplett, G. B., Vandoren, D. M. & Schmidt, B. L. Effect of Corn (Zea Mays L) Stover Mulch on No-Tillage Corn Yield and Water Infiltration. *Agronomy Journal* **60**, 236-& (1968).

2 Doss, B. D., King, C. C. & Patterso.Rm. Yield Components and Water Use by Silage Corn with Irrigation, Plastic Mulch, Nitrogen Fertilization, and Plant Spacing. *Agronomy Journal* **62**, 541-& (1970).

3 Smika, D. E. & Ellis, R. Soil Temperature and Wheat Straw Mulch Effects on Wheat Plant Development and Nutrient Concentration. *Agronomy Journal* **63**, 388-& (1971).

4 Bakajev, N. M., Souleymenov, M. K. & Vasjko, I. A. Effects of Straw Mulching in a Summer Fallow-3 Times Spring Wheat Rotation in Northern Kazakhstan, Ussr. *Soil & Tillage Research* **1**, 239-251, doi:Doi 10.1016/0167-1987(80)90027-6 (1981).

5 De, R., Rao, D. V. S. B., Rao, Y. Y., Rao, L. G. G. & Ikramullah, M. Modification of Irrigation Requirement of Wheat through Mulching and Foliar Application of Transpiration Suppressants. *Irrigation Science* **4**, 215-223, doi:Doi 10.1007/Bf00285527 (1983).

6 Sandhu, B. S., Singh, B., Singh, B. & Khera, K. L. Maize Response to Intermittent Submergence, Straw Mulching and Supplemental N-Fertilization in Subtropical Region. *Plant and Soil* **96**, 45-56, doi:Doi 10.1007/Bf02374994 (1986).

7 Sharma, P. K., Kharwara, P. C. & Tewatia, R. K. Residual Soil-Moisture and Wheat Yield in Relation to Mulching and Tillage during Preceding Rain-Fed Crop. *Soil & Tillage Research* **15**, 279-284, doi:Doi 10.1016/0167-1987(90)90084-Q (1990).

8 Sharma, B. R. Effect of Different Tillage Practices, Mulch and Nitrogen on Soil Properties, Growth and Yield of Fodder Maize. *Soil & Tillage Research* **19**, 55-66, doi:Doi 10.1016/0167-1987(91)90109-B (1991).

9 Gill, K. S., Arshad, M. A., Chivunda, B. K., Phiri, B. & Gumbo, M. Influence of Residue Mulch, Tillage and Cultural-Practices on Weed Mass and Corn Yield from 3 Field Experiments. *Soil & Tillage Research* **24**, 211-223, doi:Doi 10.1016/0167-1987(92)90088-S (1992).

10 Sandhu, K. S., Benbi, D. K., Prihar, S. S. & Saggar, S. Dryland Wheat Yield Dependence on Rainfall, Applied N and Mulching in Preceding Maize. *Fertilizer Research* **32**, 229-237, doi:Doi 10.1007/Bf01048785 (1992).

11 Vanderwerf, H. M. G. The Effect of Plastic Mulch and Greenhouse-Raised Seedlings on Yield of Maize. *Journal of Agronomy and Crop Science-Zeitschrift Fur Acker Und Pflanzenbau* **170**, 261-269 (1993).

12 Yibirin, H., Johnson, J. W. & Eckert, D. J. No-Till Corn Production as Affected by Mulch, Potassium Placement, and Soil Exchangeable Potassium. *Agronomy Journal* **85**, 639-644 (1993).

13 Acharya, C. L. & Sharma, P. D. Tillage and Mulch Effects on Soil Physical-Environment, Root-Growth, Nutrient-Uptake and Yield of Maize and Wheat on an Alfisol in North-West India. *Soil & Tillage Research* **32**, 291-302, doi:Doi 10.1016/0167-1987(94)00425-E (1994).

14 Gajri, P. R., Arora, V. K. & Chaudhary, M. R. Maize Growth-Responses to Deep Tillage, Straw Mulching and Farmyard Manure in Coarse-Textured Soils of Nw India. *Soil Use and Management* **10**, 15-20, doi:DOI 10.1111/j.1475-2743.1994.tb00451.x (1994).

15 Wicks, G. A., Crutchfield, D. A. & Burnside, O. C. Influence of Wheat (Triticum-Aestivum) Straw Mulch and Metolachlor on Corn (Zea-Mays) Growth and Yield. *Weed Science* **42**, 141-147 (1994).

16 Yunusa, I. A. M., Sedgley, R. H. & Siddique, K. M. H. Influence of Mulching on the Pattern of Growth and Water-Use by Spring Wheat and Moisture Storage on a Fine Textured Soil. *Plant and Soil* **160**, 119-130, doi:Doi 10.1007/Bf00150353 (1994).

17 Fisher, P. D. An Alternative Plastic Mulching System for Improved Water Management in Dryland Maize Production. *Agricultural Water Management* **27**, 155-166, doi:Doi 10.1016/0378-3774(95)01134-5 (1995).

18 Lal, R. Tillage and Mulching Effects on Maize Yield for 17 Consecutive Seasons on a Tropical Alfisol. *Journal of Sustainable Agriculture* **5**, 79-93, doi:Doi 10.1300/J064v05n04_07 (1995).

19 Amir, J. & Sinclair, T. R. A straw mulch system to allow continuous wheat production in an arid climate. *Field Crops Research* **47**, 21-31, doi:Doi 10.1016/0378-4290(96)00009-3 (1996).

20 Gill, K. S., Gajri, P. R., Chaudhary, M. R. & Singh, B. Tillage, mulch and irrigation effects on corn (Zea mays L) in relation to evaporative demand. *Soil & Tillage Research* **39**, 213-227, doi:Doi 10.1016/S0167-1987(96)01061-6 (1996).

21 Acharya, C. L., Kapur, O. C. & Dixit, S. P. Moisture conservation for rainfed wheat production with alternative mulches and conservation tillage in the hills of north-west India. *Soil & Tillage Research* **46**, 153-163, doi:Doi 10.1016/S0167-1987(98)00030-0 (1998).

22 Niu, J. Y., Gan, Y. T., Zhang, J. W. & Yang, Q. F. Postanthesis dry matter accumulation and redistribution in spring wheat mulched with plastic film. *Crop Science* **38**, 1562-1568 (1998).

23 Sharma, N. K., Singh, P. N., Tyagi, P. C. & Mohan, S. C. Effect of leucaena mulch on soil-water use and wheat yield. *Agricultural Water Management* **35**, 191-200, doi:Doi 10.1016/S0378-3774(97)00047-4 (1998).

24 Badaruddin, M., Reynolds, M. P. & Ageeb, O. A. A. Wheat management in warm environments: Effect of organic and inorganic fertilizers, irrigation frequency, and mulching. *Agronomy Journal* **91**, 975-983 (1999).

25 Li, F. M., Guo, A. H. & Wei, H. Effects of clear plastic film mulch on yield of spring wheat. *Field Crops Research* **63**, 79-86, doi:Doi 10.1016/S0378-4290(99)00027-1 (1999).

26 Tolk, J. A., Howell, T. A. & Evett, S. R. Effect of mulch, irrigation, and soil type on water use and yield of maize. *Soil & Tillage Research* **50**, 137-147, doi:Doi 10.1016/S0167-1987(99)00011-2 (1999).

27 Easson, D. L. & Fearnehough, W. Effects of plastic mulch, sowing date and cultivar on the yield and maturity of forage maize grown under marginal climatic conditions in Northern Ireland. *Grass and Forage Science* **55**, 221-231 (2000).

28 Li, X. Y., Gong, J. D. & Wei, X. H. In-situ rainwater harvesting and gravel mulch combination for corn production in the dry semi-arid region of China. *Journal of Arid Environments* **46**, 371-382 (2000).

29 Sharma, P. K. & Acharya, C. L. Carry-over of residual soil moisture with mulching and conservation tillage practices for sowing of rainfed wheat (Triticum aestivum L.) in north-west India. *Soil & Tillage Research* **57**, 43-52, doi:Doi 10.1016/S0167-1987(00)00141-0 (2000).

30 Li, X. Y., Gong, J. D., Gao, Q. Z. & Li, F. R. Incorporation of ridge and furrow method of rainfall harvesting with mulching for crop production under semiarid conditions. *Agricultural Water Management* **50**, 173-183, doi:Doi 10.1016/S0378-3774(01)00105-6 (2001).

31 Liu, X. J. *et al.* Effects of non-flooded mulching cultivation on crop yield, nutrient uptake and nutrient balance in rice-wheat cropping systems. *Field Crops Research* **83**, 297-311, doi:Doi 10.1016/S0378-4290(03)00079-0 (2003).

32 Zhang, X. Y., Pei, D. & Hu, C. S. Conserving groundwater for irrigation in the North China Plain. *Irrigation Science* **21**, 159-166, doi:DOI 10.1007/s00271-002-0059-x (2003).

33 Li, F. M., Wang, J., Xu, J. Z. & Xu, H. L. Productivity and soil response to plastic film mulching durations for spring wheat on entisols in the semiarid Loess Plateau of China. *Soil & Tillage Research* **78**, 9-20, doi:DOI 10.1016/j.still.2003.12.009 (2004).

34 Li, F. M., Wang, P., Wang, J. & Xu, J. Z. Effects of irrigation before sowing and plastic film mulching on yield and water uptake of spring wheat in semiarid Loess Plateau of China. *Agricultural Water Management* **67**, 77-88, doi:DOI 10.1016/j.agwat.2004.02.001 (2004).

35 Niu, J. Y., Gan, Y. T. & Huang, G. B. Dynamics of root growth in spring wheat mulched with plastic film. *Crop Science* **44**, 1682-1688 (2004).

36 Zhang, Y. Q. *et al.* Effect of soil water deficit on evapotranspiration, crop yield, and water use efficiency in the North China Plain. *Agricultural Water Management* **64**, 107-122, doi:Doi 10.1016/S0378-3774(03)00201-4 (2004).

37 Fan, M. S. *et al.* Interactions between non-flooded mulching cultivation and varying nitrogen inputs in rice-wheat rotations. *Field Crops Research* **91**, 307-318, doi:DOI 10.1016/j.fcr.2004.08.006 (2005).

38 Huang, Y. L., Chen, L. D., Fu, B. J., Huang, Z. L. & Gong, E. The wheat yields and water-use efficiency in the Loess Plateau: straw mulch and irrigation effects. *Agricultural Water Management* **72**, 209-222, doi:DOI 10.1016/j.agwat.2004.09.012 (2005).

39 Li, F. M., Wang, J. & Xu, J. Z. Plastic film mulch effect on spring wheat in a semiarid region. *Journal of Sustainable Agriculture* **25**, 5-17, doi:Doi 10.1300/J064v25n04_03 (2005).

40 Liu, X. J. *et al.* Crop production, nitrogen recovery and water use efficiency in rice-wheat rotation as affected by non-flooded mulching cultivation (NFMC). *Nutrient*

Cycling in Agroecosystems **71**, 289-299, doi:DOI 10.1007/s10705-004-6801-4 (2005).

41 Rahman, M. A., Chikushi, J., Saifizzaman, M. & Lauren, J. G. Rice straw mulching and nitrogen response of no-till wheat following rice in Bangladesh. *Field Crops Research* **91**, 71-81, doi:DOI 10.1016/j.fcr.2004.06.010 (2005).

42 Xie, Z. K., Wang, Y. J. & Li, F. M. Effect of plastic mulching on soil water use and spring wheat yield in and region of northwest China. *Agricultural Water Management* **75**, 71-83, doi:DOI 10.1016/j.agwat.2004.12.014 (2005).

43 Du, Y. J., Li, Z. Z. & Li, W. L. Effect of different water supply regimes on growth and size hierarchy in spring wheat populations under mulched with clear plastic film. *Agricultural Water Management* **79**, 265-279, doi:DOI 10.1016/j.agwat.2005.05.018 (2006).

44 Chen, S. Y., Zhang, X. Y., Pei, D., Sun, H. Y. & Chen, S. L. Effects of straw mulching on soil temperature, evaporation and yield of winter wheat: field experiments on the North China Plain. *Annals of Applied Biology* **150**, 261-268, doi:DOI 10.1111/j.1744-7348.2007.00144.x (2007).

45 Landau, S. *et al.* Grazing, mulching, and removal of wheat straw in a no-till system in a semi-arid environment. *Australian Journal of Agricultural Research* **58**, 907-912, doi:Doi 10.1071/Ar06422 (2007).

46 Zhang, S. L., Lovdahl, L., Grip, H., Jansson, P. E. & Tong, Y. N. Modelling the effects of mulching and fallow cropping on water balance in the Chinese Loess Plateau. *Soil & Tillage Research* **93**, 283-298, doi:DOI 10.1016/j.still.2006.05.002 (2007).

47 Chakraboyty, D. *et al.* Effect of mulching on soil and plant water status, and the growth and yield of wheat (Triticum aestivum L.) in a semi-arid environment. *Agricultural Water Management* **95**, 1323-1334, doi:DOI 10.1016/j.agwat.2008.06.001 (2008).

48 Glab, T. & Kulig, B. Effect of mulch and tillage system on soil porosity under wheat (Triticum aestivum). *Soil & Tillage Research* **99**, 169-178, doi:DOI 10.1016/j.still.2008.02.004 (2008).

49 Li, Q. Q. *et al.* Effects of irrigation and straw mulching on microclimate characteristics and water use efficiency of winter wheat in North China. *Plant Production Science* **11**, 161-170, doi:Doi 10.1626/Pps.11.161 (2008).

50 Gao, Y. J. *et al.* Effects of mulch, N fertilizer, and plant density on wheat yield, wheat nitrogen uptake, and residual soil nitrate in a dryland area of China. *Nutrient Cycling in Agroecosystems* **85**, 109-121, doi:DOI 10.1007/s10705-009-9252-0 (2009).

51 Zhang, S. L. *et al.* Effects of mulching and catch cropping on soil temperature, soil moisture and wheat yield on the Loess Plateau of China. *Soil & Tillage Research* **102**, 78-86, doi:DOI 10.1016/j.still.2008.07.019 (2009).

52 Zhou, L. M., Li, F. M., Jin, S. L. & Song, Y. J. How two ridges and the furrow mulched with plastic film affect soil water, soil temperature and yield of maize on the semiarid Loess Plateau of China. *Field Crops Research* **113**, 41-47, doi:DOI 10.1016/j.fcr.2009.04.005 (2009).

53 Chakraborty, D. *et al.* Synthetic and organic mulching and nitrogen effect on winter wheat (Triticum aestivum L.) in a semi-arid environment. *Agricultural Water*

Management **97**, 738-748, doi:DOI 10.1016/j.agwat.2010.01.006 (2010).

54 Balwinder-Singh, Eberbach, P. L., Humphreys, E. & Kukal, S. S. The effect of rice straw mulch on evapotranspiration, transpiration and soil evaporation of irrigated wheat in Punjab, India. *Agricultural Water Management* **98**, 1847-1855, doi:DOI 10.1016/j.agwat.2011.07.002 (2011).

55 Balwinder-Singh *et al.* Growth, yield and water productivity of zero till wheat as affected by rice straw mulch and irrigation schedule. *Field Crops Research* **121**, 209-225, doi:DOI 10.1016/j.fcr.2010.12.005 (2011).

56 Sharma, P., Abrol, V. & Sharma, R. K. Impact of tillage and mulch management on economics, energy requirement and crop performance in maize-wheat rotation in rainfed subhumid inceptisols, India. *European Journal of Agronomy* **34**, 46-51, doi:DOI 10.1016/j.eja.2010.10.003 (2011).

57 Wang, T. C., Wei, L., Wang, H. Z., Ma, S. C. & Ma, B. L. Responses of rainwater conservation, precipitation-use efficiency and grain yield of summer maize to a furrow-planting and straw-mulching system in northern China. *Field Crops Research* **124**, 223-230, doi:DOI 10.1016/j.fcr.2011.06.014 (2011).

58 Zhang, S. L., Li, P. R., Yang, X. Y., Wang, Z. H. & Chen, X. P. Effects of tillage and plastic mulch on soil water, growth and yield of spring-sown maize. *Soil & Tillage Research* **112**, 92-97, doi:DOI 10.1016/j.still.2010.11.006 (2011).

59 Botha, J. J., Van Rensburg, L. D., Anderson, J. J., van Staden, P. P. & Hensley, M. Improving Maize Production of in-Field Rainwater Harvesting Technique at Glen in South Africa by the Addition of Mulching Practices. *Irrigation and Drainage* **61**, 50-58, doi:Doi 10.1002/Ird.1681 (2012).

60 Hu, B., Jia, Y., Zhao, Z. H., Li, F. M. & Siddique, K. H. M. Soil P availability, inorganic P fractions and yield effect in a calcareous soil with plastic-film-mulched spring wheat. *Field Crops Research* **137**, 221-229, doi:DOI 10.1016/j.fcr.2012.08.014 (2012).

61 Khaledian, M. R., Mailhol, J. C., Ruelle, P. & Mubarak, I. Impacts of Direct Seeding into Mulch on the Yield, Water Use Efficiency and Nitrogen Dynamics of Corn, Sorghum and Durum Wheat. *Irrigation and Drainage* **61**, 398-409, doi:Doi 10.1002/Ird.661 (2012).

62 Mupangwa, W., Twomlow, S. & Walker, S. Reduced tillage, mulching and rotational effects on maize (Zea mays L.), cowpea (Vigna unguiculata (Walp) L.) and sorghum (Sorghum bicolor L. (Moench)) yields under semi-arid conditions. *Field Crops Research* **132**, 139-148, doi:DOI 10.1016/j.fcr.2012.02.020 (2012).

63 Ram, H. *et al.* Agronomic and Economic Evaluation of Permanent Raised Beds, No Tillage and Straw Mulching for an Irrigated Maize-Wheat System in Northwest India. *Experimental Agriculture* **48**, 21-38, doi:Doi 10.1017/S0014479711000809 (2012).

64 Shen, J. Y., Zhao, D. D., Han, H. F., Zhou, X. B. & Li, Q. Q. Effects of straw mulching on water consumption characteristics and yield of different types of summer maize plants. *Plant Soil and Environment* **58**, 161-166 (2012).

65 Abd El-Wahed, M. H. & Ali, E. A. Effect of irrigation systems, amounts of irrigation water and mulching on corn yield, water use efficiency and net profit. *Agricultural Water Management* **120**, 64-71, doi:DOI 10.1016/j.agwat.2012.06.017 (2013).

66 Bu, L. D. *et al.* The effects of mulching on maize growth, yield and water use in a semi-arid region. *Agricultural Water Management* **123**, 71-78, doi:DOI

10.1016/j.agwat.2013.03.015 (2013).
67 Han, J., Jia, Z. K., Han, Q. F. & Zhang, J. Application of Mulching Materials of Rainfall Harvesting System for Improving Soil Water and Corn Growth in Northwest of China. *J Integr Agr* **12**, 1712-1721, doi:Doi 10.1016/S2095-3119(13)60342-1 (2013).
68 Li, R. *et al.* Effects on soil temperature, moisture, and maize yield of cultivation with ridge and furrow mulching in the rainfed area of the Loess Plateau, China. *Agricultural Water Management* **116**, 101-109, doi:DOI 10.1016/j.agwat.2012.10.001 (2013).
69 Li, S. X., Wang, Z. H., Li, S. Q., Gao, Y. & Tian, X. H. Effect of plastic sheet mulch, wheat straw mulch, and maize growth on water loss by evaporation in dryland areas of China. *Agricultural Water Management* **116**, 39-49, doi:DOI 10.1016/j.agwat.2012.10.004 (2013).
70 Ram, H., Dadhwal, V., Vashist, K. K. & Kaur, H. Grain yield and water use efficiency of wheat (Triticum aestivum L.) in relation to irrigation levels and rice straw mulching in North West India. *Agricultural Water Management* **128**, 92-101, doi:DOI 10.1016/j.agwat.2013.06.011 (2013).
71 Gao, Y. H., Xie, Y. P., Jiang, H. Y., Wu, B. & Niu, J. Y. Soil water status and root distribution across the rooting zone in maize with plastic film mulching. *Field Crops Research* **156**, 40-47, doi:DOI 10.1016/j.fcr.2013.10.016 (2014).
72 Liu, C. A. *et al.* Maize yield and water balance is affected by nitrogen application in a film-mulching ridge-furrow system in a semiarid region of China. *European Journal of Agronomy* **52**, 103-111, doi:DOI 10.1016/j.eja.2013.10.001 (2014).
73 Liu, J. L. *et al.* Optimizing Plant Density and Plastic Film Mulch to Increase Maize Productivity and Water-Use Efficiency in Semiarid Areas. *Agronomy Journal* **106**, 1138-1146, doi:DOI 10.2134/agronj13.0582 (2014).
74 Li, S. X., Wang, Z. H., Hu, T. T., Gao, Y. J. & Stewart, B. A. Nitrogen in Dryland Soils of China and Its Management. *Advances in Agronomy, Vol 101* **101**, 123-181, doi:Doi 10.1016/S0065-2113(08)00803-1 (2009).
